# 5′-fluoro(di)phosphate-labeled oligonucleotides are versatile molecular probes for studying nucleic acid secondary structure and interactions by ^19^F NMR

**DOI:** 10.1093/nar/gkaa470

**Published:** 2020-06-09

**Authors:** Marek R Baranowski, Marcin Warminski, Jacek Jemielity, Joanna Kowalska

**Affiliations:** Division of Biophysics, Institute of Experimental Physics, Faculty of Physics, University of Warsaw, Ludwika Pasteura 5, 02-093 Warsaw, Poland; Division of Biophysics, Institute of Experimental Physics, Faculty of Physics, University of Warsaw, Ludwika Pasteura 5, 02-093 Warsaw, Poland; Centre of New Technologies, University of Warsaw, Stefana Banacha 2c, 02-097 Warsaw, Poland; Division of Biophysics, Institute of Experimental Physics, Faculty of Physics, University of Warsaw, Ludwika Pasteura 5, 02-093 Warsaw, Poland

## Abstract

The high sensitivity of ^19^F nucleus to changes in the chemical environment has promoted the use of fluorine-labeled molecular probes to study structure and interactions of nucleic acids by ^19^F NMR. So far, most efforts have focused on incorporating the fluorine atom into nucleobase and ribose moieties using either monomer building blocks for solid-phase synthesis, or nucleoside triphosphates for enzymatic synthesis. Here, we report a simple and efficient synthesis of 5′-fluoromonophosphorylated and 5′-fluorodiphosphorylated oligodeoxyribonucleotides, which combines solid-phase and in-solution synthesis methods and requires only commercially available nucleoside phosphoramidites, followed by their evaluation as ^19^F NMR probes. We confirmed that the fluorine atom at the oligonucleotide 5′ end did not alter the secondary structure of DNA fragments. Moreover, at the same time, it enabled real-time ^19^F NMR monitoring of various DNA-related biophysical processes, such as oligonucleotide hybridization (including mismatch identification), G-quadruplex folding/unfolding and its interactions with thrombin, as well as formation of an *i*-motif structure and its interaction with small-molecule ligands.

## INTRODUCTION

The development of robust methods enabling monitoring of oligonucleotide secondary and tertiary structure transformations, as well as detection of oligonucleotide interactions with (bio)molecules is crucial for improving our understanding of nucleic acid function and the discovery of small molecules targeting nucleic acids. Various spectroscopic methods, including UV-VIS (1), circular dichroism (CD) (2,3), surface plasmon resonance (SPR) (4–6) and fluorescence-based techniques (7–9), have been used to achieve this goal. These methods have found numerous applications, but are nevertheless limited by the need to introduce bulky reporter groups (SPR and fluorescence), narrow scope, and the inability to provide detailed structural information. Nuclear magnetic resonance (NMR) spectroscopy, despite being less sensitive than the above methods, can provide more details about nucleic acid conformational equilibria, conversion mechanisms and interaction sites (10–13). NMR methods also enable the discovery and quantification of intermolecular interactions in the micromolar to millimolar *K*_D_ range (14,15). As a result, NMR techniques have become increasingly popular for the study of nucleic acids. To this end, the ^19^F nucleus in fluorine-labeled nucleic acids exhibits several advantageous NMR-related properties, such as nuclear spin of ^1^/_2_, high-magnetogyric coefficient and 100% abundance, which mean that ^19^F NMR sensitivity amounts to 83% of ^1^H NMR (16). The absence of fluorine in natural compounds makes typical ^19^F NMR spectra simple and easy to analyze. Finally, fluorine nuclei are sensitive to changes in the local chemical environment, so even structurally similar entities can be distinguished by ^19^F NMR. As a result, these features have transformed ^19^F NMR studies on fluorine-labeled nucleic acids into a valuable biophysical tool (17,18).

Several strategies have been developed to obtain fluorine-labeled oligonucleotides for ^19^F NMR experiments. Most of them encompass internal labeling of the oligonucleotide sequence with single or multiple fluorinated nucleotide-derived building blocks. The latter can be incorporated either by (i) solid-supported chemical synthesis or (ii) polymerase-catalyzed enzymatic synthesis in the presence of fluorinated nucleoside triphosphates (19). In the chemical approach, a fluorine atom or a fluorinated substituent is introduced in the ribose or nucleobase moiety of an appropriate phosphoramidite building block through multiple synthetic steps. The fluorinated phosphoramidite is subsequently used in solid-phase synthesis of DNA or RNA. Various modified nucleotides have been synthesized following this methodology (20–24). Virta *et al.* used the click chemistry approach to incorporate the trifluoromethyl (CF_3_) group at the 4′ position of thymidine- and at the 2′-*O* position of cytidine-derived building blocks to study DNA duplex/triplex formation and DNA/RNA secondary structure stabilization by neomycin (25,26). Micura *et al.* reported a 2′-deoxy-2′-trifluoromethylthio (2′-SCF_3_) uridine building block for RNA solid-phase synthesis and prepared 2′-SCF_3_-labeled RNA probes to study RNA–protein and RNA–ligand interactions, conformational transitions and to monitor RNA folding in *Escherichia coli* cell lysates at low probe concentrations. This was achieved despite the fact that 2′-ribose labeling destabilized the RNA double helix by 15°C (1.9 kcal/mol) (27). The same team also reported the introduction of 2′-SCF_3_ into pyrimidine nucleosides to study RNA stability and conformational equilibria of hairpins by ^19^F NMR (28). Enzymatic synthesis of fluorinated DNA probes was reported by Hocek *et al.*, who incorporated trifluoroacetophenone-linked nucleoside triphosphates (uridine, cytidine, 7-deazaadenisine, and 7-deazaguanosine) into DNA to monitor DNA duplex formation and nucleic acid–protein interaction by ^19^F NMR (29).

**Figure 1.**
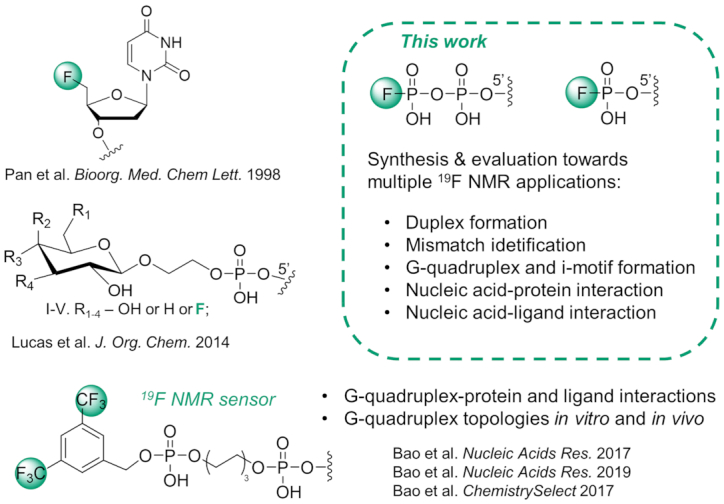
Structures of 5′ end fluorine-labeled oligonucleotides with one example used in ^19^F NMR studies.

The incorporation of fluorine-containing tags at the 5′ end of oligonucleotides (30,31) increases synthetic accessibility owing to the possibility of using post-synthetic functionalization (32,33). However, only a few examples of combining 5′-terminal fluorine-labeling and ^19^F NMR applications have been reported (Figure 1) (34–37). Xu *et al.* introduced 3,5-bis(trifluoromethyl)phenyl phosphoramidite at the 5′ end of oligonucleotides during the final cycle of solid-phase synthesis to functionalize the 5′ end of both RNA and DNA G-quadruplex-forming sequences (Figure 1). The resulting probes were used to study the telomere RNA sequence, detect different G-quadruplex assemblies *in vitro* and in living cells (single-stranded, dimeric and high-order structures) and monitor G-quadruplex–ligand interactions (34,35). More recently, the same 3,5-bis(trifluoromethyl)phenyl moiety was introduced at the 5′ end of the telomere DNA sequence and was applied to study different G-quadruplex topologies in HeLa cells (37).

Despite the above examples of synthesis and ^19^F NMR application of fluorine-labeled oligonucleotide probes, to the best of our knowledge, a universal labeling strategy compatible with multiple applications has not been proposed. We hypothesized that the most effective way to develop such a versatile and synthetically accessible labeling strategy that ensures minimal perturbation of the studied phenomena, was to rely on the 5′-terminal functionalization of oligonucleotides with a structurally compact fluorinated label. One such candidate is the fluorophosphate moiety, which we previously used to develop small nucleotide-derived ^19^F NMR probes to monitor multiple enzymatic activities and protein binding events (36). In a proof of concept experiment, we also demonstrated that short DNA duplex formation could be monitored by ^19^F NMR. Unlike other halogenophosphates or fluorophosphate diesters, fluorophosphate monoesters and phosphoanhydrides are chemically stable in aqueous solutions under a wide pH range. Moreover, the *δ*_F_ chemical shifts of flurophosphates are insensitive to changes in pH or presence of divalent metal ions, but sensitive to intramolecular chemical transformations, which makes them good candidates for ^19^F NMR probes (36). Hence, in the present study, we aimed to systematically investigate the properties, application scope and limitations of oligonucleotides carrying 5′-fluoromonophosphate (FP) or 5′-fluorodiphosphate (FPP) moieties as ^19^F NMR probes. We synthesized several FP- and FPP-functionalized oligodeoxyribonucleotides using straightforward methods that avoided commercially unavailable phosphoramidite building blocks. The ^19^F-labeled oligonucleotides were evaluated as molecular tools to: (i) probe duplex formation and distinguish 5′-terminal mismatches; (ii) monitor G-quadruplex formation; (iii) detect DNA–protein interactions; and (iv) monitor *i*-motif formation and its interactions with small-molecule ligands. We found that the FP and FPP moieties acts as universal ^19^F reporter tags enabling monitoring of a variety of nucleic acids-related phenomena.

## MATERIALS AND METHODS

### Oligonucleotide synthesis

Deoxyribonucleotides (ON1, ON3-ON5, ON9, ON11, ON20-ON25, hTeloC and PON11) were synthesized at a 2–5 μmol scale using an automated DNA/RNA synthesizer (ÄKTA oligopilot 10 plus, GE Healthcare) equipped with a column filled with either PrimerSupport™ 200 T 80s (82 μmol/g), dA 80s (79 μmol/g), dG 80s (78 μmol/g) and dC 80s (84 μmol/g), or PrimerSupport™ 5G dC 350 (360 μmol/g). In the coupling step, 10 equivalents of 0.1 M phosphoramidite (DMT-dG(dmf), DMT-dC(N-acetyl), DMT-dA(N-bz) and DMT-T purchased from ChemGenes) in acetonitrile and 0.3 M BTT in acetonitrile were recirculated through the column for 5 min. If required, the oligodeoxyribonucleotides were 5′-phosphorylated on solid-phase, using 0.1 M bis(2-cyanoethyl)-*N*,*N*-diisopropylphosphoramidite (Sigma-Aldrich) in acetonitrile and 0.3 M BTT. A solution of 3% (v/v) dichloroacetic acid in toluene was used as a detritylation reagent and 0.05 M iodine in pyridine was used for oxidation, 20% (v/v) N-methylimidazole in acetonitrile served as Cap A, while a mixture of 40% (v/v) acetic anhydride and 40% (v/v) pyridine in acetonitrile served as Cap B. After the last cycle of synthesis, DNAs still attached to the solid support were treated with 20% (v/v) diethylamine in acetonitrile to remove 2-cyanoethyl protecting groups. Finally, the solid support was washed with acetonitrile and dried with argon, followed by cleavage and deprotection using a 1:1 (v/v) mixture of 30% ammonia and 40% aqueous methylamine at 40°C for 2 h. Subsequently, the solution was filtered and the support was washed with water several times. Combined filtrates were subjected to evaporation and lyophilization (Supplementary Table S1).

Synthesis of FP analogs (FPON1, FPON3 and FP-hTeloC) proceeded as follows: the 5′-OH group of the oligonucleotide still attached to the solid-phase support (5–20 μmol scale) was reacted with a solution of diphenyl *H*-phosphonate (0.1–0.2 M) in anhydrous pyridine (4 ml) for 2 h; then, it was washed with acetonitrile and finally passed with a solution of 0.1 M triethylammonium bicarbonate buffer (pH 8.0) to obtain stable 5′-*H*-phosphonate. In the next step, the dry solid support was washed with a solution of *N*,*O*-bis(trimethylsilyl)acetamide (0.4 ml, 1.64 mmol), imidazole (150 mg, 2 mmol), CCl_4_ (0.75 ml), acetonitrile (0.75 ml) and triethylamine (0.1 ml) for 4 h, and subsequently washed with acetonitrile, and dried under argon to obtain an imidazole-activated 5′-phosphate group (38). Finally, the imidazolide was reacted for 1–2 h with a 1.0 M solution of tetrabutylammonium fluoride (TBAF) in tetrahydrofuran (THF) (0.2 ml, 0.2 mmol) and diluted with a dimethylsulfoxide (DMSO; 3 ml) solution of ZnCl_2_ (10 mg, 0.07 mmol). Next, the solid support was washed with ethylenediaminetetraacetic acid (EDTA) (10 mg/ml)/NaHCO_3_ (5 mg/ml) aqueous solution followed by washing with acetonitrile, dried under argon and cleaved from the solid support as described above. After cleavage of FPON1 and FP-hTeloC, an additional step involving nucleophilic substitution by TBAF was performed by dissolving the oligonucleotide in a solution of TBAF in THF and diluting it with ZnCl_2_ in DMSO as above. After overnight stirring, the EDTA aqueous solution (see above) was added to the reaction mixture.

FPP analogues were synthesized by reacting a lyophilized 5′-phosphate oligonucleotide with fluorophosphate imidazolide lithium salt, which was synthesized as described previously (36). The 5′-phosphorylated oligonucleotide was moisturized with water (5–10 μl), followed by addition of DMSO (0.2 ml), fluorophosphate imidazolide lithium salt (20–30 equivalents), and MgCl_2_ (20–30 equivalents). The solution was stirred for 6–24 h. Reaction progress was monitored by reversed-phase high-performance liquid chromatography (RP-HPLC) and mass spectrometry with electrospray ionization. After adequate time, the reaction was diluted with an aqueous mixture of EDTA (10 mg/ml).

Unmodified, FP and FPP oligodeoxyribonucleotides were purified by semi-preparative RP-HPLC using a Discovery RP Amide C-16 HPLC column (25 × 2.12 cm, 5 μm, flow rate 5.0 ml/min) or Vision-HT RP C-18 column (25 × 2 cm, 10 μm, flow rate 5.0 ml/min) with detection at 254 nm using a linear gradient of acetonitrile in 0.05 M ammonium acetate buffer (pH 5.9). The identity of all products was confirmed by high-resolution mass spectrometry (HRMS), whereas their homogeneity and purity were determined by RP-HPLC and ^19^F NMR spectroscopy.

### Oligonucleotide sample preparation for NMR and UV-VIS studies

Stock solutions were prepared by dissolving oligonucleotides in 100–200 μl of ultrapure water at ∼2–10 mM. The exact concentrations of oligonucleotides (FPON1, FPPON1, FPON3, FPPON3, FP-hTeloC, ON1, ON3-ON5, ON9, ON11, ON20-ON25, hTeloC and PON11) were determined spectrophotometrically at 260 nm using theoretical extinction coefficients in phosphate buffer (pH 7.0). The appropriate oligonucleotide was then diluted in a selected buffer based on the end application: duplex formation studies (20 mM sodium phosphate, 0.15 M NaCl, 0.2 mM disodium EDTA, pH 7.0, 10% D_2_O); *i*-motif studies (50 mM sodium citrate, pH 4.2, 10% D_2_O); G-quadruplex studies (10 mM KH_2_PO_4_, 70 mM KCl, 0.2 mM disodium EDTA, pH 7.0, 10% D_2_O).

### ^1^H NMR spectroscopy studies

^1^H NMR spectra were recorded on a Bruker Avance III HD 500 MHz spectrometer equipped with a 5-mm PABBO BB/19F-1H/D Z-GRD probe at a frequency of 500.24 MHz. If needed, water suppression was achieved using a 3-9-19 pulse sequence with gradients (WATERGATE) (39,40). The ^1^H NMR chemical shifts were compared to sodium 3-(trimethylsilyl)-2,2′,3,3′-tetradeuteropropionate (TSP) (*δ*_H_ 0 ppm) as an internal standard.

### ^19^F NMR spectroscopy studies

^19^F NMR spectra were recorded as above at a frequency of 470.67 MHz in 5-mm NMR samples. Typical experimental parameters were as follows: ^19^F excitation pulse, 15.0 μs; acquisition time, 1.19 s; prescan delay, 6.0 μs; relaxation delay, 1.0 s; usual number of scans, 512–4096 or up to 8000 in the case of G-quadruplex studies. The ^19^F NMR chemical shifts were compared to either 10 mM NaF in D_2_O (δ_F_ = −121.5 ppm) or 0.5 mM 2,2,2-trifluoroethanol (δ_F_ = −76.0 ppm) used as external standards.

#### Duplex formation and mismatch identification

Fluorophosphorylated oligonucleotides FPON3 and FPPON3 in 10 mM aqueous stock solutions were diluted in phosphate buffer (20 mM sodium phosphate, 0.15 M NaCl, 0.2 mM EDTA, pH 7.0, 10% D_2_O) to a final concentration of 0.1 and 0.2 mM, respectively. Titrations were performed by adding aliquots of complementary oligonucleotide from the stock solutions. Before acquiring each NMR spectrum, the sample was incubated at 90°C for 3 min and then cooled to room temperature on ice. NMR measurements were carried out at 23°C.

#### *i*-Motif studies

Fluorophosphorylated oligonucleotides FPON1, FPPON1 and FP-hTeloC were prepared as 5–8 mM stock solutions in ultra-pure water and diluted to a final concentration of 0.1–0.5 mM in citrate buffer (50 mM sodium citrate, pH 4.2, 10% D_2_O) or cacodylate buffer (10 mM sodium cacodylate, 100 mM NaCl, pH 5.5, 10% D_2_O). If needed, pH was determined in a 1.5-ml tube by a calibrated pH-meter equipped with a glass electrode specifically adapted for measurements in a small volume. The pH was adjusted by dropwise additions of 0.1 M HCl or 0.1 M NaOH/KOH. In the case of temperature-dependent studies, the samples were incubated inside the magnet for 10 min before starting data acquisition. For pH-dependent studies the temperature was set to 25°C. Mitoxantrone dihydrochloride and cationic porphyrin TMPyP4 tetratosylate were used as titrants in the studies with FP-hTeloC and FPON1, respectively; they were purchased from Sigma-Aldrich as lyophilized powders and dissolved in water at a concentration of 10 mM. Adenosine 5′-fluoromonophospate (AMPF) was used as negative control in interaction studies between *i*-motifs and ligands; it was synthesized as previously described (36) and dissolved in ultra-pure water at a concentration of 10 mM. To calculate the dissociation constant (*K*_d_), a one-site-specific binding model was fitted to the data using GraphPad Prism 8.3 software (Equation 1):

(1) $$\begin{equation*}\Delta {\delta _{ppm}} = \frac{{\Delta \delta _{ppm}^{\max }*{c_M}}}{{{K_d} + {c_M}}}\end{equation*}$$

where Δ*δ*_ppm_ represents the observed chemical shift change relative to free oligonucleotide, c_M_ is the concentration of mitoxantrone (in μM), Δ*δ*_ppm_^max^ is the chemical shift change value extrapolated to saturating concentrations of mitoxantrone and *K*_d_ is the equilibrium dissociation constant.

#### G-quadruplex formation and protein interaction studies

The fluorophosphorylated oligonucleotide FPPON11 in 2.50 mM aqueous stock solution was diluted to a final concentration of 70 μM in phosphate buffer (10 mM KH_2_PO_4_, 70 mM KCl, 0.2 mM disodium EDTA, pH 7.0, 10% D_2_O). Prior to NMR measurements, the sample was heated to 90°C and cooled on the bench to room temperature (21–23°C). Thrombin from bovine plasma served as a titrant in nucleic acid–protein interaction studies; it was purchased from Sigma-Aldrich (T4648-1KU) as a lyophilized powder and dissolved in water to a concentration of 10 mg/ml (137 μM).

### UV-VIS measurements

For duplex formation studies, oligonucleotides FPON3, FPPON3, ON3-ON5, ON9 and ON20-ON25 were diluted with sodium phosphate buffer to a final concentration of 2 μM (20 mM sodium phosphate, 0.15 M NaCl, 0.2 mM EDTA, pH 7.0, 10% D_2_O) and mixed together in a 1:1 ratio (to a final concentration of 2 μM each). For G-quadruplex formation studies, oligonucleotides FPPON11, PON11 and ON11 were diluted in potassium phosphate buffer to a final concentration of 4 μM (10 mM KH_2_PO_4_, 70 mM KCl, 0.2 mM disodium EDTA, pH 7.0, 10% D_2_O). For *i*-motif formation studies, oligonucleotides ON1, FPON1, FPPON1 and hTeloC were diluted in sodium citrate buffer to a final concentration of 12 μM (50 mM sodium citrate, 10% D_2_O, pH 4.2 or 5.5). The measurements were performed in 1 × 1 cm cuvettes with a sample volume of 2 ml. Melting temperature determination was performed on a Thermo Scientific Evolution 350 UV-VIS spectrometer, equipped with an 8 Cell Peltier Thermostatted System and Temperature Probe, at a temperature ranging from 10°C to 90°C (ramp rate 1.0°C/min). Duplex formation was monitored at 260 nm, whereas G-quadruplex and *i*-motif formation were monitored at 295 and 260 nm. All experiments were performed in triplicate.

### Melting temperature and transition pH determination

^19^F NMR and UV-VIS data were analyzed by normalizing the integrated ^19^F NMR signal or absorbance in GraphPad Prism 8.3 software: 0% was defined as the smallest mean, whereas 100% was defined as the largest mean value in each dataset. To determine the melting temperatures (*T*_m_) or transitional pH (pH_T_) the following equation was fitted:

(2) $$\begin{equation*}A\,{\rm or}\,I = {\rm Bottom} + \frac{{({\rm Top - Bottom})}}{{1 + {e^{\frac{{V50 - X}}{{{\rm slope}}}}}}}\end{equation*}$$

where normalized absorbance (A) or normalized ^19^F NMR signal integration (I) vary from BOTTOM to TOP, and V_50_ is the value of *T*_m_ or pH_T_.

## RESULTS

### Synthesis of 5′-fluorophosphate oligonucleotides

**Figure 2.**
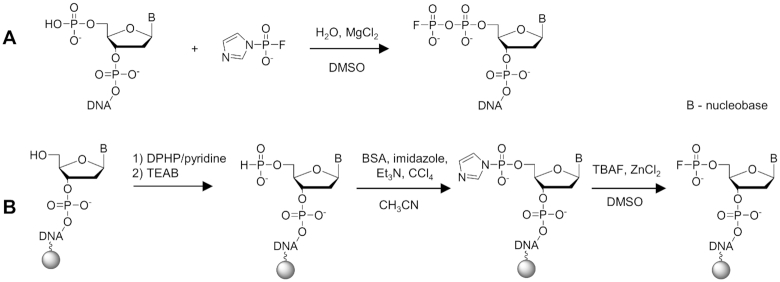
Synthesis of 5′-fluoro(mono)- and 5′-fluoro(di)phosphate-labeled oligonucleotides (FPONs and FPPONs). (**A**) FPPONs obtained by reacting solid-phase synthesized oligonucleotide 5′-phosphates with an activated fluorophosphate subunit (FpIm). (**B**) FPONs obtained by activation on a solid support and reaction with TBAF. DPHP: Diphenyl *H*-phosphonate; BSA: *N*,*O*-Bis(trimethylsilyl)acetamide.

To explore the use of FPPONs and FPONs as molecular probes for ^19^F NMR studies, we synthesized a set of 18 oligonucleotide probes containing these moieties (Table 1). To obtain FPPONs, a previously developed approach based on an imidazole-activated fluorophosphate subunit (FpIm) as an electrophilic fluorophosphorylating agent was adapted (Figure 2A) (36). FPONs were generated by reacting imidazole-activated oligonucleotide 5′-monophosphates with TBAF (Figure 2B).

**Table 1.** Summary of fluoro(di)phosphorylated oligonucleotide probes

Probe name	Length (nt)	5′-terminal group^[a]^	Sequence (5′→3′)	HPLC conversion	Isolated yield^[b]^	Molecular formula of [M-nH]^n−^ ion	Calc. (m/z)	Actual (m/z)
FPON1^[c]^	6	Fp	TCC CCC	80%	20%	C_55_H_72_FN_17_O_37_P_6_^2−^	883.63478	883.63531
FPPON1^[c]^	6	Fpp	TCC CCC	61%	35%	C_55_H_73_FN_17_O_40_P_7_^2−^	923.61795	923.61885
FPPON2	7	Fpp	GTC AAT G	86%	45%	C_69_H_86_FN_27_O_45_P_8_^2−^	1139.65835	1139.65965
FPON3^[c]^	10	Fp	AGA CAT TGA C	60%	15%	C_98_H_120_FN_40_O_58_P_10_^3−^	1037.83489	1037.83648
FPPON3^[c]^	10	Fpp	AGA CAT TGA C	86%	30%	C_98_H_121_FN_40_O_61_P_11_^3−^	1064.49033	1064.49194
FPPON4	10	Fpp	TGA CAT TGA C	93%	49%	C_98_H_122_FN_37_O_63_P_11_^3−^	1061.48648	1061.48788
FPPON5	10	Fpp	GTC AAT GTC C	82%	41%	C_97_H_122_FN_35_O_64_P_11_^3−^	1053.48273	1053.48426
FPPON6	10	Fpp	GGA CAT TGA C	99%*	48%	C_98_H_121_FN_40_O_62_P_11_^3−^	1069.82197	1069.82293
FPPON7	10	Fpp	ATC AAT GTC G	85%	51%	C_98_H_122_FN_37_O_63_P_11_^3−^	1061.48648	1061.48737
FPPON8	10	Fpp	CGA CAT TGA T	99%*	58%	C_98_H_122_FN_37_O_63_P_11_^3−^	1061.48648	1061.48751
FPPON9	10	Fpp	GTC AAT GTC T	88%	48%	C_98_H_123_FN_34_O_65_P_11_^3−^	1058.48262	1058.48364
FPPON10	13	Fpp	GTC AAT GTC AGC G	80%	41%	C_127_H_158_FN_50_O_81_P_14_^3−^	1377.20362	1377.20421
FPPON11**^[c]^**	15	Fpp	GGT TGG TGT GGT TGG	82%	42%	C_150_H_184_FN_57_O_99_P_16_^4−^	1220.42309	1220.42932
FPPON12	16	Fpp	GGA TAC TTT TGT ATC C	78%	35%	C_157_H_194_FN_62_O_98_P_17_^4−^	1254.43380	1254.43400
FPPON13	16	Fpp	GGA TAT TTT TAT ATC C	97%*	19%	C_158_H_198_FN_52_O_103_P_17_^4−^	1254.18499	1254.18523
FPPON14	16	Fpp	GTC AAT GTC AGC GAT A	85%	30%	C_157_H_195_FN_62_O_98_P_17_^3−^	1687.25737	1687.25876
FPPON15	25	Fpp	CCT GGG GGA GTA TTG CGG AGG AAG G	95%*	20%	C_247_H_301_FN_107_O_152_P_26_^5−^	1604.24604	1604.24765
FP-hTeloC^[b]^	24	Fp	TAA CCC TAA CCC TAA CCC TAA CCC	76%	24%	C_228_H_289_FN_84_O_140_P_24_^4−^	1801.30192	1801.29517

^[a]^ Fpp, fluorodiphosphate; Fp, fluorophosphate; ^[b]^ For FP overall yield including solid phase synthesis is reported; For FPP the yield for the final step (reaction with FpIm) is reported; ^[c]^ Oligonucleotide used as ^19^F molecular probe in monitoring of *i*-motif structure changes; ^[c]^ Oligonucleotide used as ^19^F molecular probe in hybridization studies; ^[d]^ Oligonucleotide used as ^19^F molecular probe in monitoring of G-quadruplex structure changes and nucleic acid–protein interactions; *approximate value due to HPLC signal overlap.

Oligonucleotides are listed by length.

FPPONs were synthesized in solution starting from oligonucleotide 5′-monophosphates prepared by conventional phosphoramidite solid-phase synthesis (41). After cleavage and deprotection, the oligonucleotide 5′-monophosphate methylamine salt was converted into 5′-fluorodiphosphate in a one-step reaction, with an excess of activated FpIm (30 equivalents) and MgCl_2_ (30 equivalents) as a reaction mediator (Figure 2A). Using this approach, 15 oligonucleotides of varying length (from 6 to 25 nt) (Table 1) were synthesized. The set included ten oligonucleotides of various lengths designed for duplex formation studies (FPPON2–FPPON10 and FPPON14), a 15-nt G-quadruplex-forming sequence (FPPON11), an *i*-motif-forming sequence (FPPON1) and three longer sequences of 16 nt (FPPON12 and FPPON13) or 25 nt (FPPON15). The isolated yields for fluorophosphorylation varied from 19 to 58%, but usually reached 30%. The final products were purified by RP-HPLC.

To obtain FPONs on solid support, 5′-deprotected oligonucleotides synthesized by the conventional phosphoramidite method were modified while still attached to the support. The 5′-OH group of the oligonucleotide was converted to *H*-phosphonate monoester by treatment with diphenyl *H*-phosphonate in pyridine, followed by hydrolysis with triethylammonium bicarbonate buffer. The resulting 5′-*H*-phosphonate was oxidized to *P*-imidazolide by a reaction with carbon tetrachloride in the presence of imidazole and *N*,*O*-bis(trimethylsilyl)acetamide (BSA) (38). Finally, a nucleophilic substitution of imidazole with fluoride (using TBAF as a source) was carried out in the presence of excess ZnCl_2_, followed by cleavage from the support with a mixture of 30% ammonia and 40% aqueous methylamine (AMA) to yield the desired FPON (Figure 2B) (36). Notably, no side-reactions associated with the P-F bond cleavage were observed under these conditions. The approach was used to synthesize a molecular probe designed for DNA duplex formation monitoring (FPON3) with a 15% yield, and two cytosine-rich sequences, FPON1 and FP-hTeloC, designed to study *i*-motif structure changes and stabilization, with yields of 20 and 24%, respectively (Table 1). All synthesized oligonucleotides were purified by RP-HPLC and characterized by HRMS as well as ^19^F NMR spectroscopy (Supplementary Data).

To verify whether the fluoro(di)phosphate oligonucleotide analogues were suitable for ^19^F NMR studies, we evaluated them as molecular probes to monitor different phenomena including DNA duplex formation, G-quadruplex formation and interaction with proteins, as well as *i*-motif assembly and interaction with small molecules (Supplementary Table S2).

### Duplex formation and mismatch identification

**Figure 3.**
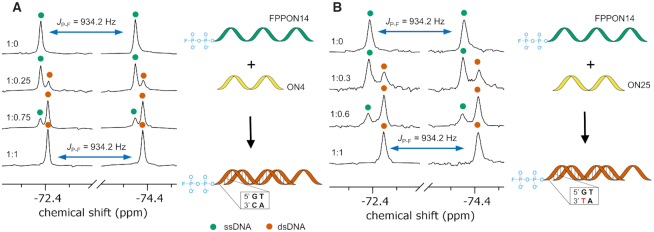
^19^F NMR (470.67 MHz) spectra of fluorine-labeled oligonucleotides in single-stranded (ss) and double-stranded (ds) form. (**A**) ^19^F NMR duplex formation monitoring between FPPON14 and ON4. (**B**) ^19^F NMR duplex formation monitoring between FPPON14 and ON25; Green dots indicate ssDNA, orange dots indicate dsDNA. All spectra were recorded at 23°C in 20 mM sodium phosphate, 0.15 M NaCl, 0.2 mM disodium EDTA, pH 7.0 and 10% D_2_O.

We first explored the suitability of fluorophosphorylated oligonucleotides (FPON3, FPPON3 and FPPON14) to monitor duplex formation and identify 3′-terminal mismatches in a complementary strand by ^19^F NMR. Oligonucleotides used in these experiments differed in sequence, length (10–16 nt) and 5′-terminal tags (either FP or FPP). For initial studies, we chose the 16-nt FPP oligonucleotide FPPON14. The ^19^F NMR spectrum of FPPON14 showed a single doublet at −73.38 ppm and *J_P-F_* = 934.2 Hz (Figure 3A and B, top spectra). We next verified whether it was possible to monitor duplex formation between FPPON14 and either the fully complementary 10-nt ON4 or the single-mismatched 10-nt ON25 (Supplementary Table S1). The addition of 0.25 equivalents of ON4 to FPPON14 resulted in the appearance of a new signal at *δ*_F_ −73.40 ppm (*J_P-F_* = 934.2 Hz), whose intensity increased with further addition of complementary oligonucleotide (up to one equivalent), while the original signal of the ssDNA disappeared completely (Figure 3A). In an analogous experiment, the addition of one equivalent of ON25 to FPPON14 resulted in the appearance of a new signal at *δ*_F_ −73.42 ppm and disappearance of the ssDNA signal (Figure 3B). Duplex formation under the above conditions was independently confirmed using UV-VIS spectroscopy (Supplementary Figure S1). By comparing the changes in chemical shifts between ssDNA and dsDNA, a chemical shift of 0.02 ppm was detected between duplexes with ON4 and ON25. This observation suggested that our probes could be used for 5′/3′ terminal mismatch identification by ^19^F NMR.

To further explore this possibility, we synthesized a 10-nt model sequence, ON3, carrying either a 5′-monofluorophosphate (FPON3) or 5′-difluorophosphate (FPPON3) and a library of oligonucleotides (ON5, ON9 and ON20–ON25; Supplementary Table S1) that were either fully complementary to the ON3 sequence, or complementary except for a single mismatch at the last (X) or second to last (Y) position from their 3′ ends. To assess the influence of FPP and FP moieties on DNA duplex stability, we first studied the stability of these fully or partially complementary duplexes with respect to an identical but unmodified (5′-OH) ON3 sequence (Supplementary Figures S2–4). *T*_m_ was determined via UV-VIS spectroscopy by fitting a theoretical model to the determined temperature-dependent sigmoidal curves (Table 2) (42). The fully complementary duplexes of FPON3, FPPON3 and ON3 with ON9 showed similar stability, with *T*_m_ values of 40.8 ± 0.1°C, 41.0 ± 0.1°C and 41.6 ± 0.1°C, respectively (Table 2, line 1). Duplexes with sequences carrying a single mismatch (ON5, ON20–ON21) at the 3′ end (i.e. at the 5′ end of the fluorophosphorylated probe) resulted in *T*_m_ values of 39–41°C, with the 5′-modified duplexes showing lesser stability (0.6–1.5°C lower *T*_m_) than the unmodified counterparts (Table 2, lines 2–4). The sequences with single mismatch (ON22–ON24) at the second to last position from the 3′ end resulted in *T*_m_ values of 29–31°C (Table 2, lines 5–7), with the 5′-terminal modifications having an additional slightly destabilizing influence. Overall, these results revealed that the complementarity of the last base pair had little influence on duplex stability; therefore, these oligos are good model sequences to study mismatches and other modifications that do not affect duplex stability significantly.

**Table 2.** Summary of melting temperatures determined for fully and partially complementary duplexes containing the AGA CAT TGA C sequence tagged with FP, FPP or OH at the 5′ terminus

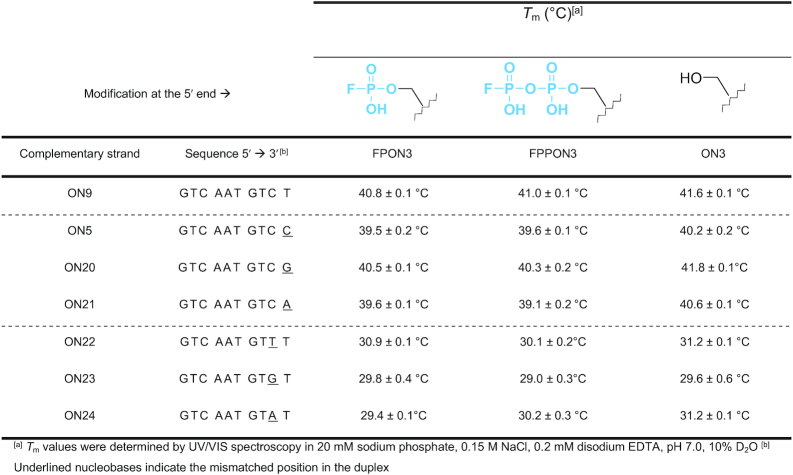

**Figure 4.**
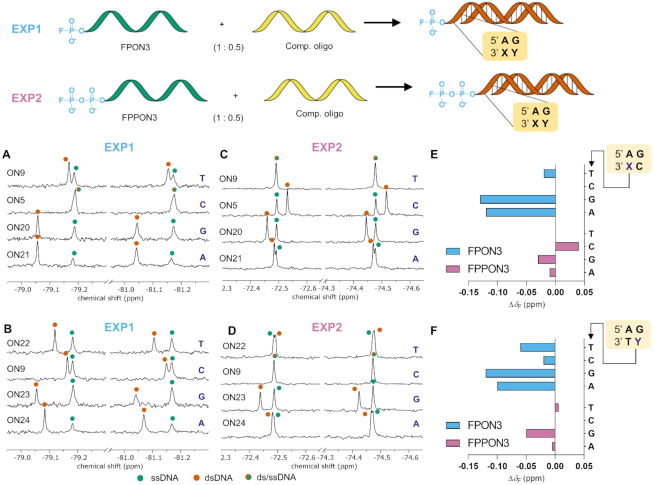
DNA hybridization monitoring and mismatch identification by ^19^F NMR spectroscopy. FPON3 (EXP1) and FPPON3 (EXP2) sequences were used to monitor dsDNA formation and mismatch identification. Fluorophosphorylated ONs were mixed with 0.5 equivalents of different complementary or partially complementary (single mismatch at X or Y position from the 3′ end) oligonucleotides (ON5, ON9, ON20–ON24) and ^19^F NMR spectra were recorded. Color-coding was used to indicate the resonances for ssDNA (green dots), dsDNA (orange dots) and overlapping dsDNA and ssDNA signals (green-orange dots). Downfield or upfield shifted components of each doublet were analyzed to calculate Δ*δ*_F_ differences between ssDNA and dsDNA. Measurements were performed in 20 mM sodium phosphate, 0.15 M NaCl, 0.2 mM disodium EDTA, pH 7.0, 10% D_2_O buffer at 23°C.

We subsequently tested whether it was possible to detect duplex formation for these sequences and identify 3′-terminal sequence mismatches in the complementary strand based on their unique ^19^F NMR signatures (EXP1 and EXP2; Figure 4). Mixtures of fluorophosphorylated (200 μM) and non-modified oligonucleotides (added stepwise from 0 to 200 μM) were prepared and ^19^F NMR spectra were recorded at 23°C (Supplementary Figures S5–18). Interestingly, for almost all tested mismatches, a distinct *δ*_F_ chemical shift corresponding to the formed duplex was observed (Figure 4A–D); this value differed from both the *δ*_F_ value of the ssDNA and that of the perfect duplex. The largest changes in chemical shifts between ssDNA (green dot) and dsDNA (orange dot) were observed when a fluoromonophosphorylated oligonucleotide (FPON3) was used and if purines were mismatched against purines. Formation of a duplex between FPON3 and the complementary strand ON9 (Figure 4A and B) resulted in a new signal at 0.02 ppm downfield from ssFPON3. The duplex with ON5 carrying an A/C mismatch at the X position was virtually indistinguishable from ssFPON3; whereas new downfield shifts of 0.13 ppm and 0.12 ppm were observed if A was mismatched against G or A in the duplex with ON20 and ON21, respectively (Figure 4A). A similar result was observed for duplex formation with partially complementary sequences, in which mismatch was located at the Y position. ^19^F NMR spectra of FPON3 in the presence of 0.5 equivalents of ON22, ON23 or ON24 revealed the appearance of new signals corresponding to dsDNA, with *δ*_F_ values shifted downfield by 0.06, 0.12 and 0.10 ppm, respectively (Figure 4B). Next, similar experiments were performed with FPPON3 to reveal a notably different set of ^19^F NMR signatures. As shown in Figure 4C and D, duplex formation of FPPON3 with the fully complementary oligonucleotide ON9 (A ↔T) resulted in no changes to the ^19^F NMR spectrum. In contrast, duplexes formed with partially complementary ON5 (A ↔ C), ON20 (A ↔ G) and ON21 (A ↔ A) resulted in the appearance of signals shifted by 0.04, −0.03 and −0.01 ppm, respectively (Figure 4C). For duplexes in which mismatch was located at the Y position, smaller chemical shift changes were generally observed upon duplex formation. The largest Δ*δ*_ppm_ value was observed for the duplex with ON23 (G ↔ G; Δ*δ*_F_ = −0.05 ppm) and the smallest for duplexes with ON22 (G ↔ T; Δ*δ*_F_ = 0.006 ppm) and ON24 (G ↔ A, Δ*δ*_F_ = −0.005 ppm) (Figure 4D). Although the observed chemical shift differences were smaller for FPPON3 than FPON3, the obtained ^19^F NMR datasets were complementary to each other and their combination revealed unambiguous characteristics of each duplex (Figure 4E and F).

### G-quadruplex formation and protein–nucleic acid interaction monitoring

Guanine-rich sequences tend to form non-canonical DNA structures called G-quadruplexes (43). These secondary DNA structures are stabilized by a network of Hoogsteen hydrogen bonds between four guanine bases and a centrally located monovalent cation (preferably K^+^). G-quadruplexes have been identified as important elements in gene expression regulation and genome instability (44–47). Small compounds interacting with and stabilizing G-quadruplexes may serve as gene expression modulators and drugs in anti-cancer therapy (48). Hence, developing methods to study these DNA structures is of broad interest.

**Figure 5.**
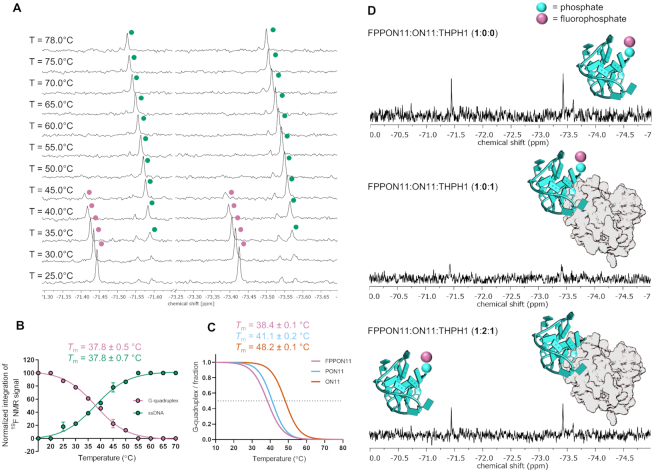
^19^F NMR studies on a 5′-fluorodiphosphorylated TBA sequence forming a G-quadruplex structure (FPPON11). (**A**) ^19^F NMR spectra of FPPON11 as a function of temperature. (**B**) Melting temperature determination by analysis of integrated ^19^F NMR signals. (**C**) UV/VIS melting profile of FPPON11, ON11 and PON11 (A_295nm_). (**D**) Protein–nucleic acid interaction and ligand displacement experiment. Top: ^19^F NMR spectrum of FPPON11; middle: ^19^F NMR spectrum of FPPON11 in complex with thrombin; bottom: ^19^F NMR spectrum recorded after addition of two equivalents of TBA sequence (ON11). All signals were referenced to CF_3_CH_2_OH (−76.00 ppm). The protein structure was adapted from PDB entry: 4DII (73), the scale has not been preserved.

To verify the usefulness of FP labeling for studying G-quadruplexes by ^19^F NMR, we introduced the fluorodiphosphate moiety at the 5′ end of the thrombin-binding aptamer (TBA) sequence (ON11). TBA is an artificial 15-nt deoxyribooligonucleotide that forms a potassium-stabilized G-quadruplex consisting of two G-tetrads and a TGT loop (49). The aptamer, discovered in 1992 by systematic evolution of ligands by exponential enrichment, interacts with thrombin with nanomolar *K*_d_ and inhibits fibrin-clot formation (50,51). Using ^1^H NMR, we confirmed that FPPON11 formed a G-quadruplex structure at 25°C in the presence of K^+^, similar to the unmodified TBA sequence (52). The imino resonances typical of G-quadruplexes (*δ*_H_ 11.8–12.5 ppm) were clearly observed at room temperature, but not at 78°C (Supplementary Figure S19). Next, we tested whether temperature-induced unfolding of this TBA G-quadruplex could be observed by ^19^F chemical shift changes in temperature-dependent spectra of FPPON11. ^19^F NMR spectra of FPPON11 at 25°C showed a doublet at −72.43 ppm (*J*_P-F_ = 933 Hz). A stepwise temperature increase (up to 78°C) resulted in the appearance of a new signal (−72.51 ppm, *J*_P-F_ = 929 Hz, Δ*δ*_F_ = 0.016 ppm at 40°C), while the original signal decreased in intensity until it disappeared completely at 55°C (Figure 5A). The ^19^F NMR signals at different temperatures were integrated, normalized, and the relative intensities were plotted as a function of temperature, followed by fitting a theoretical curve to determine *T*_m_. The *T*_m_ value was 37.8 ± 0.5°C if G-quadruplex signal was used for the analysis and 37.8 ± 0.7°C if ssDNA signal was analyzed (Figure 5B). The results were independently verified under the same conditions by UV-VIS temperature-dependent measurements. *T*_m_ values for unmodified TBA (ON11), 5′-phosphate TBA (PON11) and FPPON11 were determined (Figure 5C). The 5′-phoshpate (P) group lowered the melting temperature by 7°C (T*_m_* = 41.1 ± 0.1°C) compared to unmodified ON11 (*T_m_* = 48.2 ± 0.1°C). Further destabilization by 2.7°C was observed for FPPON11, resulting in a *T*_m_ of 38.4 ± 0.1°C (Table 3).

**Table 3.** Comparison of melting temperatures of thrombin-binding aptamers containing different chemical groups at the 5′ end

		5′ GGT TGG TGT GGT TGG 3′	
Oligo.	5′-terminal group	*T*_m_ [°C]	method
ON11	OH	48.2 ± 0.1	UV/VIS
PON11	P	41.1 ± 0.2	UV/VIS
FPPON11	FPP	38.4 ± 0.1	UV/VIS
		37.8 ± 0.5^a^	^19^F NMR
		37.8 ± 0.7^b^	^19^F NMR

^a^based on G-quadruplex ^19^F NMR signal, ^b^ based on ssDNA ^19^F NMR signal.

After confirming that the fluorophosphate moiety enabled monitoring of G-quadruplex formation by ^19^F NMR, we next tested whether a similar approach could be used to monitor the TBA–thrombin interaction. To this end, thrombin (THPH1) was titrated into a solution of FPON11 and the corresponding ^19^F NMR spectrum was recorded after each addition (Figure 5D). A significant widening of the ^19^F NMR signal was observed following THPH1 addition, in line with a significantly increased molecular weight and consequently slower tumbling of oligo in the complex with THPH1 (53). The signal was restored after addition of a competitive TBA binder (ON11), in accordance with displacement of the fluorinated probe from the complex (Figure 5D, bottom and Supplementary Figure S20).

### *i*-Motif formation monitoring

*i*-Motifs are another class of non-canonical secondary nucleic acid structures, which are formed by cytosine-rich sequences. *i*-Motifs were initially found to be stable only under acidic conditions and at low temperatures; hence, the potential biological significance of these structures has not been fully appreciated. In recent years, the initial views on *i*-motifs have been challenged as it has been revealed that these structures can be stable even at neutral pH (54,55) and are additionally stabilized under molecular crowding conditions (56). Finally, *i*-motif structures have been detected in the nuclei of human cells, suggesting that they may play regulatory roles in the genome, which has sparked renewed interest (57). Interactions between ligands and *i*-motifs have been investigated with the aim of identifying molecules capable of modulating *i*-motif stability (58–60).

**Figure 6.**
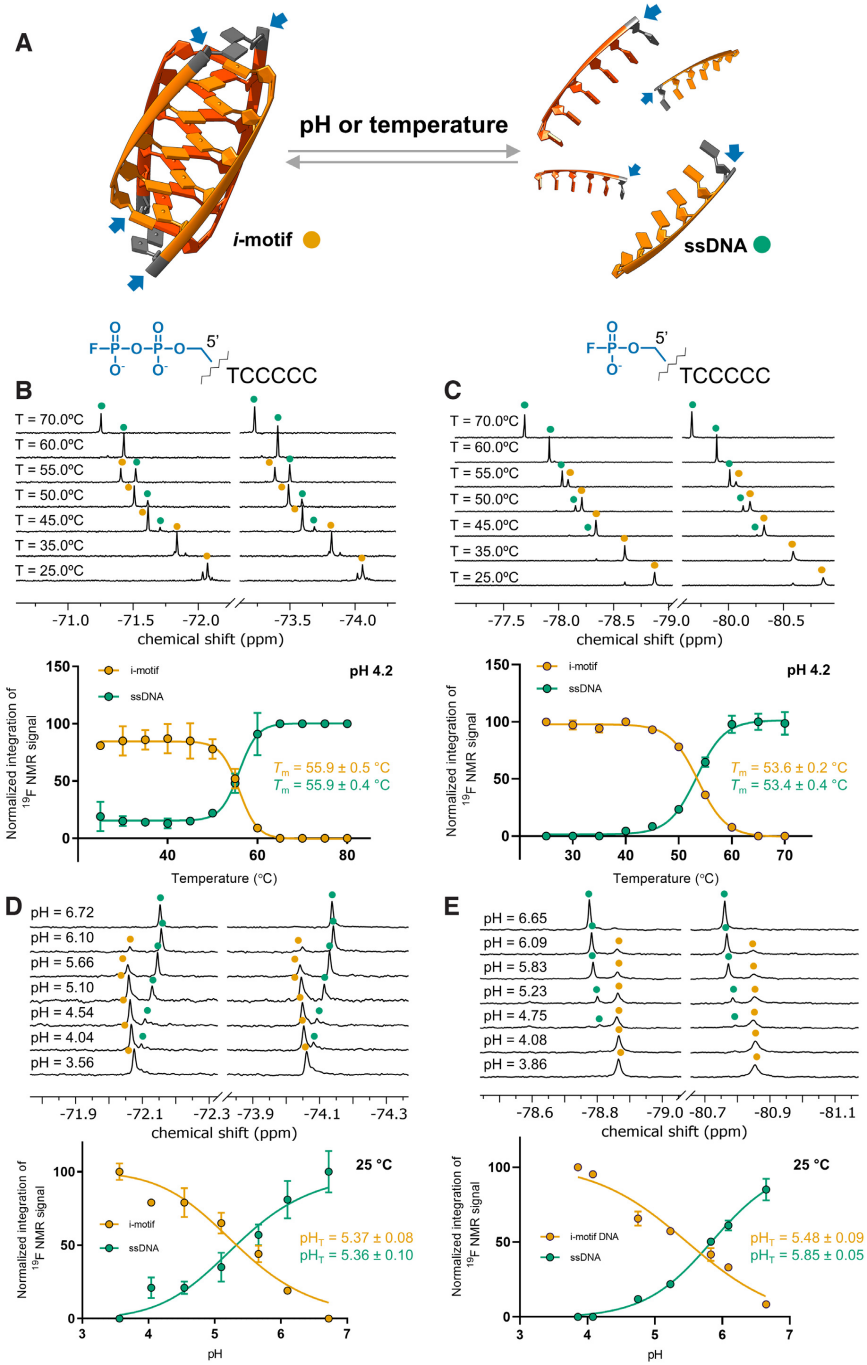
^19^F NMR studies of FPP and FP *i*-motif sequences (FPPO1 and FPON1, respectively). (**A**) TC_5_ sequence as building block of the *i*-motif structure (structure taken from PDB entry: 225D) and as ssDNA; thymines are in gray, cytidines are in orange/red orange, blue arrows indicate positions of FP or FPP ^19^F NMR tags. (**B** and **C**) ^19^F NMR spectra at different temperatures: 100 μM FPPON1 or 500 μM FPON1, 50 mM sodium citrate buffer, 10% D_2_O, pH 4.20. (**D** and **E**) ^19^F NMR spectra at different pH, conditions: 100 μM FPPON1 or 500 μM FPON1, 50 mM sodium citrate buffer, 10% D_2_O, 25°C.

Here, we aimed to verify whether fluorophosphate-labeled oligonucleotides could be used to monitor pH- and temperature-dependent *i*-motif (un)folding. To this end, we first generated a TCCCCC sequence labeled with FP (FPON1) or FPP (FPPON1) moieties and their unmodified counterpart (ON1). This short DNA sequence consisting of a single thymine and five cytidines forms a tetrameric *i*-motif and is a simple, well-known model molecule for studying *i*-motif stability, as well as the effects of different chemical modifications on *i*-motif structure (61). Using ^1^H NMR, we confirmed that all three variants of the ON1 sequence formed *i*-motifs at 10°C in an aqueous buffer at pH 4.2 (10% D_2_O). As expected, the presence of imino signals (*δ*_H_ 15–16 ppm) was clearly visible in the ^1^H NMR spectra, which was consistent with the presence of protonated C·CH^+^ base pairs characteristic of the *i*-motif structure (Supplementary Figure S21) (62). Increasing the temperature above 50°C resulted in the disappearance of imino resonances. ^19^F NMR spectra of FPON1 and FPPON1 recorded under conditions expected to favor *i*-motif formation (pH 4.2, 25°C) revealed presence of a doublet for each form (δ_F_ −79.86 ppm and −73.06 ppm, respectively; Supplementary Figure S22). Both FPON1 and FPPON1 were used in a series of temperature- and pH-dependent ^19^F NMR experiments, in which we attempted to monitor stepwise *i*-motif unfolding (Figure 6A). The temperature-dependent ^19^F NMR spectra of FPPON1 and FPON1 were recorded in the range of 25 to 70°C (step of 5°C) at pH 4.20 (Supplementary Figures S23 and 24). For FPPON1 at 25°C, a single doublet (*δ*_F_ −73.06 ppm, *J*_P-F_ = 934.7 Hz) was observed, which disappeared with increasing temperature along with the appearance of a new doublet (*δ*_F_ −72.24 ppm at 70°C, *J*_P-F_ = 929.8 Hz) (Figure 6B). At intermediate temperatures (40–60°C), varying proportions of both signals were clearly observed. Similar changes in ^19^F NMR spectra were observed for FPON1. A signal visible at 25°C (*δ*_F_ −79.86 ppm, *J*_P-F_ = 936.4 Hz) disappeared at 70°C, while a new doublet appeared (*δ*_F_ −78.68 ppm, *J*_P-F_ = 930.0 Hz) (Figure 6C). The influence of pH changes (in the range of ∼3.5–7) on ^19^F NMR spectra at a constant temperature (25°C) was also studied. At low pH (3.56), one doublet was observed for FPPON1 (*δ*_F_ −73.07 ppm, *J*_P-F_ = 934.6 Hz); whereas increasing the pH resulted in the appearance of a new doublet shifted upfield (*δ*_F_ = −73.15 ppm, *J*_P-F_ = 933.9 Hz, Δ*δ*_F_ = 0.08 ppm, pH 6.72; Figure 6D). Similarly, for FPON1, one major signal was observed at pH 3.86 (*δ*_F_ −79.86 ppm, *J*_P-F_ = 935.9 Hz), but it disappeared with increasing pH and a new downfield-shifted doublet emerged (*δ*_F_ −79.77 ppm, *J*_P-F_ = 934.2 Hz, Δ*δ*_F_ −0.09 ppm, pH 6.65; Figure 6E). In both pH- and temperature-dependent experiments, the appearance of new spectral signals suggested unfolding of the *i*-motif structure and appearance of the single-stranded form. Thus, the intensity of normalized ^19^F NMR signals from each experimental set was plotted as a function of temperature or pH to determine *T*_m_ or pH_T_, respectively (Figure 6). The *T*_m_ and pH_T_ values were calculated by fitting a theoretical curve to the data (Table 4). The *T*_m_ values were also independently determined by UV/VIS measurements at 260 nm (pH 4.2), and were usually in good agreement with those obtained by ^19^F NMR (Supplementary Figure S25 and Table 4). Moreover, the determined values were in good agreement with published data, further confirming that the observed changes in ^19^F NMR spectra were related to *i*-motif (un)folding (Supplementary Table S3).

**Table 4.** Transitional pH (pH_T_) and melting temperature (*T*_m_) values for *i*-motif sequences

			25°C	pH 4.2		pH 5.5	
			pH_T_	*T*_m_		*T*_m_	
Sequence (5′→3′)	Bases	Abbreviation	^19^F NMR ^[a]^	^19^F NMR ^[a]^	UV-VIS	^19^F NMR ^[a]^	UV-VIS
TCCCCC	6	FPON1	5.48 ± 0.09	53.6 ± 0.2°C	57.7 ± 0.3°C	n.d.	n.d
TCCCCC	6	FPPON1	5.37 ± 0.08	55.9 ± 0.5°C	60.7 ± 0.7°C	n.d.	n.d
(TAACCC)_4_	24	FP-hTeloC	6.47 ± 0.07	64.6 ± 1.1°C	54.7 ± 2.4°C	40.9 ± 0.3°C	42.2 ± 0.1°C

Conditions: 50 mM sodium citrate, 10% D_2_O; ^[a] 19^F NMR signal corresponding to *i*-motif structure was analyzed.

Interestingly, while studying FPON1 and FPPON1 by ^19^F NMR, we noted that the spectra recorded at acidic pH contained a varying number of signals, depending on the sample incubation time. Freshly prepared solutions of oligonucleotides usually exhibited more than one signal in the ^19^F NMR spectra (Supplementary Figure S26A); whereas after 48 h of incubation at room temperature (∼20–21°C) only one clearly visible signal was observed (Supplementary Figure S26B). A single signal was observed at high temperatures, confirming the presence of only one fluorine-tagged sequence in solution (Supplementary Figure S26C). Spectra recorded 15 min after lowering the temperature from 70 to 25°C showed at least four clearly visible signals, each with a different intensity. ^19^F NMR spectra produced better signal separation for FPON1 than FPPON1, but in both cases peaks with different chemical shifts could be observed (Supplementary Figure S26D). We believe this observation indicates the presence of different *i*-motif topologies in solution. Such phenomenon was previously observed for a short C_4_T sequence, whereby ^1^H NMR studies revealed an equilibrium between so-called S- and R-forms (63). A more recent study utilized a longer (21-nt) C-rich sequence, which formed major (5′E) and minor (3′E) *i*-motif conformations following a change in pH from 9 to 6 (64). Therefore, we assumed that the initially visible ^19^F NMR signals corresponded to several kinetically favored *i-*motif conformations, which rearranged to the most thermodynamically stable single form over time.

**Figure 7.**
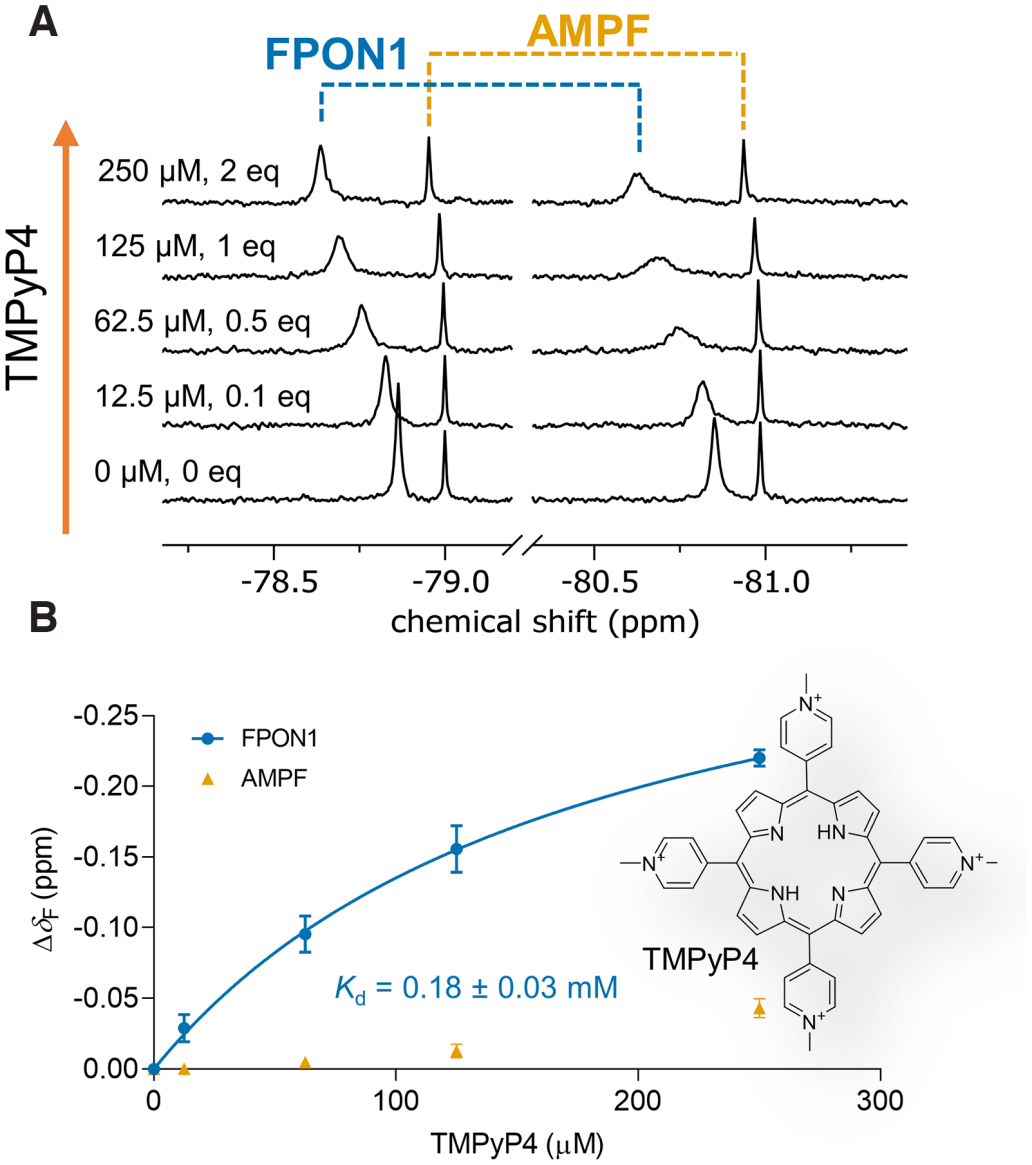
Interaction of *i*-motif-forming sequence, FPON1, with TMPyP4 ligand observed by ^19^F NMR. (**A**) ^19^F NMR spectra recorded during titration of FPON1 with TMPyP4 at pH 4.2 at 500 μM FPON1 (125 μM of *i*-motif structure), 100 μM AMPF, 50 mM sodium citrate buffer pH 4.2, 10% D_2_O, 25°C. (**B**) *δ*_F_ chemical shift changes as a function of TMPyP4 concentration (Δ*δ*_F_ = [*δ*_F_(FPON1 + TMPyP4)–*δ*_F_(FPON1)]); data points represent mean values from duplicate experiments ± S.E.

We next utilized FPON1 as a molecular probe to study *i*-motif interaction with a cationic porphyrin, TMPyP4, by ^19^F NMR (65). When TMPyP4 was titrated into the preformed FPON1 *i-*motif at 0.1–1 equivalents (0–125 μM), we observed signal broadening (Supplementary Figure S27) with simultaneous downfield shifting of the corresponding resonance (Figure 7). To verify that the spectral changes were a result of *i*-motif interaction with TMPyP4, AMPF (100 μM) was used as an internal reference as it was not expected to interact with the ligand (Figure 7A). Addition of a second equivalent of TMPyP4 led to narrowing of the FPON1 ^19^F NMR signal, which was likely caused by saturation of the complex in the presence of excess ligand. ^19^F NMR data were analyzed by plotting chemical shift changes as a function of concentration of the ligand to determine the *K*_d_ value (0.18 ± 0.03 mM; Figure 7B).

**Figure 8.**
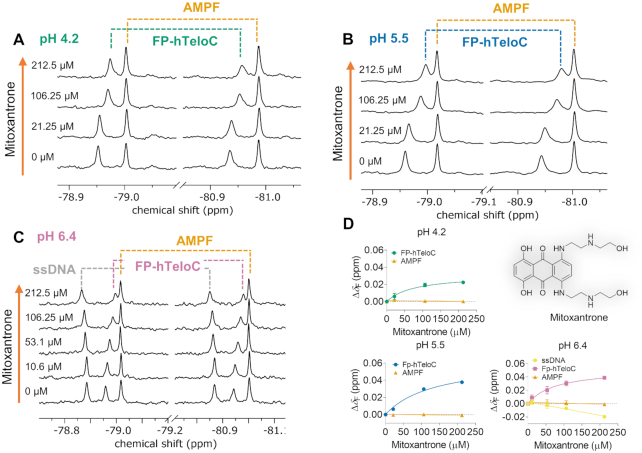
^19^F NMR study of FP-hTeloC with mitoxantrone at different pH. (**A**) Titration of FP-hTeloC with mitoxantrone at pH 4.2. (**B**) Titration of FP-hTeloC with mitoxantrone at pH 5.5. (**C**) Titration of FP-hTeloC with mitoxantrone at pH_T_ 6.4. (**D**) Chemical shift changes as a function of mitoxantrone concentration under different pH conditions (Δ*δ*_F_ = [*δ*_F_(FP-hTeloC+mitoxantrone)–*δ*_F_(FP-hTeloC)]); data points represent mean values from duplicate experiments ± S.E. Conditions: 212.5 μM FP-hTeloC, 80 μM AMPF, 50 mM sodium citrate buffer pH 4.2, 10% D_2_O or 10 mM sodium cacodylate, 100 mM NaCl, 10% D_2_O, pH 6.4. Experiments were performed in duplicate.

Next, we turned our attention to a longer (24-nt) *i*-motif-forming sequence known as hTeloC (5′-TAA CCC TAA CCC TAA CCC TAA CCC-3′). The sequence was found in the human genome within C-rich stretches present in telomers (66). Small molecules that bind to this sequence are potential gene expression modulators. The interaction of hTeloC with multiple ligands has been evaluated by different biophysical methods including CD, fluorescence resonance energy transfer melting screen, UV/VIS spectroscopy and SPR (58,67). To generate an ^19^F NMR probe for the discovery of potential binders, we synthesized an hTeloC sequence labeled with the FP moiety. The initial characterization of FP-hTeloC was performed as described for FPON1 and FPPON1. This confirmed that the sequence formed an *i*-motif structure under acidic conditions (Supplementary Figure S21D) and that pH-dependent unfolding of FP-hTeloC could be observed by ^19^F NMR (Supplementary Figure S28) to determine the pH_T_ value (pH_T_ 6.4; Table 4). We also performed ^19^F NMR-monitored temperature-dependent experiments at two different pH values (pH 4.2 and 5.5; Supplementary Figures S29 and 30) to determine *T*_m_ values of 60 and 40°C, respectively (Table 4). To confirm our findings, we performed UV/VIS spectroscopy temperature-dependent experiments of FP-hTeloC and its unmodified counterpart, hTeloC, at both pH 4.2 and 5.5 (Supplementary Figures S31 and 32). The observed *T*_m_ values for the tested hTeloC sequences were 55°C at pH 4.2 and 43°C at pH 5.5 (Supplementary Table S4), which was in good agreement with published data (Supplementary Table S5), albeit there was a noticeable discrepancy between the values determined for FP-hTeloC by ^19^F NMR and UV/VIS at pH 4.2 (Table 4). We hypothesize that this discrepancy may be a result of either contribution from some minor secondary form that affects the results of UV-VIS measurements (which form was observed by ^19^F NMR at lower pH values, Supplementary Figure S29) or insufficient equilibration times prior to ^19^F NMR measurements. After this preliminary characterization, we investigated the effect of mitoxantrone on the stability of FP-hTeloC by determining *T*_m_ in the presence of this ligand by both ^19^F NMR and UV/VIS experiments. Intriguingly, ^19^F NMR showed that addition of 0.3 equivalents of mitoxantrone had a small stabilizing effect on the *i*-motif (ΔT_m_ = +1.1°C), whereas higher concentrations had an opposite effect (Δ*T*_m_ = −3.2°C in the presence of two equivalents). UV/VIS studies of FP-hTeloC and hTeloC confirmed the destabilizing effect of excess mitoxantrone on *i*-motif structure (Supplementary Figure S33). Finally, we used ^19^F NMR to study FP-hTeloC and mitoxantrone interaction in a concentration-dependent manner at three different pH values (4.2, 5.5 and 6.4). To that end, ^19^F NMR-monitored titration of FP-hTeloC with mitoxantrone was performed, while AMPF was used as a non-interacting reference. At pH 4.2 and 5.5, *i*-motif signals visible at −79.94 and −79.95 ppm shifted upfield in the presence of one equivalent of mitoxantrone, to −79.97 and −79.99 ppm, respectively (Figure 8A and B). ^19^F NMR spectra at pH_T_ resulted in the appearance of the *i*-motif signal and singe-stranded oligo signal in a 45:55 ratio. Addition of up to one equivalent of mitoxantrone induced changes to both resonances, but especially the one corresponding to *i*-motif (Figure 8C). No interaction between mitoxantrone and the reference AMPF molecule was observed. By plotting the chemical shifts as a function of concentration and fitting a 1:1 binding model, we determined the corresponding apparent dissociation constant equal 0.07 ± 0.03 mM for interaction at pH 4.2 (Figure 8D) which is in the same order of magnitude as the value previously determined by SPR (*K*_d_ 0.12 ± 0.003 mM measured at pH 5.5 in 10 mM sodium cacodylate buffer with 100 mM NaCl, 0.05% Tween-20 and 5% DMSO) (67).

Notably, the interaction of mitoxantrone with the *i*-motif could be observed even at pH 6.4, whereby only a fraction of the probe actually adapted the *i*-motif fold. Moreover, under these conditions ^19^F NMR signal of ssDNA also visibly changed its chemical shift upon mitoxantrone addition (Δ*δ*_F_ = −0.02 ppm), which suggested the ligand weakly interacts with FP-hTeloC sequence also in ssDNA form (Figure 8D).

## DISCUSSION

In this study, we sought to develop ^19^F NMR oligonucleotide probes that combined functional versatility with synthetic accessibility. A careful review of the literature led us to conclude that the key factors for ^19^F NMR probe development were biocompatibility, synthetic availability, responsiveness, sensitivity and application scope. In developing our ^19^F NMR oligonucleotide probes, we focused on FP and FPP moieties, both of which are structurally compact and isosteric with naturally occurring mono- and diphosphate moieties. This was advantageous, as it could ensure highly responsive and biocompatible labels. However, due to the presence of only a single fluorine atom and strong coupling with the phosphorus nucleus (^1^*J*_P-F_ ∼935 Hz) that causes signal splitting into a doublet, the sensitivity of those labels is relatively low. Such low sensitivity could be partially compensated by the synthetic accessibility of such probes, enabling their usage even at high micromolar concentrations.

The first step of our study was the synthesis of 18 deoxyribonucleotide sequences modified at the 5′ end with either FP or FPP moieties (Table 1 and Figure 2). We confirmed that the probes could be synthesized at good yields by adapting methods previously developed for mononucleotides. Importantly, the synthetic protocols were based only on commercially available building blocks for solid-supported synthesis, and as such did not require laborious preparation of modified phosphoramidites, thereby increasing the robustness and scalability of the synthetic protocol.

These fluorinated probes were subsequently evaluated as molecular tools for ^19^F NMR studies. We demonstrated that the probes enabled us to distinguish between ssDNA and dsDNA due to different chemical shift values for those entities. Moreover, the probes could also distinguish between perfect and imperfect duplexes (with a single mismatch at the 3′ end of the complementary strand) independently of their thermodynamic stability (*T*_m_ values), thus overcoming a limitation of many existing probes for mismatch detection. Importantly, a combination of ^19^F NMR signatures from the experiments performed with FP and FPP probes for the same sequence enabled unambiguous determination of every investigated duplex. We believe this finding can be further harnessed to develop assays for different DNA 3′ end-processing enzymatic activities.

We next investigated whether FP and FPP probes could be applied to monitor formation and interactions of non-canonical secondary structures, i.e. G-quadruplexes and *i*-motifs. These structures have previously been investigated by ^19^F NMR, but to the best of our knowledge a universal approach applicable to different non-canonical structures has not been proposed. The influence of FP/FPP moieties did not prevent formation of these secondary structures, albeit it affected their stability. G-quadruplexes were destabilized by FP and FPP moieties, a phenomenon that was observed upon addition of unmodified 5′-phosphate; whereas the stability of *i*-motifs was increased in the presence of FP/FPP. Next, we demonstrated that both G-quadruplex and *i*-motif formation, and either temperature- or pH-dependent transformations, could be detected by ^19^F NMR with the use of our FP and FPP probes. Furthermore, we found that different *i*-motif topologies might also be detected by ^19^F NMR using our probes. ^19^F NMR experiments revealed the formation of more than one topology of the tetrameric *i*-motif structures at 25°C and pH 4.2. Similar phenomena have been previously reported for *i*-motif sequences. As an example, a fluorinated short TC_5_ human telomeric and centromeric DNA sequence incorporating 2′-F-araC has been used to study *i*-motif topology by ^19^F NMR (68). *i*-Motifs are stabilized by acidic conditions when a hemiprotonated cytosine-cytosine^+^ interaction results in two base-paired parallel-stranded duplexes, with fully intercalated nucleobases and antiparallel orientation (69). ^19^F NMR revealed the existence of two possible topologies for this four-stranded *i*-motif form: the R-form and the S-form, in which either the 3′ or the 5′ end cytidine were located outside stacked cytosine-cytosine^+^ pairs (63,70). This phenomenon has also been examined by gel electrophoresis (68).

Finally, we demonstrated that ^19^F NMR could detect the interaction between these secondary structures and either proteins or small molecules. The interaction with proteins was demonstrated by means of a TBA sequence (ON11) and thrombin (Figure 5). This sort of experiment may be performed to verify results obtained by other biophysical methods, as well as for the development of ^19^F NMR-based screening assays to identify small molecules targeting nucleic acid-binding proteins. The ability to monitor nucleic acid interaction with small molecules by means of FP/FPP probes was exemplified using two *i*-motif structures and their previously identified ligands: TMPyP4 and mitoxantrone (65,71). So far, the discovery of small molecules targeting G-quadruplexes and *i*-motifs has relied mostly on probes emitting fluorescent light upon intercalating these secondary structures (66,72). However, such competition assays are prone to fluorescence-associated interference and may lead to both false positive and negative results. As demonstrated here, ^19^F NMR experiments enable direct observation of the interaction with small molecules due to chemical shift perturbation. Therefore, ^19^F NMR experiments with our probes could be used in the future to validate hits from fluorescent assays, characterize binding affinities for different target sequences, as well as to develop fluorescence-independent high-throughput screening experiments aimed at identifying nucleic acid-binding ligands and verifying their specificity for a particular sequence or topology.

Further studies are required to determine whether FP and FPP labels are suitable for investigation of other types of nucleic acid-related phenomena (e.g. hairpin formation, RNA–DNA duplex formation, duplex versus triplex identification) and labeling of different types of nucleic acids (e.g. RNA, PNA).

## Supplementary Material

gkaa470_Supplemental_Files
