# Supplementary material for: 5′-fluoro(di)phosphate-labeled oligonucleotides are versatile molecular probes for studying nucleic acid secondary structure and interactions by 19F NMR
Source: Nucleic Acids Res. 2020 Jun 9;48(15):8209–24. doi: 10.1093/nar/gkaa470 (PMC7470941; doi:10.1093/nar/gkaa470)
Supplement: gkaa470_Supplemental_Files [file gkaa470_supplemental_files.zip › Supplementary Information 1 - Figures and Tables.pdf]

Supplementary Information 1

5′-fluoro(di)phosphate-labeled oligonucleotides are versatile molecular probes for studying nucleic acid secondary structure and interactions by ^{19}F NMR

Marek R. Baranowski[1], Marcin Warminski[1], Jacek Jemielity[2] and Joanna Kowalska[1,*]

[1] Division of Biophysics, Institute of Experimental Physics, Faculty of Physics, University of Warsaw, Ludwika Pasteura 5, 02-093 Warsaw, Poland
[2] Centre of New Technologies, University of Warsaw, Stefana Banacha 2c, 02-097 Warsaw, Poland

Table of contents

Synthesis of oligonucleotides 4

Table S1. Summary of synthesized unmodified DNAs. 4

Table S2. Sequences of all synthesized oligonucleotides and their applications in ^{19}F NMR studies. 5

UV/VIS duplex formation monitoring 6

Figure S1. UV/VIS duplex formation monitoring of FPPON14 and ON4 or ON25. 6

Figure S2. UV/VIS duplex formation monitoring of FPON3 and ON5, ON9, ON20–ON24. 6

Figure S3. UV/VIS duplex formation monitoring of FPPON3 and ON5, ON9, ON20–ON24. 7

Figure S4. UV/VIS duplex formation monitoring of ON3 and ON5, ON9, ON20–ON24....... 7

^{19}F NMR duplex formation monitoring 8

Figure S5. ^{19}F NMR duplex formation monitoring for FPPON3 and ON9. 8

Figure S6. ^{19}F NMR duplex formation monitoring for FPPON3 and ON5. 8

Figure S7. ^{19}F NMR duplex formation monitoring for FPPON3 and ON20. 9

Figure S8. ^{19}F NMR duplex formation monitoring for FPPON3 and ON21. 9

Figure S9. ^{19}F NMR duplex formation monitoring for FPPON3 and ON22. 10

Figure S10. ^{19}F NMR duplex formation monitoring for FPPON3 and ON23. 10

Figure S11. ^{19}F NMR duplex formation monitoring for FPPON3 and ON24. 11

Figure S12. ^{19}F NMR duplex formation monitoring for FPON3 and ON9. 11

Figure S13. ^{19}F NMR duplex formation monitoring for FPON3 and ON5. 12

Figure S14. ^{19}F NMR duplex formation monitoring for FPON3 and ON20. 12

Figure S15. ^{19}F NMR duplex formation monitoring for FPON3 and ON21. 13

Figure S16. ^{19}F NMR duplex formation monitoring for FPON3 and ON22. 13

Figure S17. ^{19}F NMR duplex formation monitoring for FPON3 and ON23. 14

Figure S18. ^{19}F NMR duplex formation monitoring for FPON3 and ON24. 14

G-quadruplex formation studies 15

Figure S19. ^{1}H NMR spectra of FPPON11 at different temperatures. 15

Figure S20. Monitoring thrombin (THPH1)-FPPON11 interaction by ^{19}F NMR. 15

***i*-Motifs formation studies** 16

Figure S21. ^{1}H NMR spectra of *i*-motif sequences. 16

Figure S22. ^{19}F NMR spectrum of FPON1 (A) and FPPON1 (B). 16

Figure S23. ^{19}F NMR spectra of FPPON1 *i*-motif sequence at different temperatures. 17

Figure S24. ^{19}F NMR spectra of FPON1 *i*-motif sequence at different temperatures......... 17

Figure S25. UV/VIS formation monitoring of short (TCCCCC) *i*-motif sequences at pH 4.2 18

Figure S26. ^{19}F NMR spectra of 0.5 mM FPON1 and 0.1 mM FPPON1 at different time after dilution in buffer....... 19

Figure S27. ^{19}F NMR interaction monitoring of FPON1 and TMPyP4....... 20

Figure S28. ^{19}F NMR spectra of FP-hTeloC *i*-motif sequence in different pH. 20

Figure S29. Melting temperature determination of FP-hTeloC at pH 4.2 by ^{19}F NMR....... 21

Figure S30. Melting temperature determination of FP-hTeloC at pH 5.5 by ^{19}F NMR....... 21

Figure S31. UV/VIS formation monitoring of FP-hTeloC and ON_I in pH 4.2. 22

Figure S32. UV/VIS formation monitoring of hTeloC *i*-motif sequences in pH 5.5....... 22

Figure S33. Influence of Mitoxantrone on T_m values of FP-hTeloC and hTeloC. 23

Table S3. Transitions pH (pH_T) and melting temperatures (T_m) values of TCCCCC taken from literature. 23

Table S4. Melting temperatures (T_m) values of TCC CCC and (TAA $CCC)_4$ determined by UV/VIS....... 24

Table S5. Transitions pH (pH_T) and melting temperatures (T_m) values of hTeloC taken from literature. 24

References 25

Synthesis of oligonucleotides

Table S1. Summary of synthesized unmodified DNAs.

Oligo name	Length (nt)	Sequence (5′→3′)	Isolated yield[a]	Molecular formula of $[M-nH]^{n-}$ ion	Calc. (m/z)	Actual (m/z)
ON1	6	TCC CCC	38%	$C_{55}H_{72}N_{17}O_{35}P_5^{2-}$	842.65379	842.65435
hTeloC	24	TAA CCC TAA CCC TAA CCC TAA CCC	30%	$C_{228}H_{289}N_{84}O_{138}P_{23}^{4-}$	1780.80415	1780.80475
ON3	10	AGA CAT TGA C	16%	$C_{99}H_{121}N_{40}O_{56}P_9^{3-}$	1010.51423	1010.51660
ON4	10	TGA CAT TGA C	35%	$C_{98}H_{121}N_{37}O_{58}P_9^{3-}$	1007.51037	1007.51482
ON5	10	GTC AAT GTC C	20%	$C_{97}H_{121}N_{35}O_{59}P_9^{3-}$	999.50662	999.51110
ON9	10	GTC AAT GTC T	29%	$C_{98}H_{122}N_{34}O_{60}P_9^{3-}$	1004.50652	1004.51057
ON11	15	GGT TGG TGT GGT TGG	57%	$C_{150}H_{184}N_{57}O_{94}P_{14}^{3-}$	1573.59044	1573.59159
PON11[b]	15	P-GGT TGG TGT GGT TGG	42%	$C_{150}H_{185}N_{57}O_{97}P_{15}^{3-}$	1600.24588	1600.24497
ON20	10	GTC AAT GTC G	27%	$C_{98}H_{121}N_{37}O_{59}P_9^{3-}$	1012.84201	1012.84612
ON21	10	GTC AAT GTC A	25%	$C_{98}H_{121}N_{37}O_{58}P_9^{3-}$	1007.51037	1007.51098
ON22	10	GTC AAT GTT T	40%	$C_{99}H_{123}N_{33}O_{61}P_9^{3-}$	1009.50640	1009.50718
ON23	10	GTC AAT GTG T	20%	$C_{99}H_{122}N_{36}O_{60}P_9^{3-}$	1017.84190	1017.84319
ON24	10	GTC AAT GTA T	30%	$C_{99}H_{122}N_{36}O_{59}P_9^{3-}$	1012.51026	1012.51180
ON25	10	TGA CAT TGA T	32%	$C_{99}H_{122}N_{36}O_{59}P_9^{3-}$	1012.51026	1012.51121

[a] All unmodified oligonucleotides were synthesized on 2 μmole scale; [b] P refers to phosphate group.

Table S2. Sequences of all synthesized oligonucleotides and their applications in ^{19}F NMR studies.

No.	Oligonucleotide name	Sequence (5′→3′)	5′-group
	**Oligonucleotides used as molecular probes in ^{19}F NMR studies**		
	Oligonucelotide used for *i*-motif formation monitoring		
1	FPPON1	TCCC CC	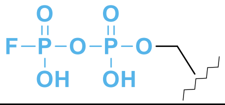
2	FPON1	TCCC CC	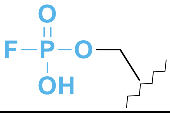
3	FP-hTeloC	TAA CCC TAA CCC TAA CCC TAA CCC	
	Oligonucleotides used in monitoring of duplex formation and mismatch identification		
4	FPON3	AGA CAT TGA C	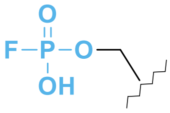
5	FPPON3	AGA CAT TGA C	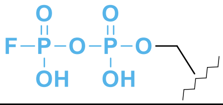
6	FPPON14	GTC AAT GTC AGC GAT A	
	Oligonucleotide used in monitoring of G-quadruplex formation and protein-nucleic acid interaction		
7	FPPON11	GGT TGG TGT GGT TGG	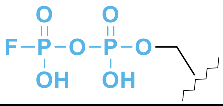
	**Other fluorodiphosphorylated oligonucleotides**		
8	FPPON2	GTC AAT G	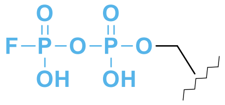
9	FPPON4	TGA CAT TGA C	
10	FPPON5	GTC AAT GTC C	
11	FPPON6	GGA CAT TGA C	
12	FPPON7	ATC AAT GTC G	
13	FPPON8	CGA CAT TGA T	
14	FPPON9	GTC AAT GTC T	
15	FPPON10	GTC AAT GTC AGC G	
16	FPPON12	GGA TAC TTT TGT ATC C	
17	FPPON13	GGA TAT TTT TAT ATC C	
18	FPPON15	CCT GGG GGA GTA TTG CGG AGG AAG G	
	**Unmodified oligonucleotides**		
19	ON1	TCC CCC	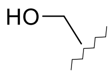
20	hTeloC	TAA CCC TAA CCC TAA CCC TAA CCC	
21	ON3	AGA CAT TGA C	
22	ON4	TGA CAT TGA C	
23	ON5	GTC AAT GTC C	
24	ON9	GTC AAT GTC T	
25	ON11	GGT TGG TGT GGT TGG	
26	ON20	GTC AAT GTC G	
27	ON21	GTC AAT GTC A	
28	ON22	GTC AAT GTT T	
29	ON23	GTC AAT GTG T	
30	ON24	GTC AAT GTA T	
31	ON25	TGA CAT TGA T	
32	PON11	GGT TGG TGT GGT TGG	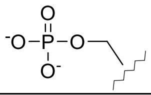

UV/VIS duplex formation monitoring

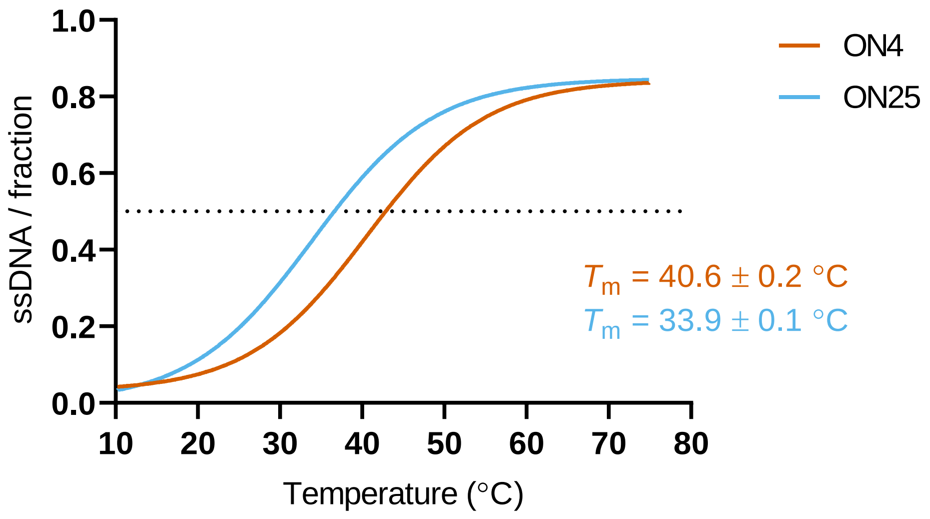

Figure S1. UV/VIS duplex formation monitoring of FPPON14 and ON4 or ON25. For clarity only fitted curves are shown.

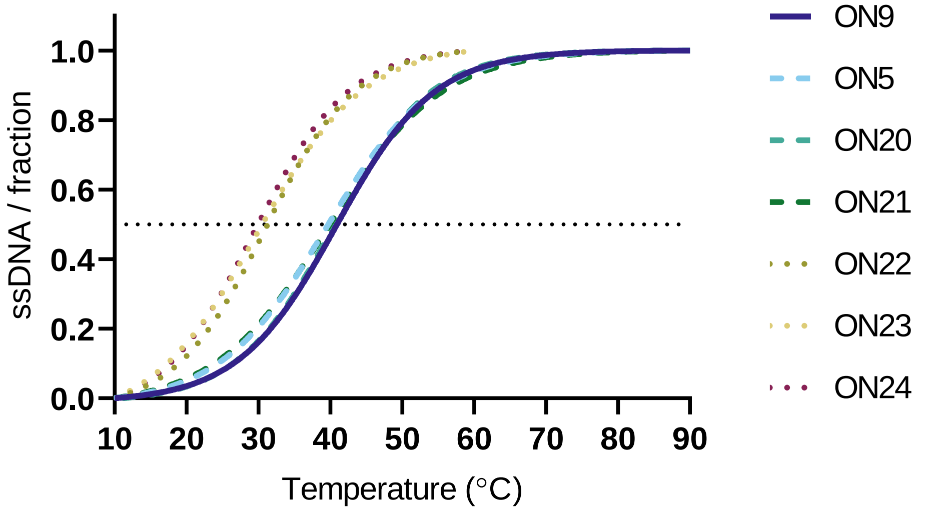

Figure S2. UV/VIS duplex formation monitoring of FPON3 and ON5, ON9, ON20–ON24. For clarity only fitted curves are shown.

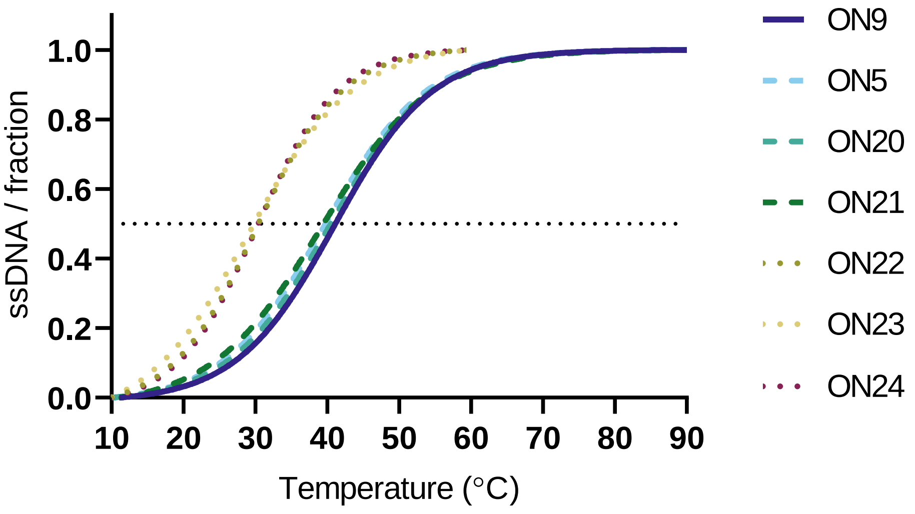

Figure S3. UV/VIS duplex formation monitoring of FPPON3 and ON5, ON9, ON20–ON24. For clarity only fitted curves are shown.

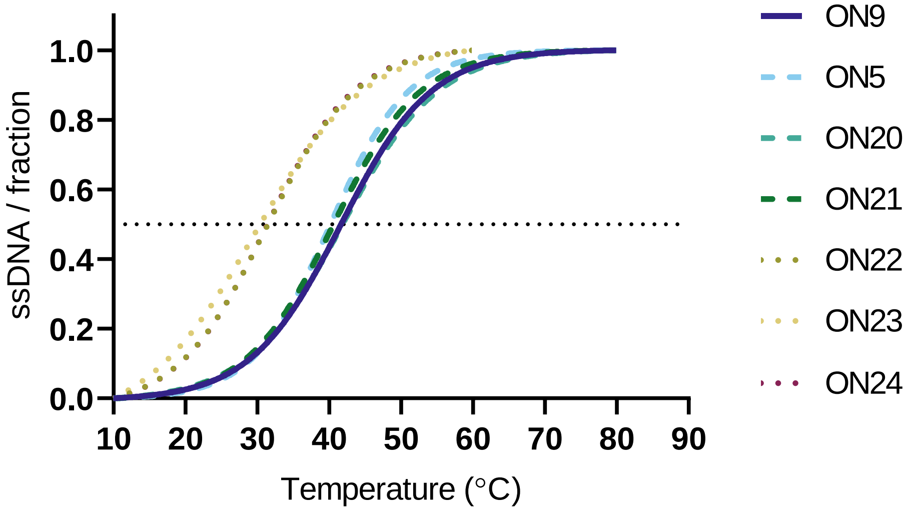

Figure S4. UV/VIS duplex formation monitoring of ON3 and ON5, ON9, ON20–ON24. For clarity only fitted curves are shown.

^{19}F NMR duplex formation monitoring

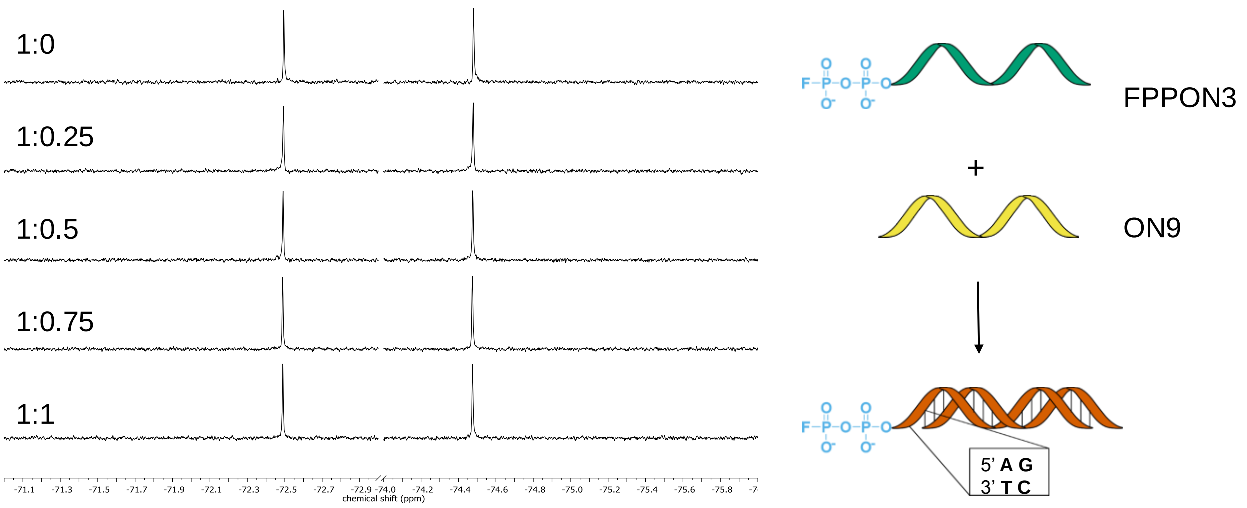

Figure S5. ^{19}F NMR duplex formation monitoring for FPPON3 and ON9.

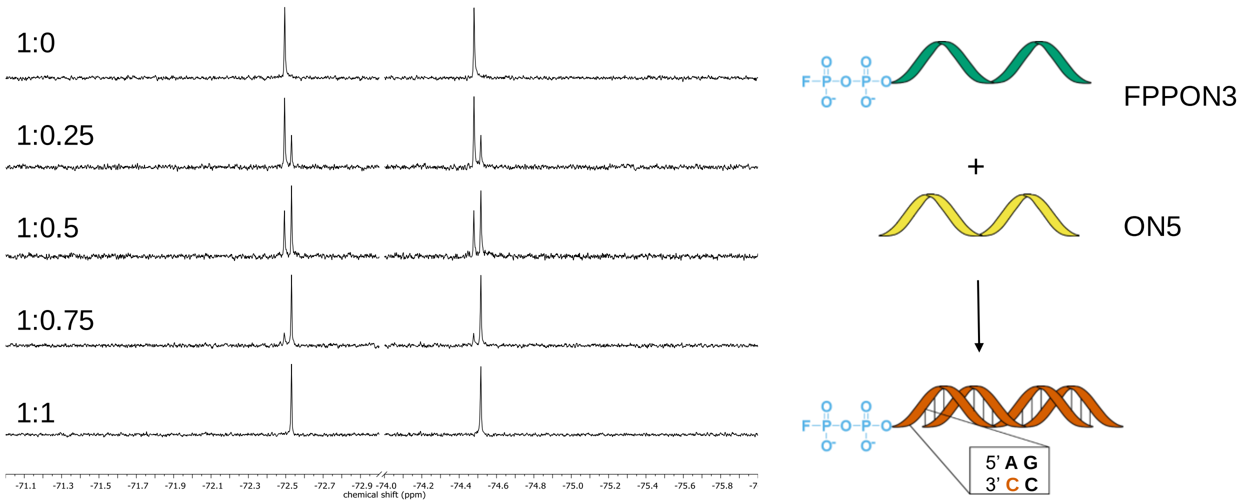

Figure S6. ^{19}F NMR duplex formation monitoring for FPPON3 and ON5.

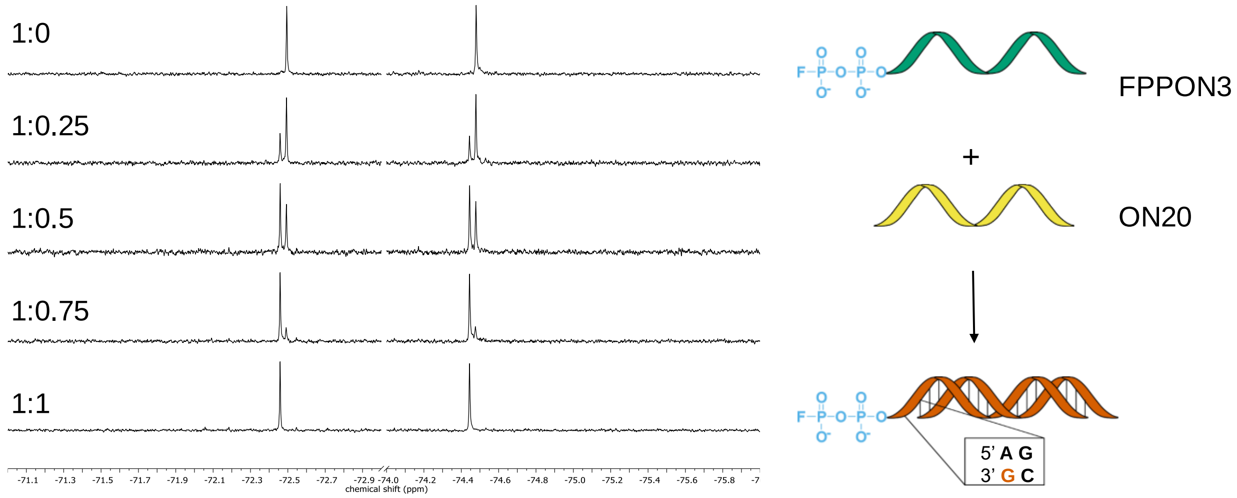

Figure S7. ^{19}F NMR duplex formation monitoring for FPPON3 and ON20.

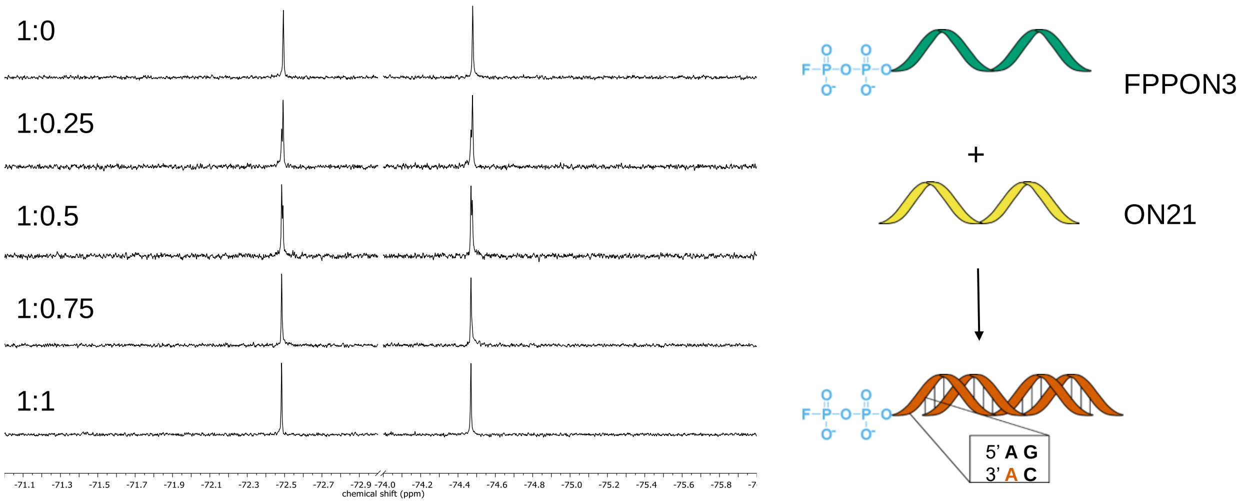

Figure S8. ^{19}F NMR duplex formation monitoring for FPPON3 and ON21.

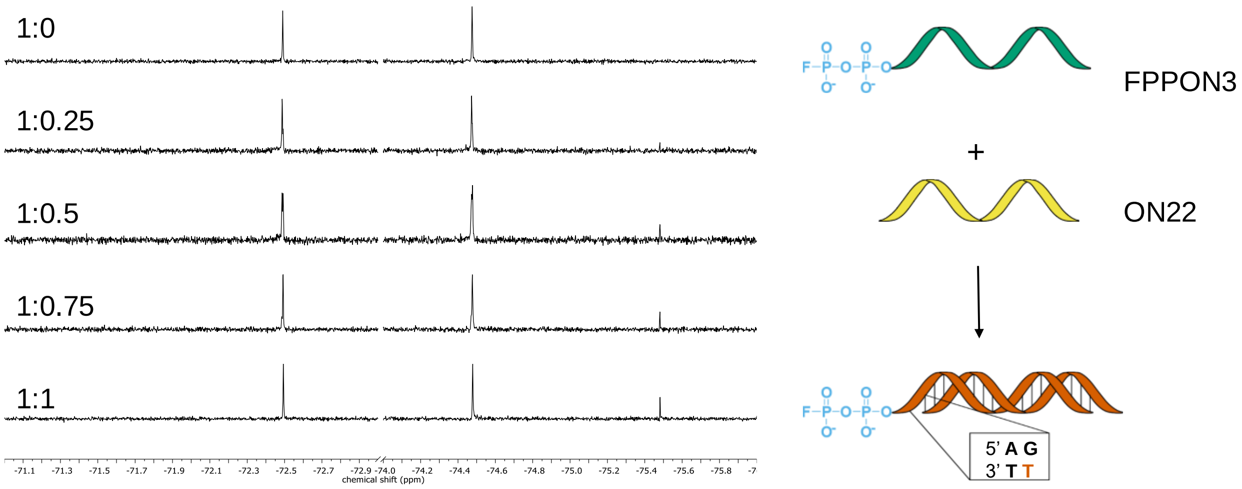

Figure S9. ^{19}F NMR duplex formation monitoring for FPPON3 and ON22.

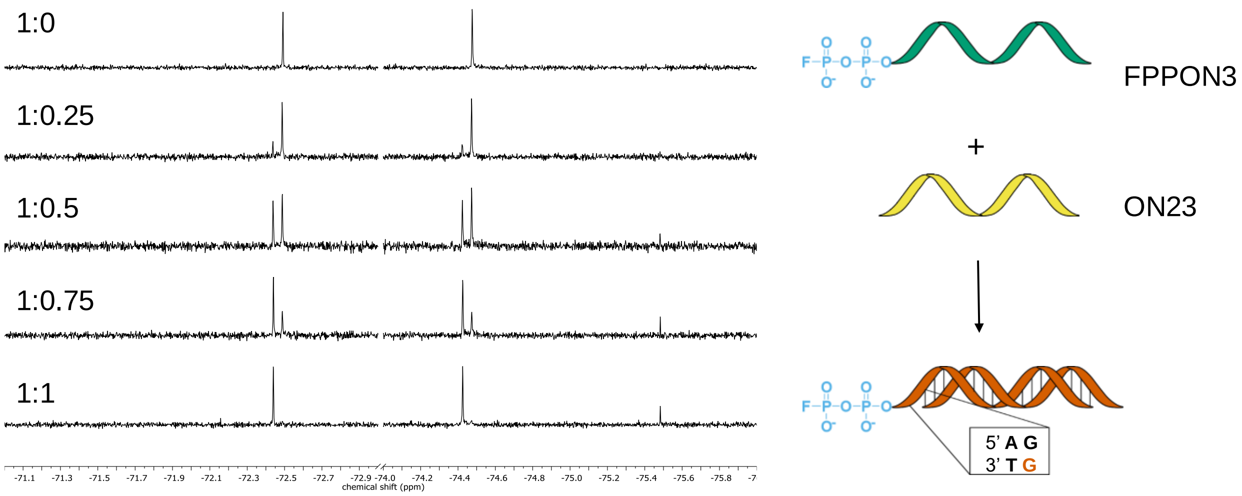

Figure S10. ^{19}F NMR duplex formation monitoring for FPPON3 and ON23.

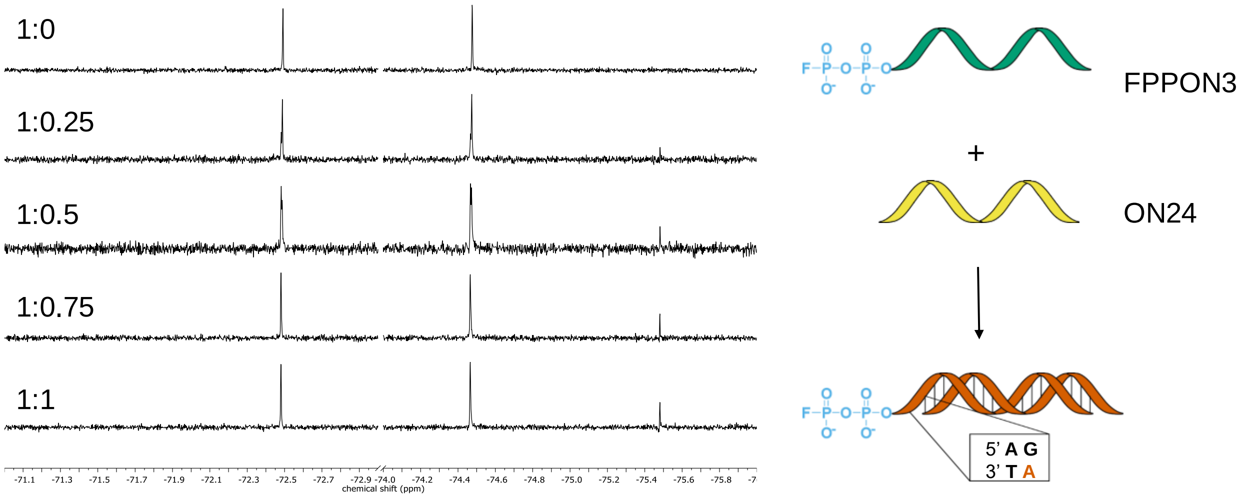

Figure S11. ^{19}F NMR duplex formation monitoring for FPPON3 and ON24.

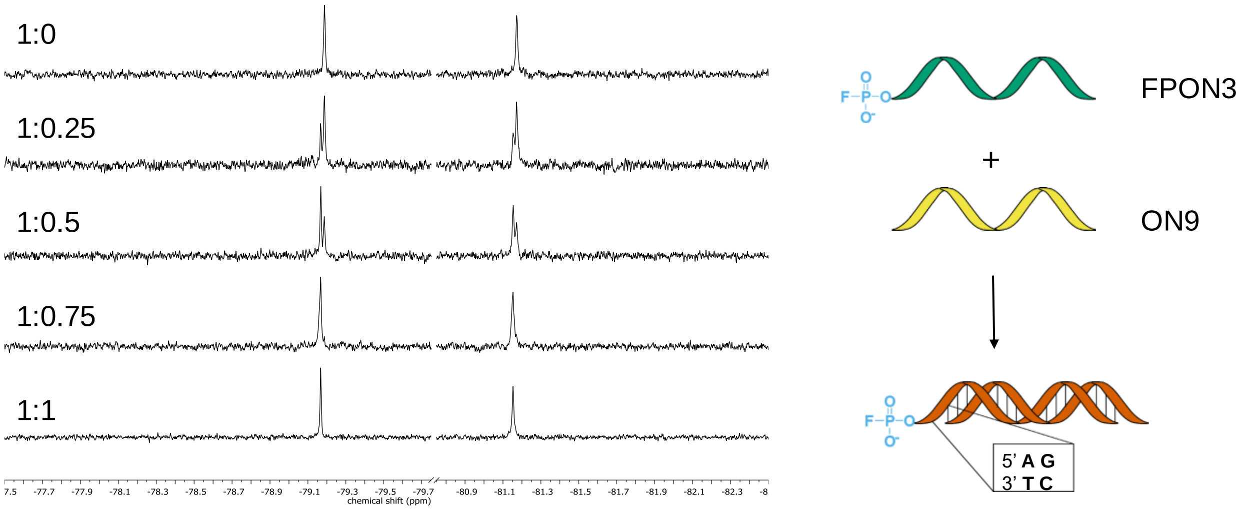

Figure S12. ^{19}F NMR duplex formation monitoring for FPON3 and ON9.

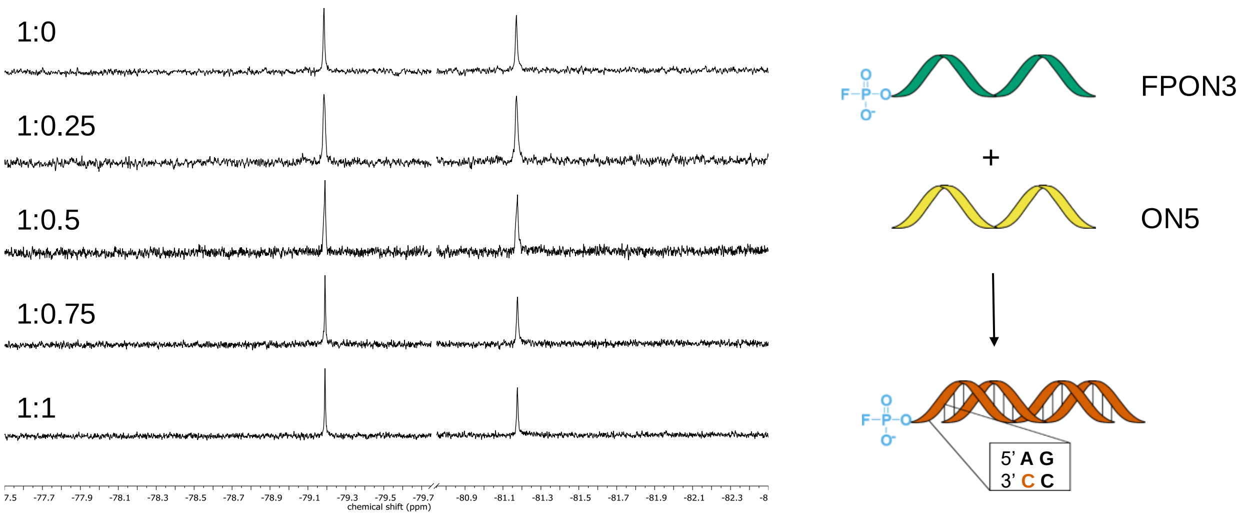

Figure S13. ^{19}F NMR duplex formation monitoring for FPON3 and ON5.

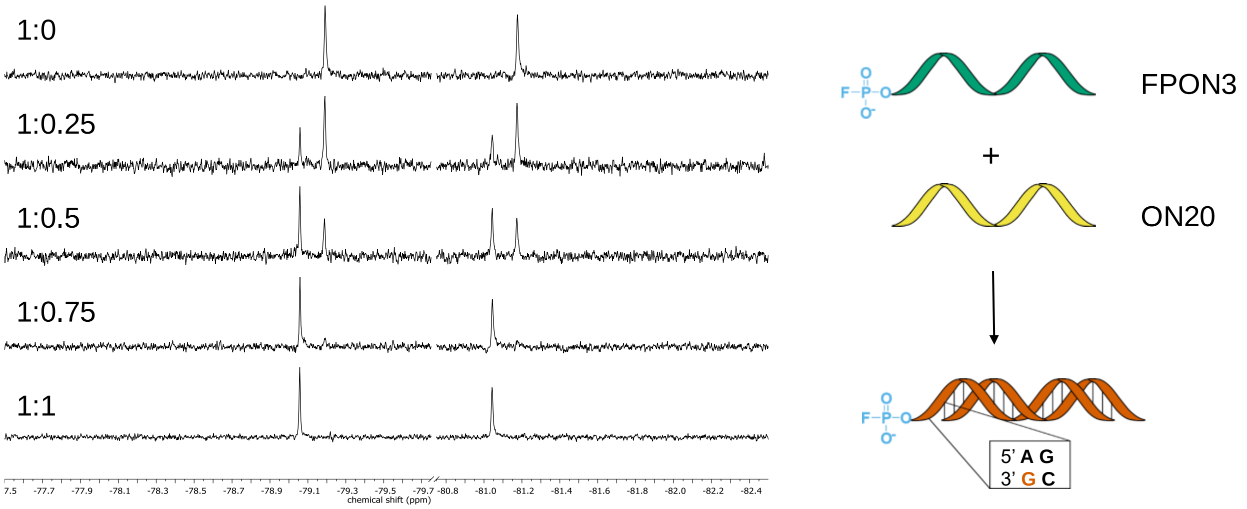

Figure S14. ^{19}F NMR duplex formation monitoring for FPON3 and ON20.

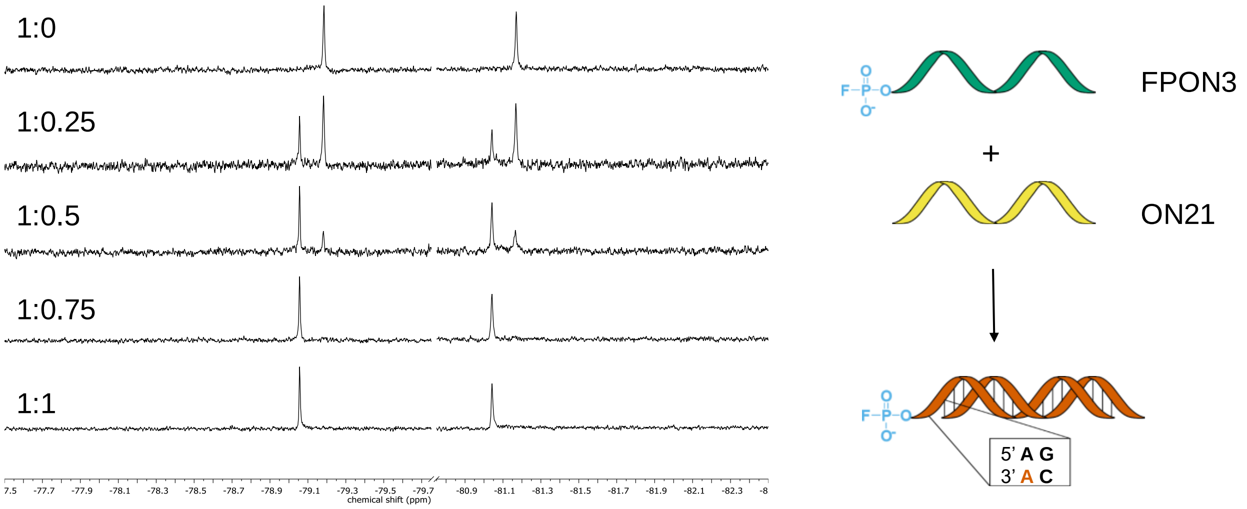

Figure S15. ^{19}F NMR duplex formation monitoring for FPON3 and ON21.

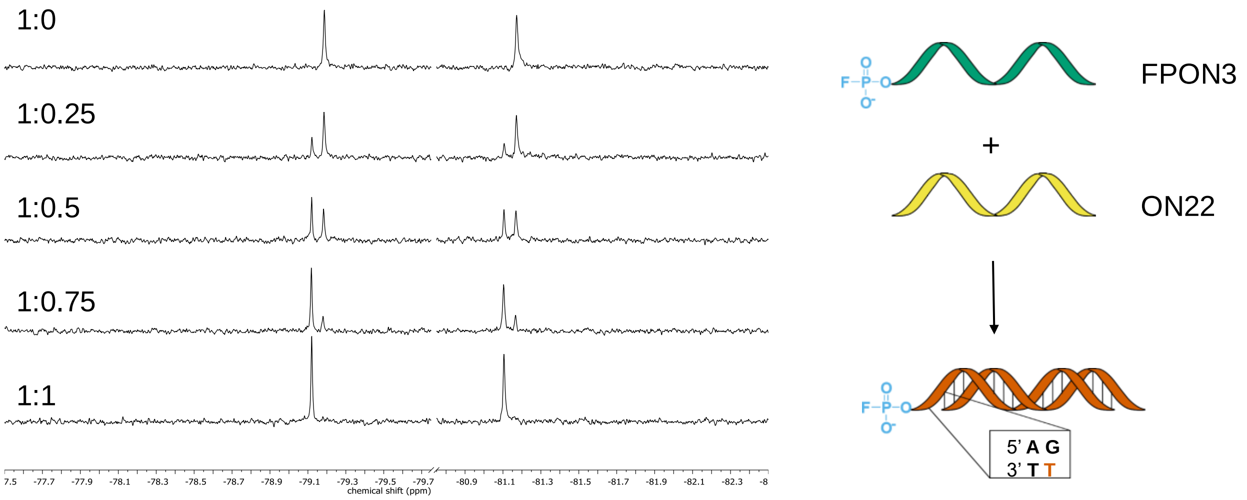

Figure S16. ^{19}F NMR duplex formation monitoring for FPON3 and ON22.

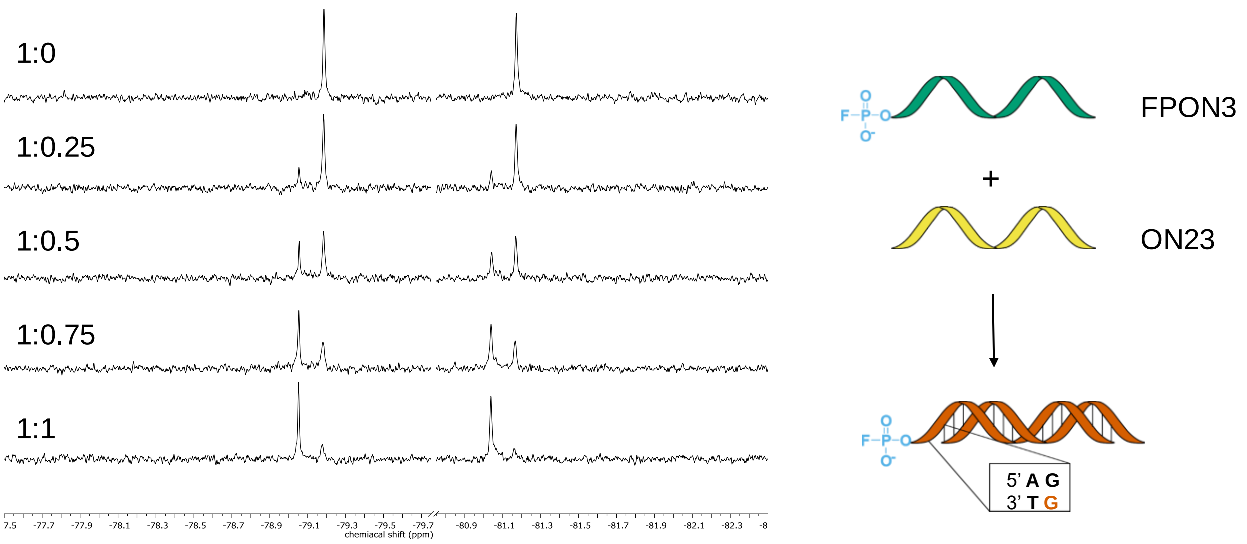

Figure S17. ^{19}F NMR duplex formation monitoring for FPON3 and ON23.

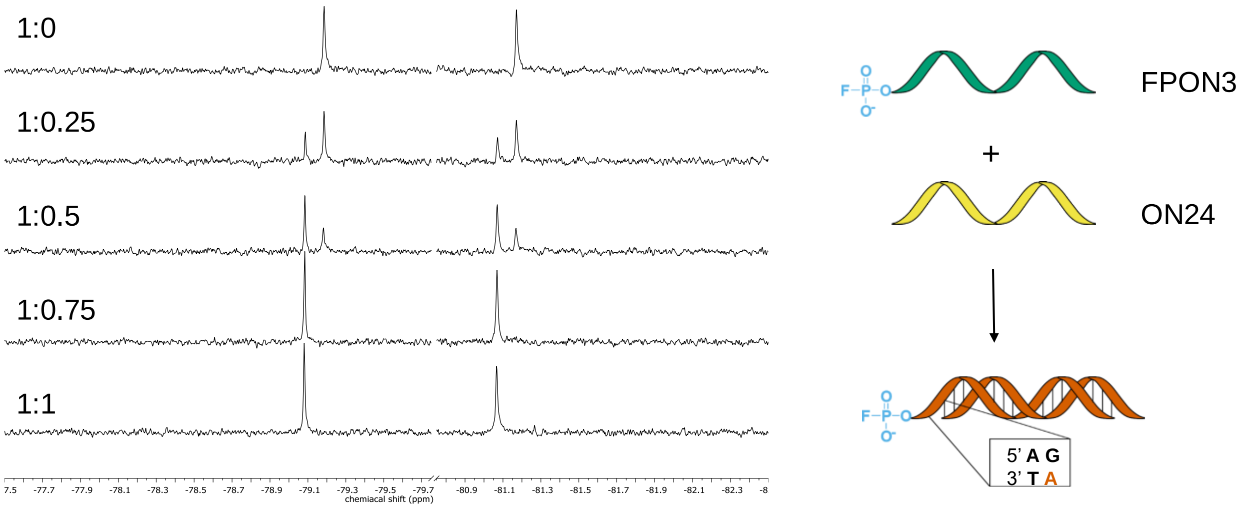

Figure S18. ^{19}F NMR duplex formation monitoring for FPON3 and ON24.

G-quadruplex formation studies

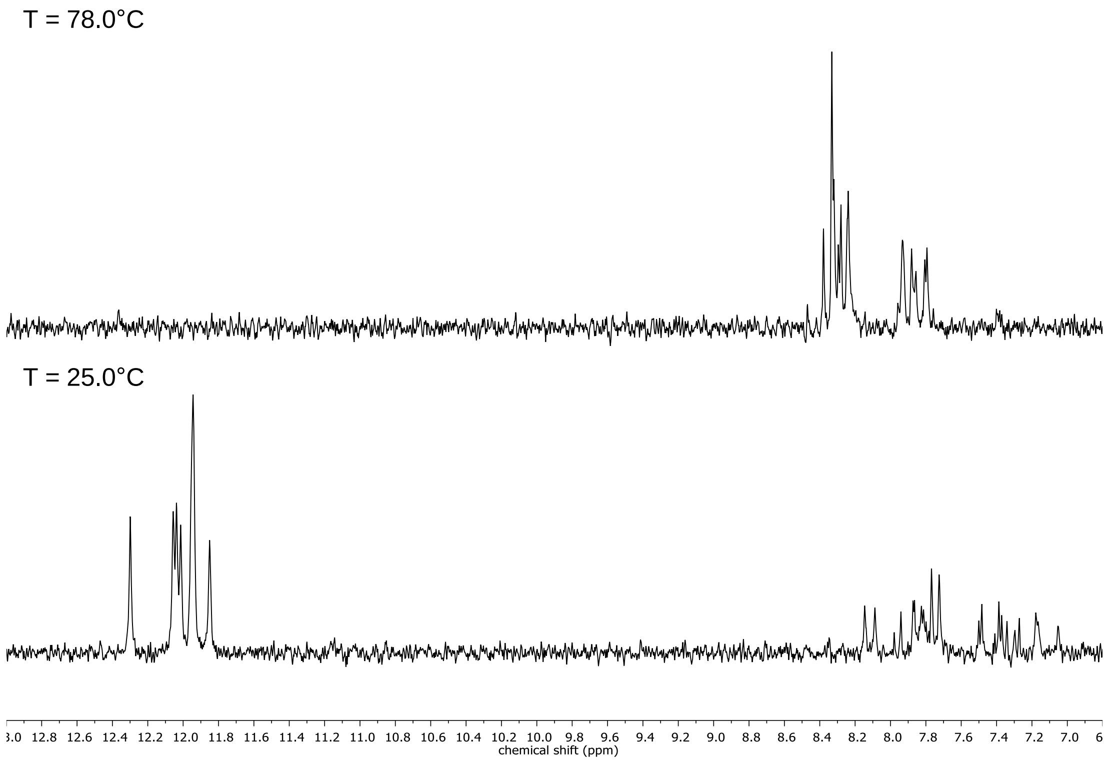

Figure S19. ^{1}H NMR spectra of FPPON11 at different temperatures.
FPPON11 (70 μM) in 90% H_2O/10 % D_2O (10 mM KH_2PO_4, 70 mM KCl, 0.2 mM EDTA, pH=7.0). Hydrogen-bonded imino resonances are clearly visible at 25°C. Imino protons are clearly visible at 12 ppm which confirms G-quadruplex formation in selected conditions.

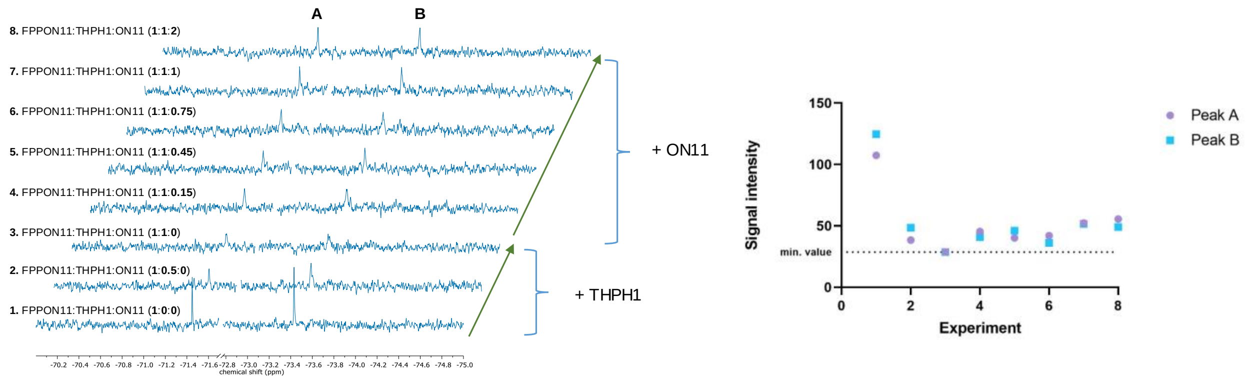

Figure S20. Monitoring thrombin (THPH1)-FPPON11 interaction by ^{19}F NMR.
70 μM of FPPON11 in 90% H_2O/10 % D_2O (10 mM KH_2PO_4, 70 mM KCl, 0.2 mM EDTA, pH=7.0).

i-Motifs formation studies

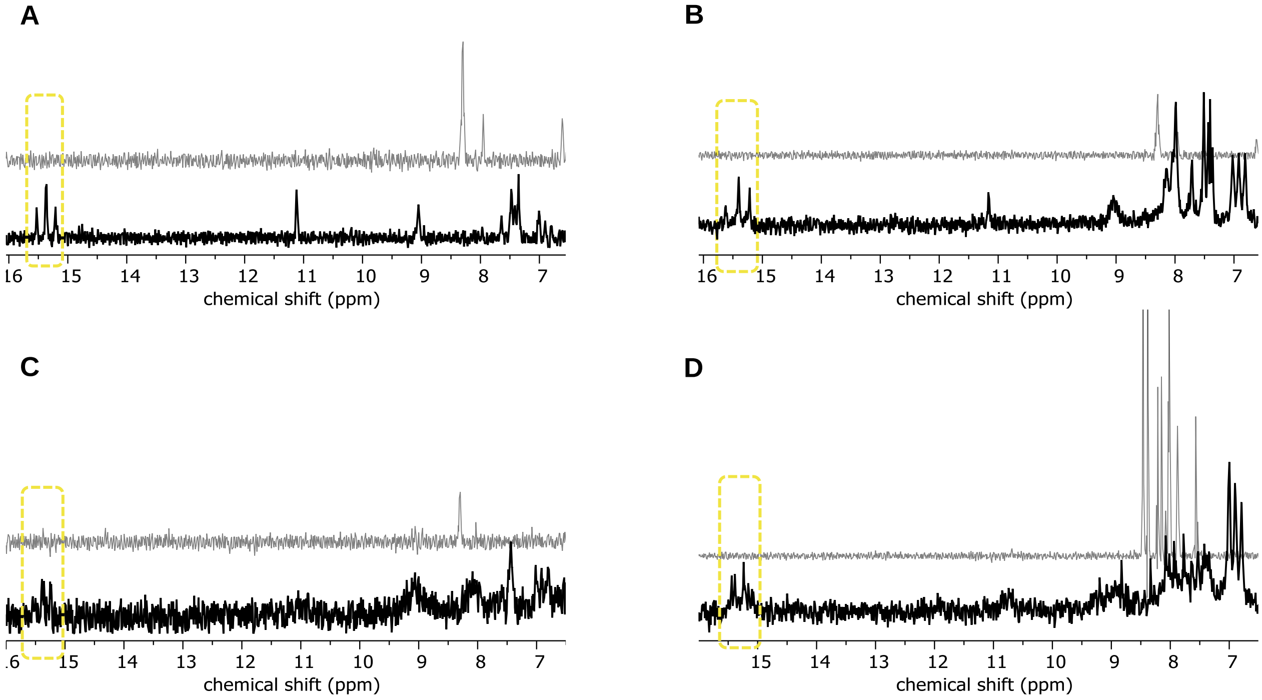

Figure S21. ^{1}H NMR spectra of *i*-motif sequences.
^{1}H NMR spectra at 10 °C (black line) and at 70 °C (grey line) in 50 mM sodium citrate buffer, 10% D_2O. (**A**) 0.5 mM FPON1, pH 4.20, 2048 scans; (**B**) 0.5 mM ON1, 2048 scans; (**C**) 0.1 mM FPPON1, 10240 scans; grey line: 70 °C; (**D**) 0.25 mM FP-hTeloC, pH 4.20, 4096 scans;

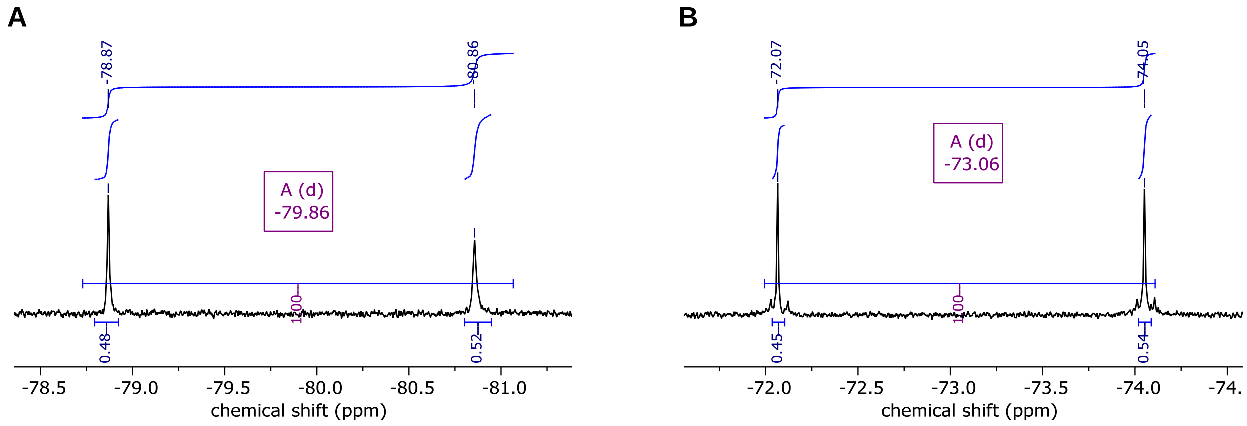

Figure S22. ^{19}F NMR spectrum of FPON1 (A) and FPPON1 (B).
Conditions: 0.5 mM FPON1, 0.1 mM FPPON1, 50 mM sodium citrate buffer pH 4.2

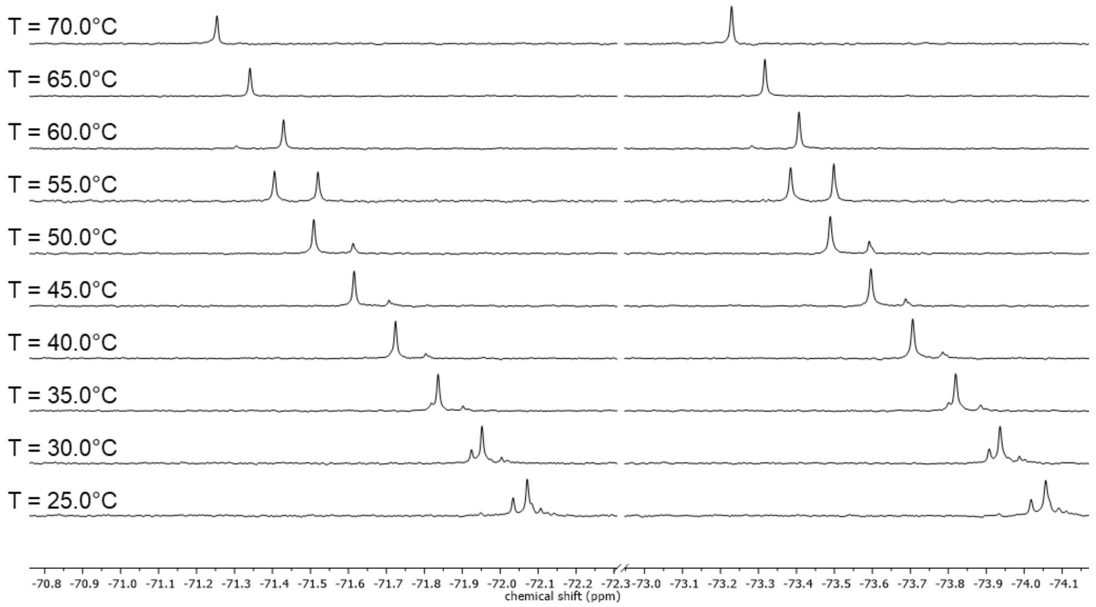

Figure S23. ^{19}F NMR spectra of FPPON1 *i*-motif sequence at different temperatures. Conditions: 100 μM FPPON1, 50 mM citrate buffer pH=4.20, 10% D_2O.

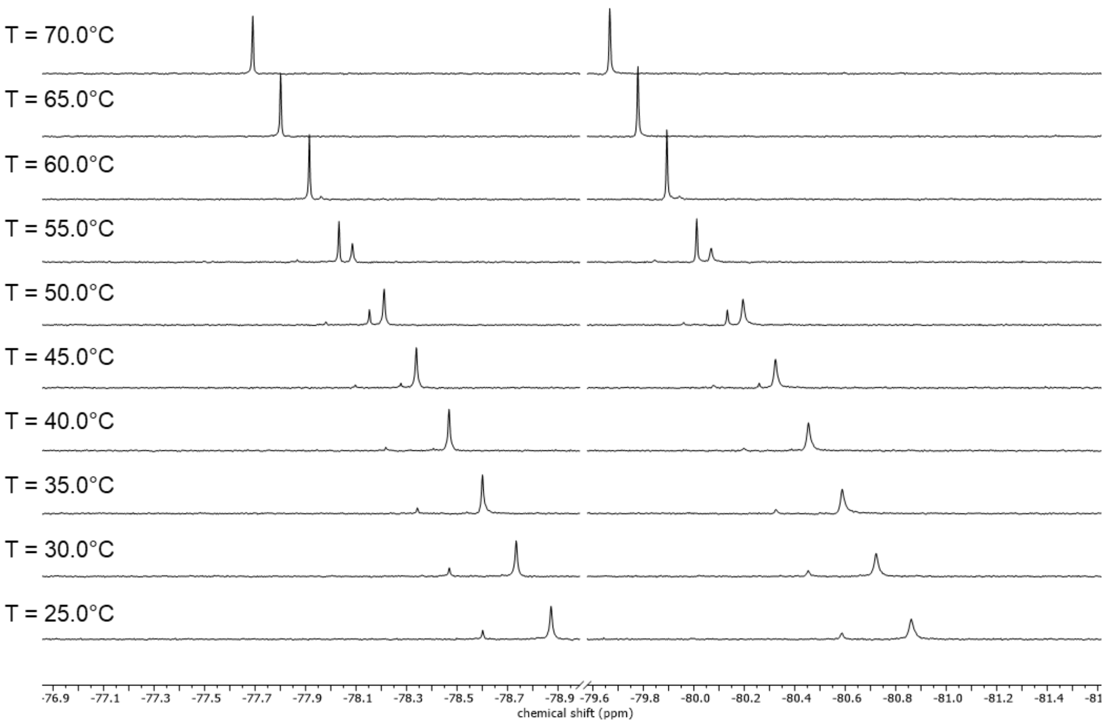

Figure S24. ^{19}F NMR spectra of FPON1 *i*-motif sequence at different temperatures. Conditions: 500 μM FPON1, 50 mM citrate buffer pH=4.20, 10% D_2O.

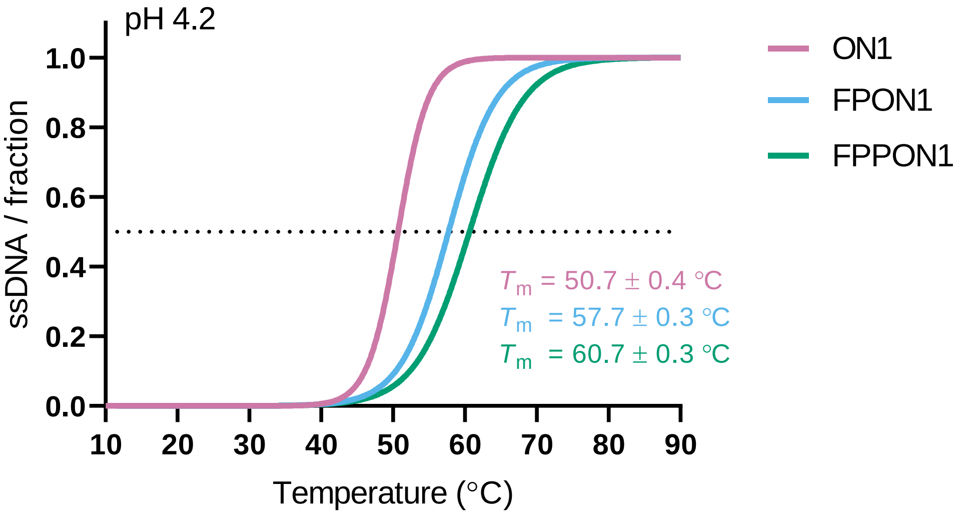

Figure S25. UV/VIS formation monitoring of short (TCCCCC) *i*-motif sequences at pH 4.2 Conditions: 12 μM of each sequence, 50 mM citrate buffer, pH 5.50, 10% D_2O; Absorbance at 260 nm was measured.

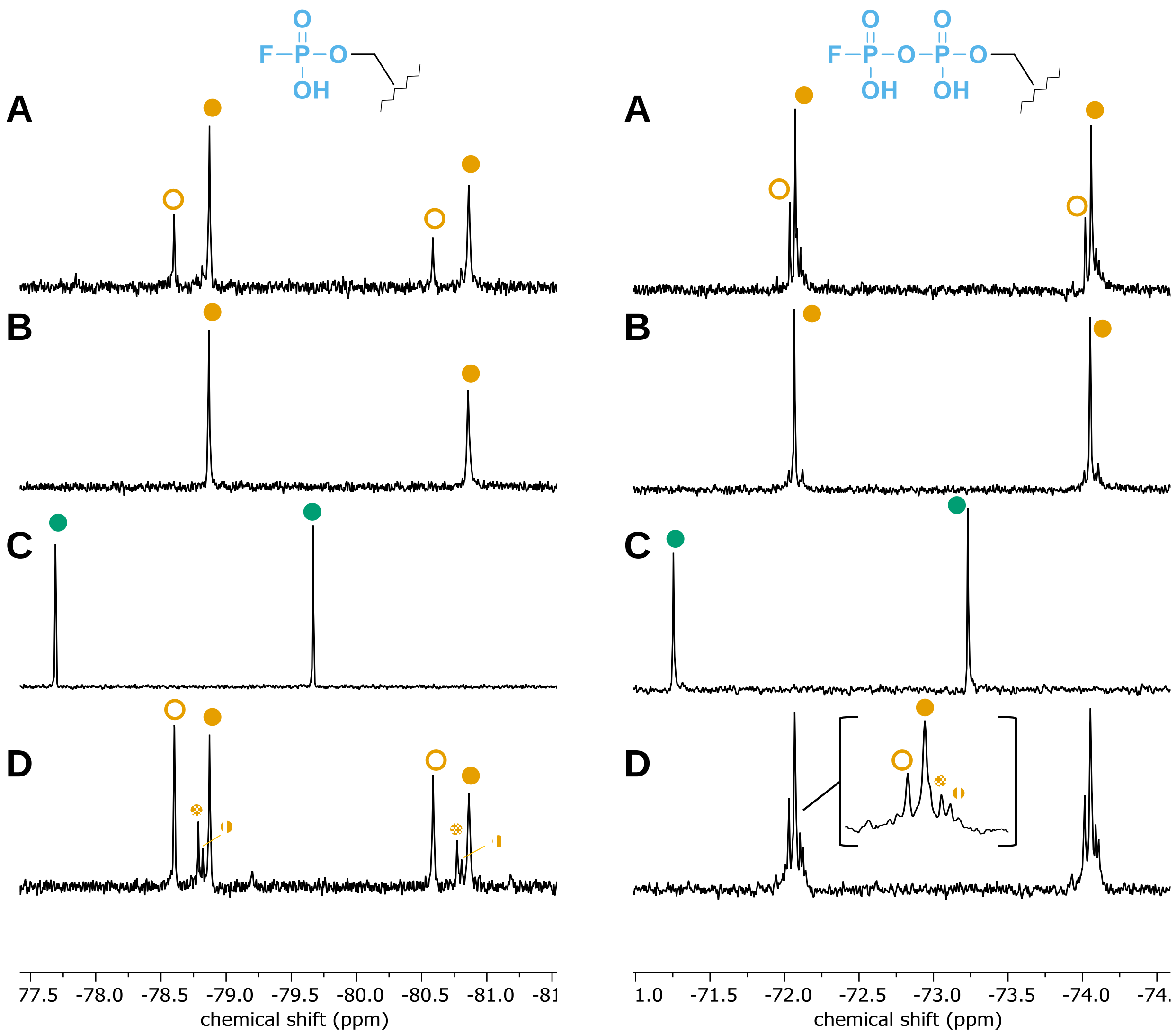

Figure S26. ^{19}F NMR spectra of 0.5 mM FPON1 and 0.1 mM FPPON1 at different time after dilution in buffer.
(**A**) Spectra recorded after 1h of dilution oligo in buffer (**B**) The same sample as in A but after 48h in rt; (**C**) Oligo at 70 °C; (**D**) The same sample as in C but spectra recorded after decreasing temperature to 25 °C. Orange dots indicate *i*-motif structures, green dots indicate ssDNA. Conditions: 50 mM sodium citrate buffer pH 4.20, 10% D_2O.

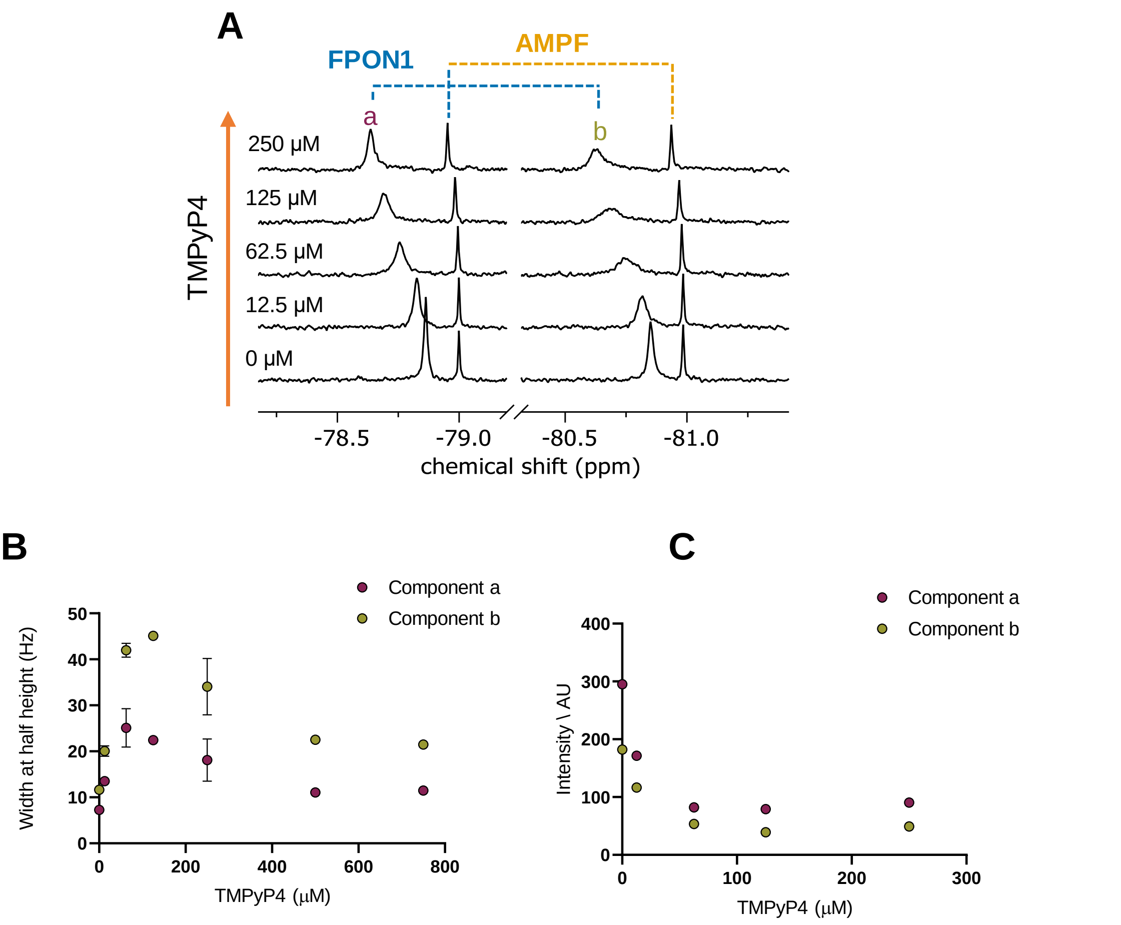

Figure S27. ^{19}F NMR interaction monitoring of FPON1 and TMPyP4.
A) ^{19}F NMR signal changes upon titration with TMPyP4; B) and C) Width at the half height and intensity changes of ^{19}F NMR signal. Conditions: 50 mM sodium citrate buffer pH 4.2, 10% D_2O, 25 °C.

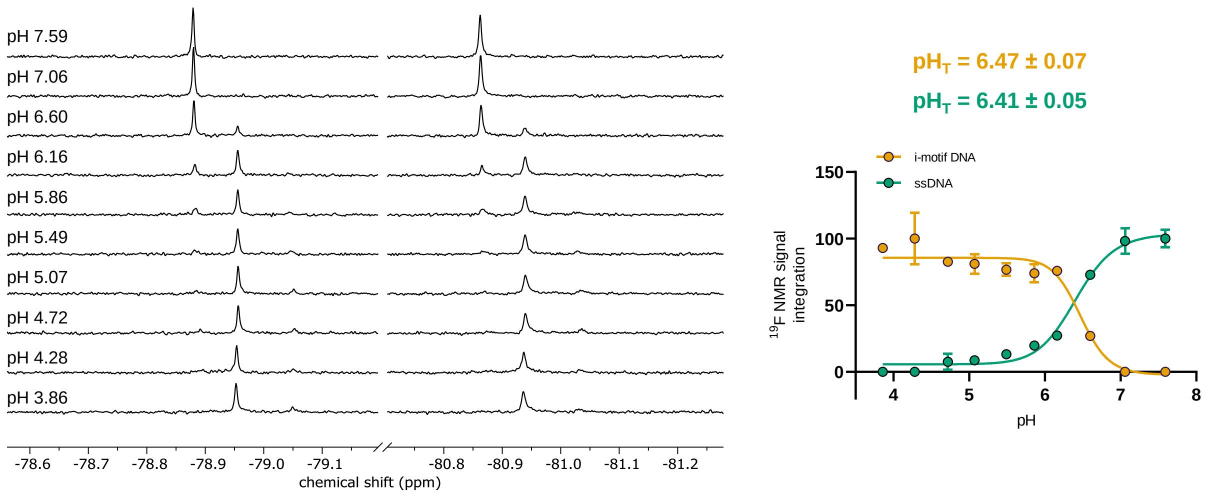

Figure S28. ^{19}F NMR spectra of FP-hTeloC *i*-motif sequence in different pH.
Conditions: 250 µM Fp-hTeloC, 50 mM citrate buffer, 10% D_2O, 25 °C

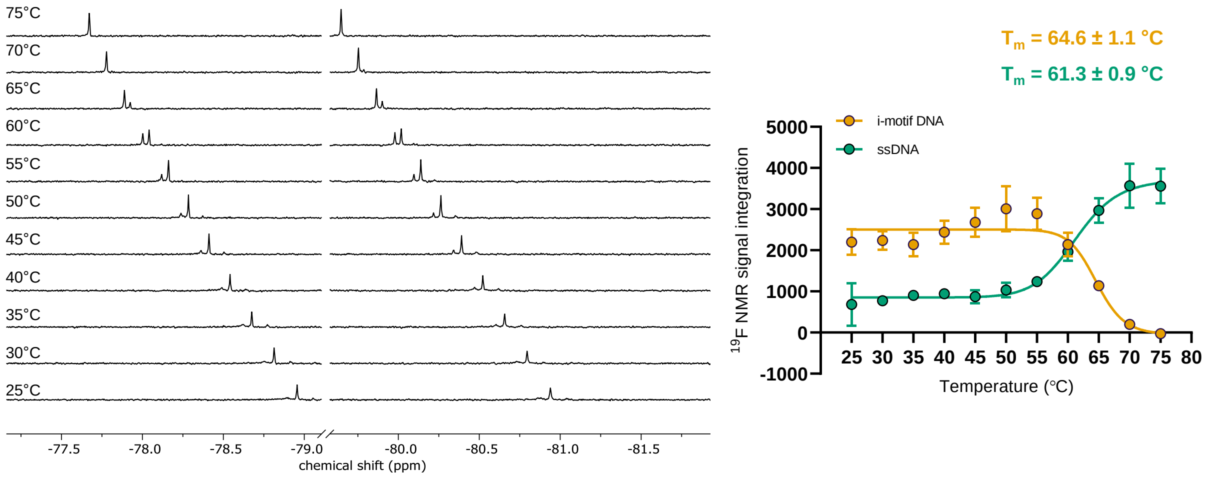

Figure S29. Melting temperature determination of FP-hTeloC at pH 4.2 by ^{19}F NMR. Conditions: 300 μM Fp-hTeloC, 50 mM citrate buffer, pH 4.20, 10% D_2O.

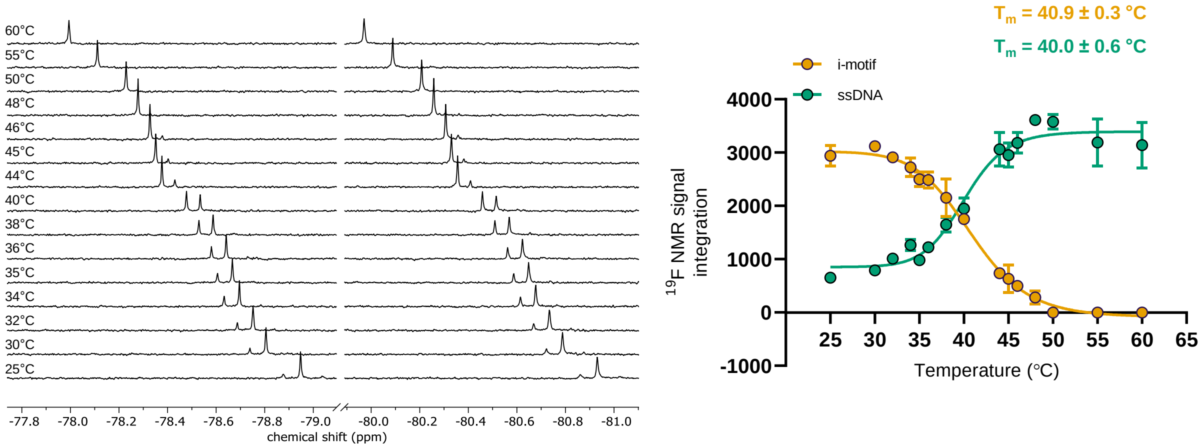

Figure S30. Melting temperature determination of FP-hTeloC at pH 5.5 by ^{19}F NMR. Conditions: 300 μM Fp-hTeloC, 50 mM citrate buffer, pH 5.50, 10% D_2O.

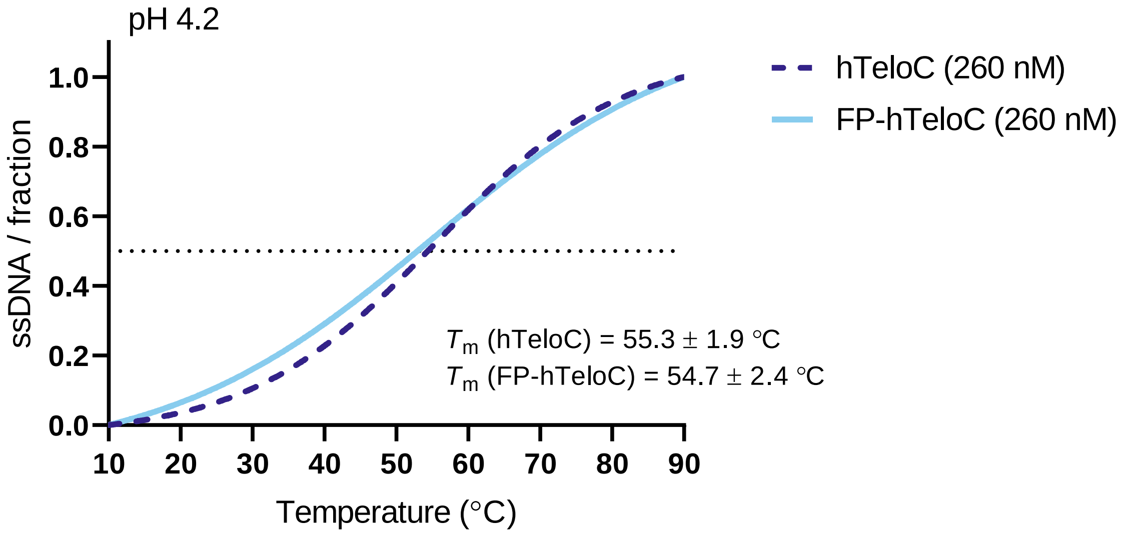

Figure S31. UV/VIS formation monitoring of FP-hTeloC and ON_I in pH 4.2.
Conditions: 4 μM FP-hTeloC, 50 mM citrate buffer, pH 5.50, 10% D_2O; Absorbance at 260 nm was measured.

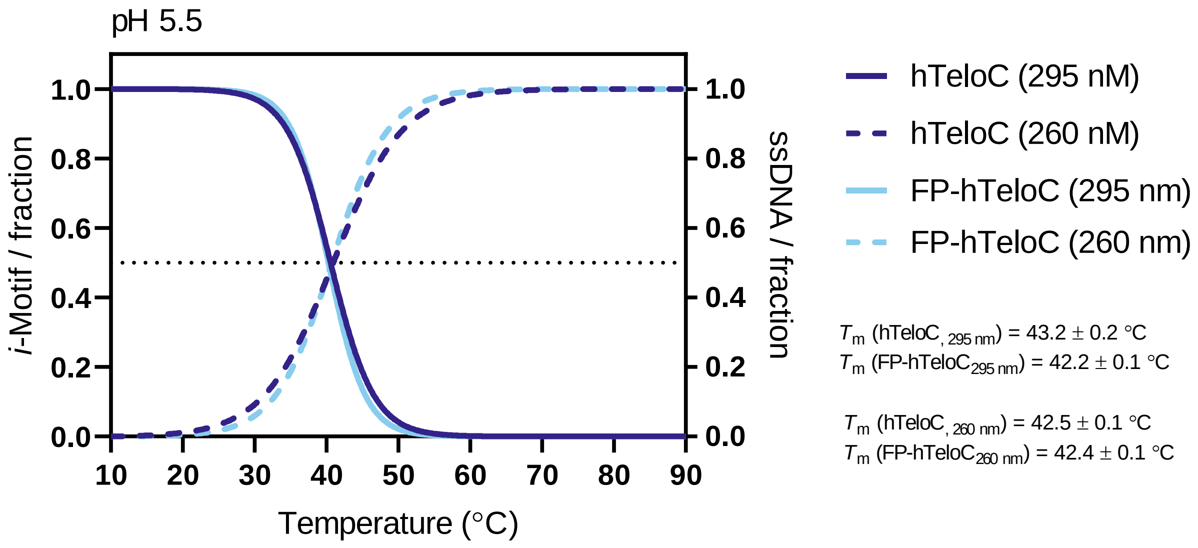

Figure S32. UV/VIS formation monitoring of hTeloC *i*-motif sequences in pH 5.5.
Conditions: 4 μM FP-hTeloC, 50 mM citrate buffer, pH 5.50, 10% D_2O; Absorbance at 260 nm and 295 nm was measured.

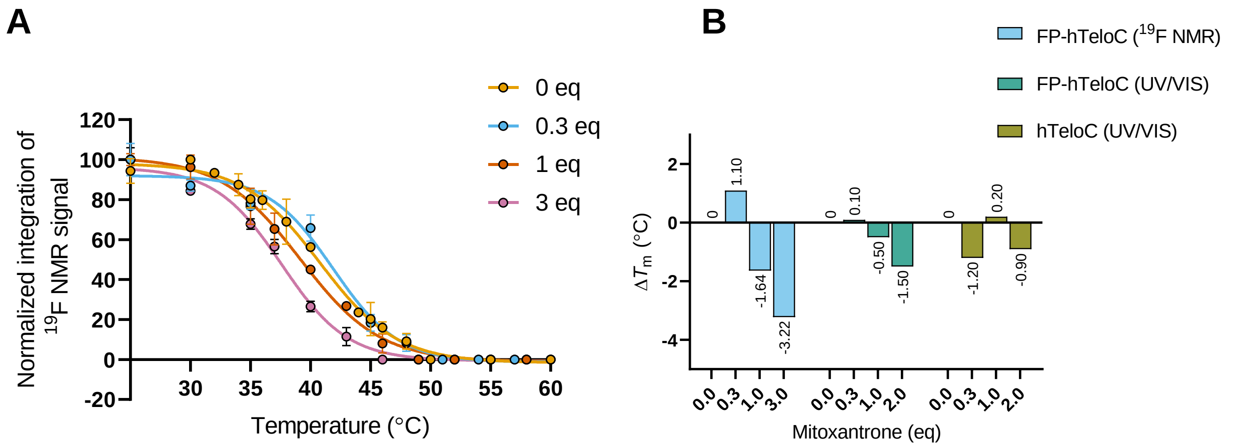

Figure S33. Influence of Mitoxantrone on T_m values of FP-hTeloC and hTeloC.
A) ^{19}F NMR melting profiles with different amount of mitoxantrone; B) Comparison of melting temperature changes. Conditions for ^{19}F NMR experiments: 250 µM FP-hTeloC, 50 mM citrate buffer, pH 5.50, 10% D_2O. UV/VIS experiments: 4 µM FP-hTeloC, 50 mM citrate buffer, pH 5.50, 10% D_2O; $\Delta T_m = T_m$(DNA+ligand) – T_m(DNA). *i*-Motif signal in ^{19}F NMR spectra has been analyzed. Absorbance was measured at 295 nm.

Table S3. Transitions pH (pH_T) and melting temperatures (T_m) values of TCCCCC taken from literature.

5′ TCC CCC 3′			
pH_T	T[a]	method	source
6.1	5 °C	UV/VIS	1
5.8	5 °C	UV/VIS	2
T_m [ºC]	pH[b]	method	source
47.0	4.0	UV/VIS	1
51.5	5.0	UV/VIS	
44.8	4.6	UV/VIS	3
46.0	4.2	UV/VIS	
47.0	5.8	UV/VIS	2

[a] Temperature measurement
[b] pH measurement

Table S4. Melting temperatures (T_m) values of TCC CCC and (TAA CCC)$_4$ determined by UV/VIS.

Sequence (5′→3′)	Bases	Abbreviation	pH 4.2		pH 5.5	
			T_m		T_m	
			260 nm	295 nm	260 nm	295 nm
TCCCCC	6	ON1	50.7 ± 0.4 °C	n.d.*	---	---
TCCCCC	6	FPON1	57.7 ± 0.3 °C	n.d.*	---	---
TCCCCC	6	FPPON1	60.7 ± 0.3 °C	n.d.*	---	---
(TAACCC)$_4$	24	hTeloC	55.3 ± 1.9 °C	n.d.*	42.5 ± 0.1 °C	43.2 ± 0.2 °C
(TAACCC)$_4$	24	FP-hTeloC	54.7 ± 2.4 °C	n.d.*	42.4 ± 0.1 °C	42.2 ± 0.1 °C

* no hyperchromic (increase of absorbance) or hypochromic (decrease of absorbance) effect observed

Table S5. Transitions pH (pH$_T$) and melting temperatures (T_m) values of hTeloC taken from literature.

5′ (TAA CCC)$_4$ 3′			
pH$_T$	T[a]	method	source
5.1	37 °C	CD	4
6.5	20 °C	CD	5
T_m [ºC]	pH[b]	method	source
52.8	4.3	CD	6
40.6	5.7	CD	
43.0	5.5	UV/VIS	5

[a] Temperature of measurement
[b] pH of measurement

References

1. Abou Assi, H., Harkness, R.W., Martin-Pintado, N., Wilds, C.J., Campos-Olivas, R., Mittermaier, A.K., Gonzalez, C. and Damha, M.J. (2016) Stabilization of i-motif structures by 2′-beta-fluorination of DNA. *Nucleic Acids Res.*, **44**, 4998-5009.
2. Tsvetkov, V.B., Zatsepin, T.S., Belyaev, E.S., Kostyukevich, Y.I., Shpakovski, G.V., Podgorsky, V.V., Pozmogova, G.E., Varizhuk, A.M. and Aralov, A.V. (2018) i-Clamp phenoxazine for the fine tuning of DNA i-motif stability. *Nucleic Acids Res.*, **46**, 2751-2764.
3. Fenna, C.P., Wilkinson, V.J., Arnold, J.R.P., Cosstick, R. and Fisher, J. (2008) The effect of 2′-fluorine substitutions on DNA i-motif conformation and stability. *ChemComm*, 3567-3569.
4. Rogers, R.A., Fleming, A.M. and Burrows, C.J. (2018) Unusual isothermal hysteresis in DNA i-motif pH transitions: a study of the RAD17 promoter sequence. *Biophys J.*, **114**, 1804-1815.
5. Wright, E.P., Huppert, J.L. and Waller, Z.A.E. (2017) Identification of multiple genomic DNA sequences which form i-motif structures at neutral pH. *Nucleic Acids Res.*, **45**, 2951-2959.
6. Pagano, A., Iaccarino, N., Abdelhamid, M.A.S., Brancaccio, D., Garzarella, E.U., Di Porzio, A., Novellino, E., Waller, Z.A.E., Pagano, B., Amato, J. *et al.* (2018) Common G-quadruplex binding agents found to interact with i-motif-forming DNA: unexpected multi-target-directed compounds. *Front. Chem.*, **6**.
