# Supplementary material for: 5′-fluoro(di)phosphate-labeled oligonucleotides are versatile molecular probes for studying nucleic acid secondary structure and interactions by 19F NMR
Source: Nucleic Acids Res. 2020 Jun 9;48(15):8209–24. doi: 10.1093/nar/gkaa470 (PMC7470941; doi:10.1093/nar/gkaa470)

Supplementary Information 2

5′-fluoro(di)phosphate-labeled oligonucleotides are versatile molecular probes for studying nucleic acid secondary structure and interactions by ^{19}F NMR

Marek R. Baranowski[1], Marcin Warminski[1], Jacek Jemielity[2] and Joanna Kowalska[1,*]

[1] Division of Biophysics, Institute of Experimental Physics, Faculty of Physics, University of Warsaw, Ludwika Pasteura 5, 02-093 Warsaw, Poland
[2] Centre of New Technologies, University of Warsaw, Stefana Banacha 2c, 02-097 Warsaw, Poland

This file gathers reverse phase high-pressure liquid chromatography (RP HPLC) profiles, high-resolution mass spectrometry profiles (HRMS) and ^{19}F NMR spectra of all synthesized oligonucleotides.

Table of contents

Fluorophosphorylated oligonucleotides 4
FPON1 4
FPPON1 5
FPPON2 6
FPON3 7
FPPON3 8
FPPON4 9
FPPON5 10
FPPON6 11
FPPON7 12
FPPON8 13
FPPON9 14
FPPON10 15
FPPON11 16
FPPON12 17
FPPON13 18
FPPON14 19
FPPON15 20
FP-hTeloC 21
Unmodified oligonucleotides 22
ON1 22

hTeloC 23
ON3 24
ON4 25
ON5 26
ON9 27
ON11 28
PON11 29
ON20 30
ON21 31
ON22 32
ON23 33
ON24 34
ON25 35

Fluorophosphorylated oligonucleotides

FPON1

Sequence Fp TCC CCC

HRMS (calc. m/z – 883.64206, found m/z – 883.63531)

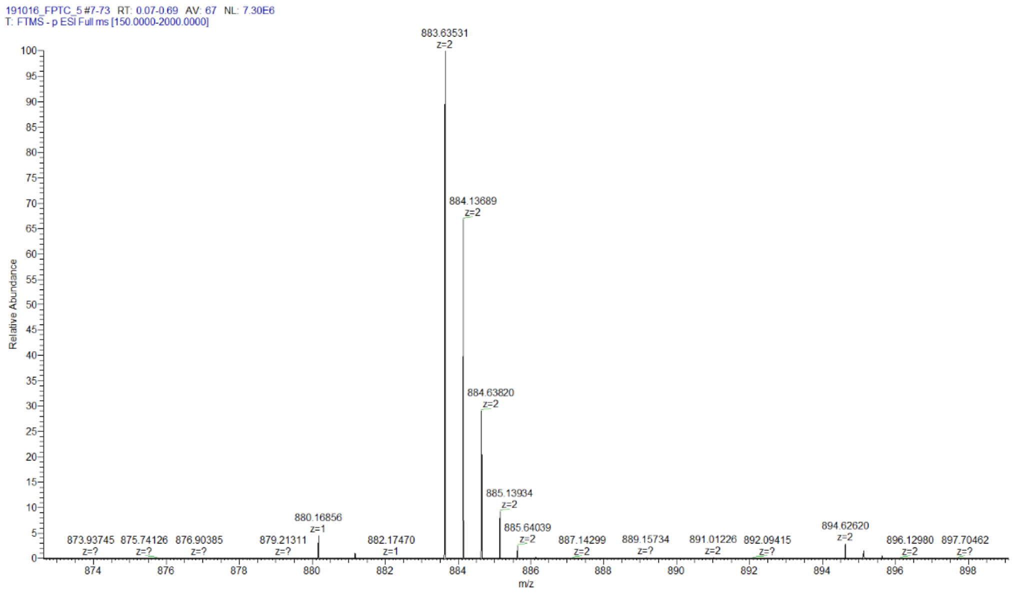

RP HPLC (purified product)

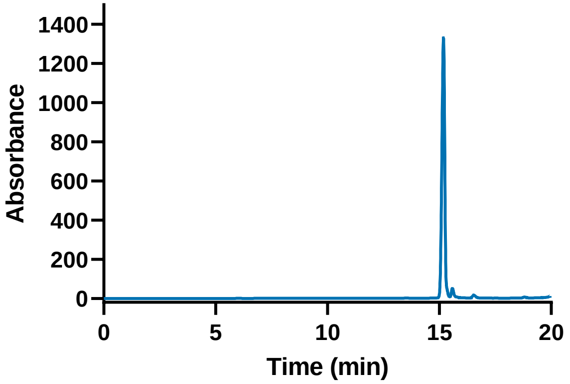

^{19}F NMR (471 MHz, 50 mM sodium citrate buffer pH 4.20, 10% D_2O): δ -79.86 (1 F, d, *J* = 936.2 Hz)

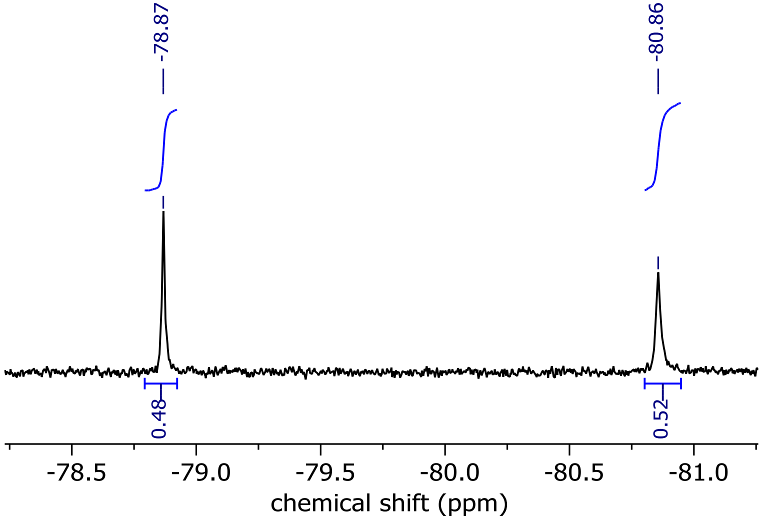

FPPON1

Sequence Fpp TCC CCC

HRMS (calc. m/z – 923.61795, found m/z – 923.61885)

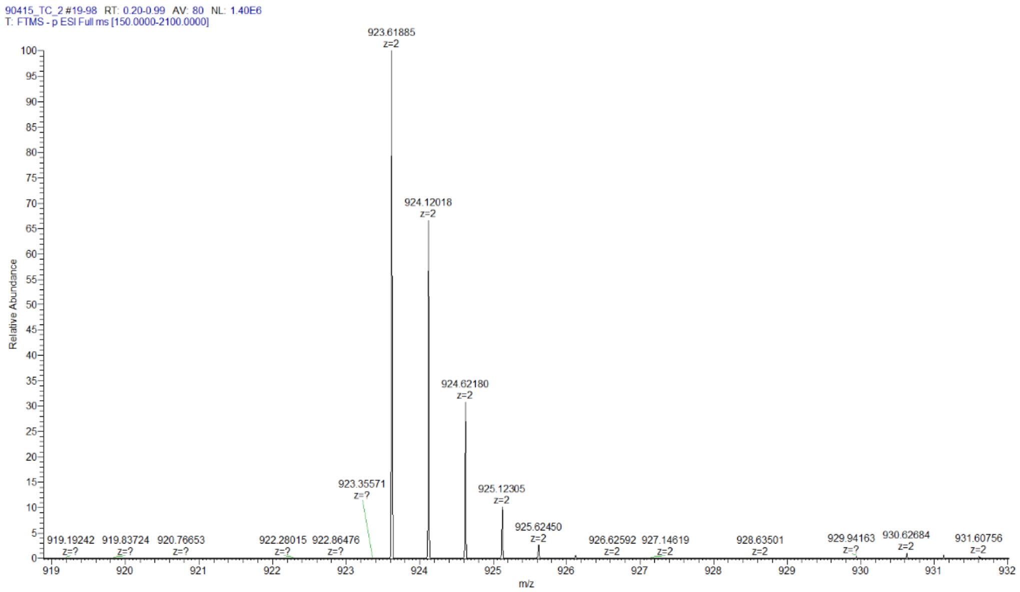

RP HPLC (purified product)

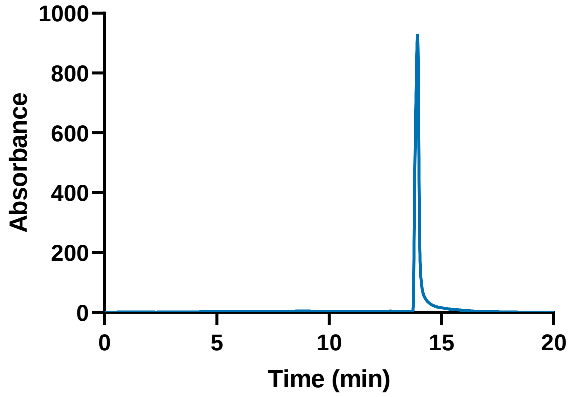

^{19}F NMR (471 MHz, 50 mM sodium citrate buffer pH 4.20, 10% D_2O): δ -73.06 (1 F, d, *J* = 934.6 Hz)

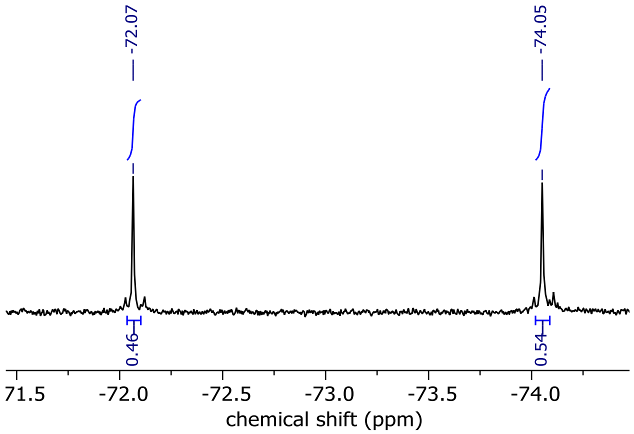

FPPON2

Sequence Fpp GTC AAT G

HRMS (calc. m/z – 1139.65835, found m/z – 1139.65965)

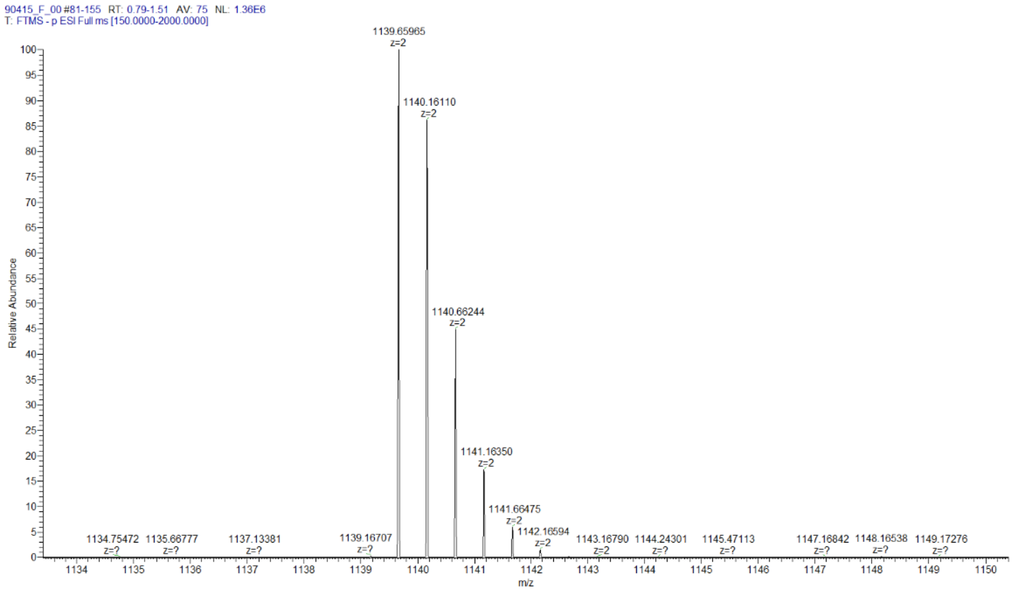

RP HPLC (purified product)

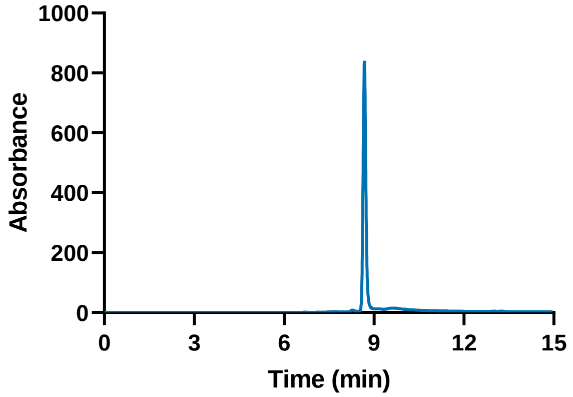

^{19}F NMR (471 MHz, D_2O): δ -73.34 (1 F, d, J = 933.7 Hz)

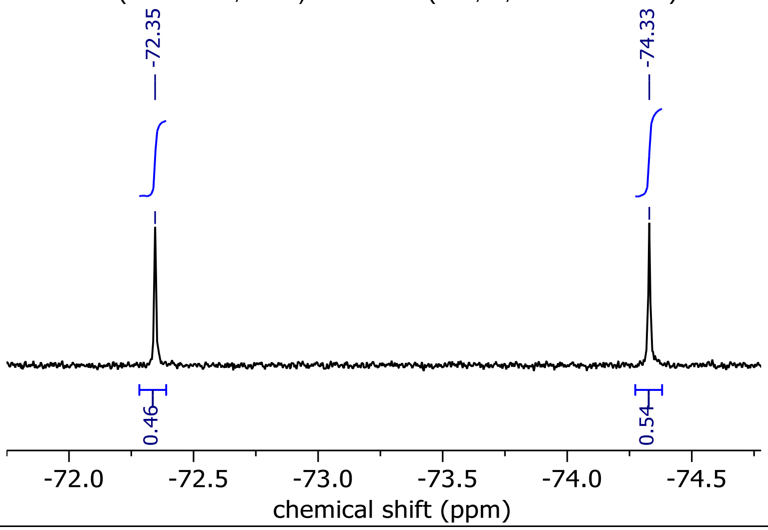

FPON3

Sequence Fp AGA CAT TGA C

HRMS (calc. m/z - 1037.83489 , found m/z – 1037.83648)

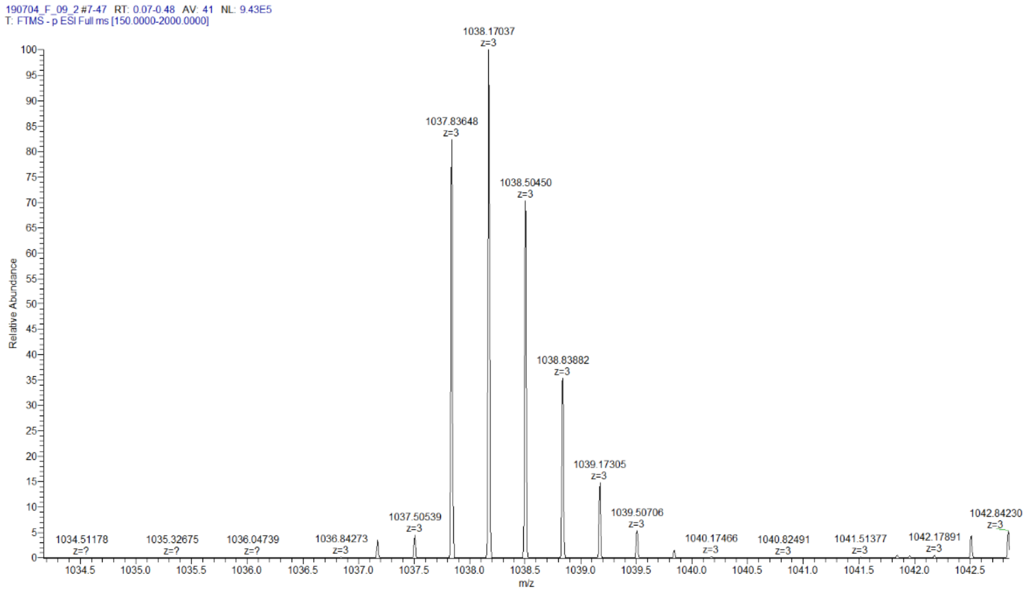

RP HPLC (purified product)

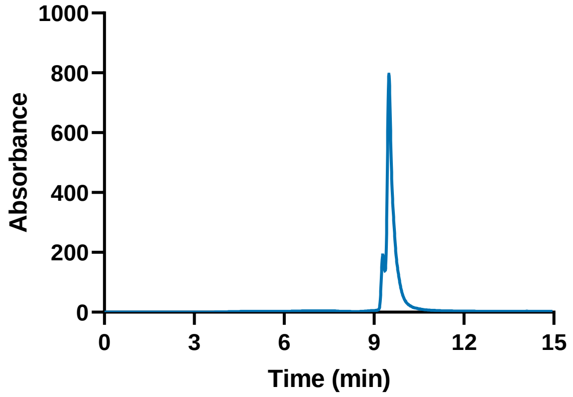

^{19}F NMR (471 MHz, D_2O): δ -80.18 (1 F, d, J = 934.6 Hz)

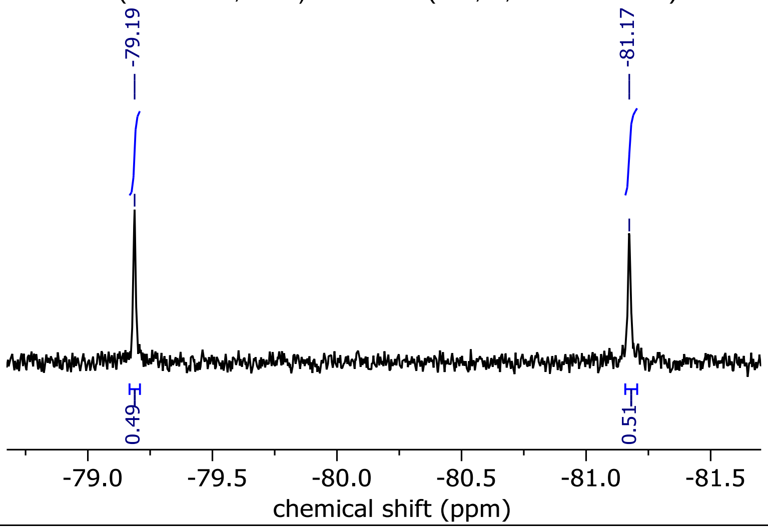

FPPON3

Sequence Fpp AGA CAT TGA C

HRMS (calc. m/z - 1064.49033, found m/z – 1064.49194)

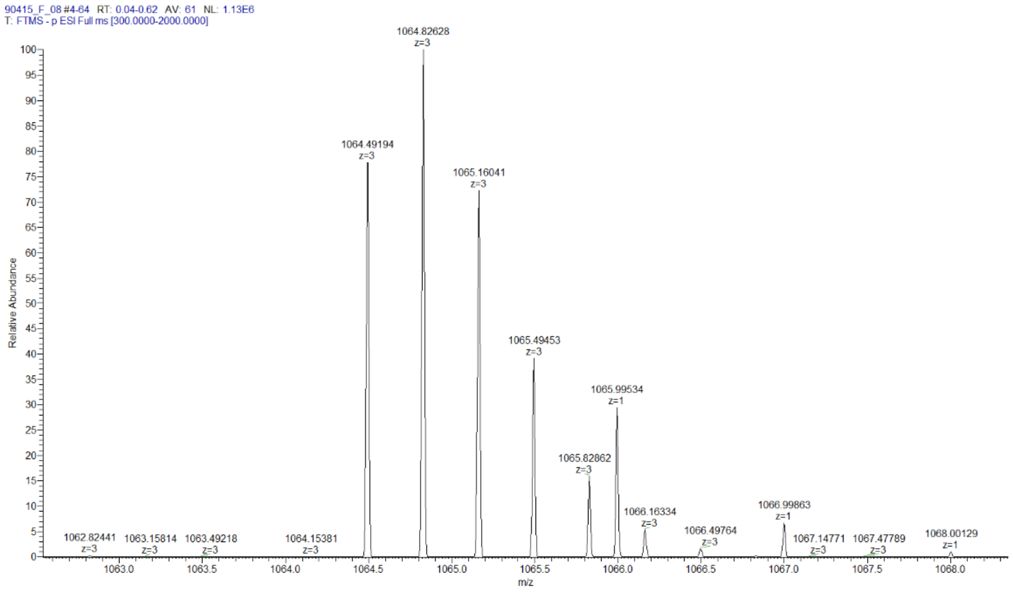

RP HPLC (purified product)

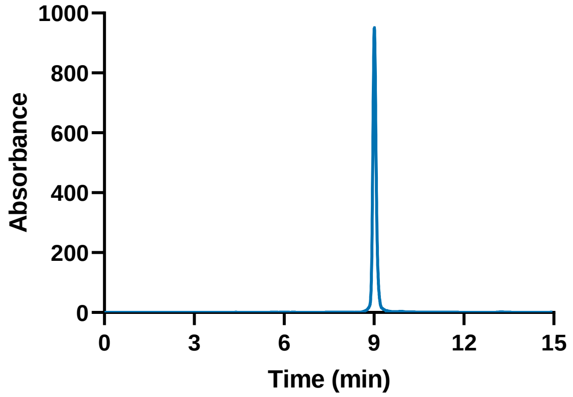

^{19}F NMR (471 MHz, D_2O): δ -73.49 (1 F, d, *J* = 934.5 Hz)

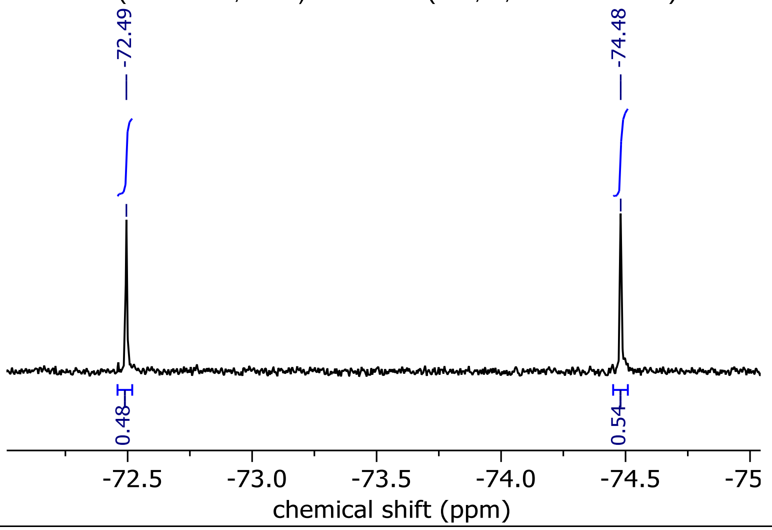

FPPON4

Sequence Fpp TGA CAT TGA C

HRMS (calc. m/z - 1061.48648 , found m/z – 1061.48788)

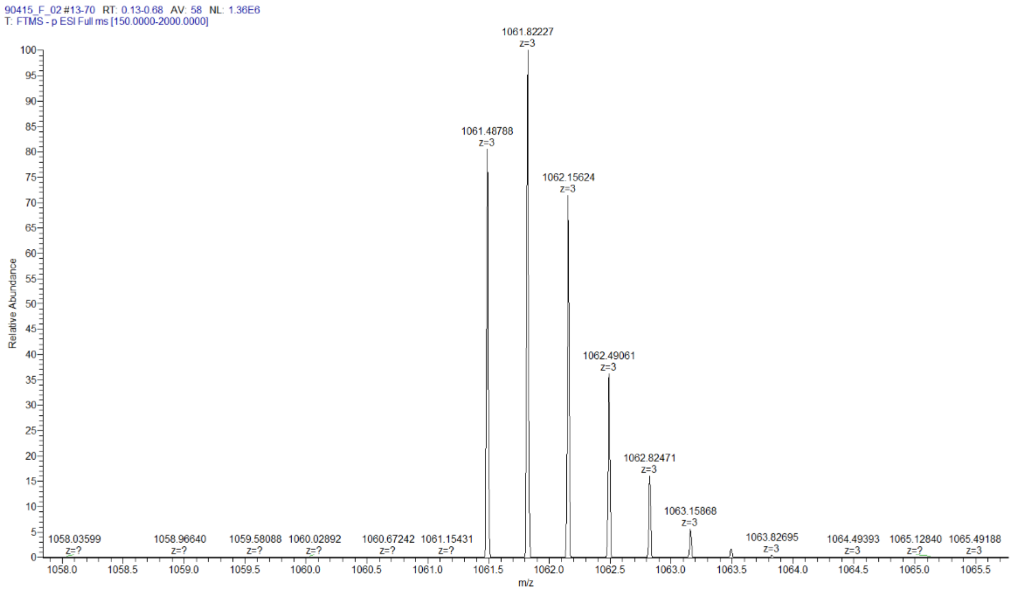

RP HPLC (purified product)

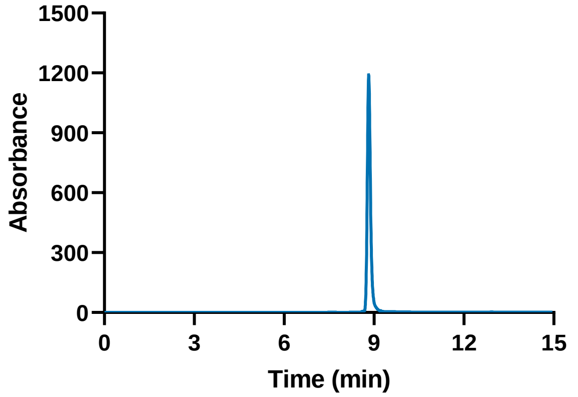

^{19}F NMR (471 MHz, D_2O): δ -73.33 (1 F, d, *J* = 933.7 Hz)

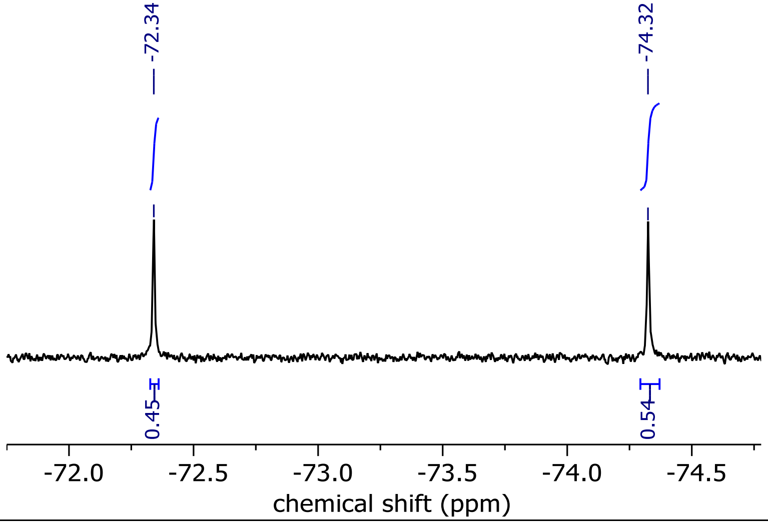

FPPON5

Sequence Fpp GTC AAT GTC C

HRMS (calc. m/z - 1053.48273, found m/z – 1053.48426)

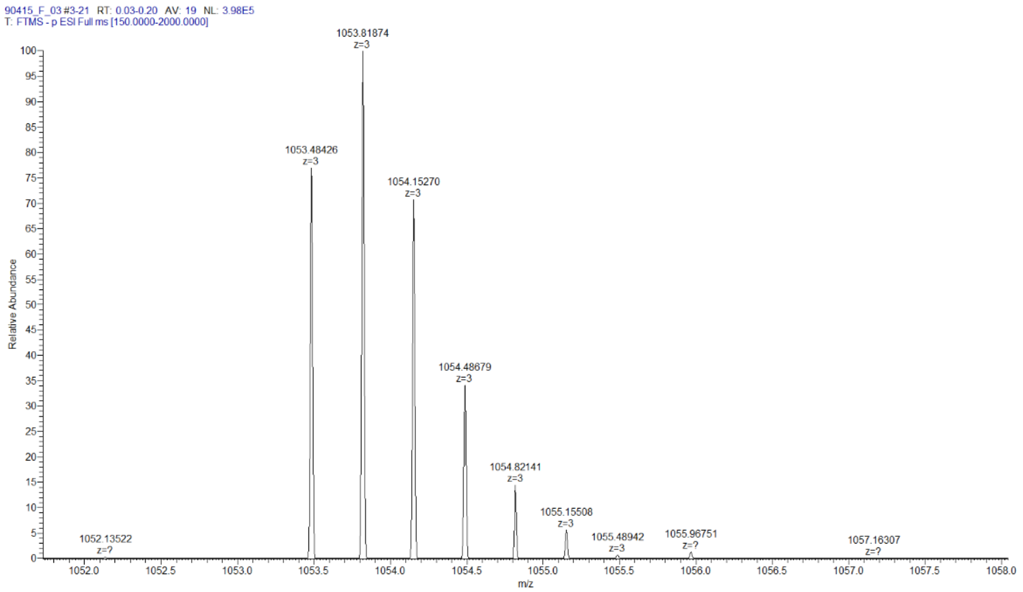

RP HPLC (purified product)

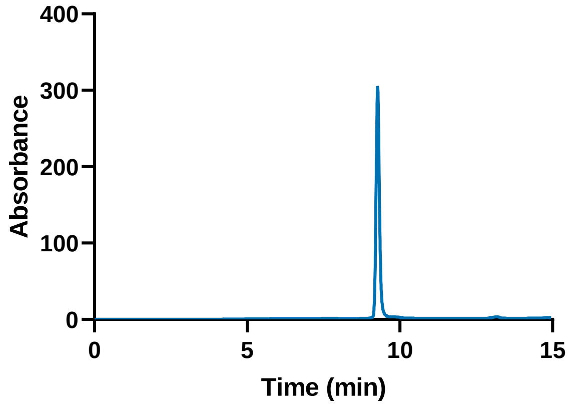

^{19}F NMR (471 MHz, D_2O): δ -73.51 (1 F, d, J = 934.2 Hz)

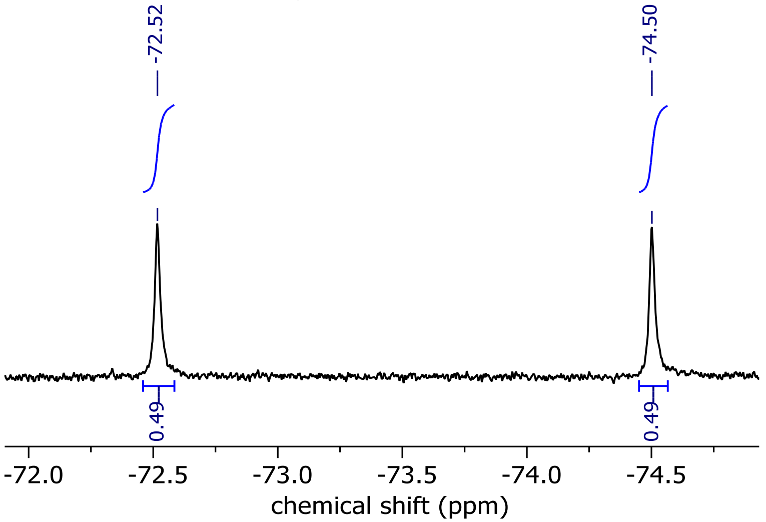

FPPON6

Sequence Fpp GGA CAT TGA C

HRMS (calc. m/z - 1069.82197, found m/z – 1069.82293)

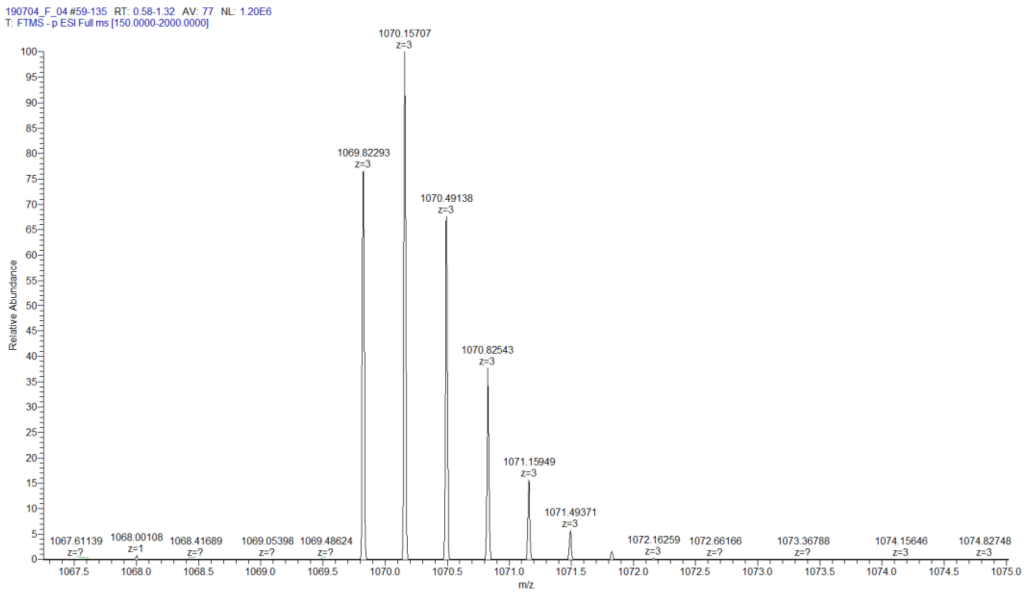

RP HPLC (purified product)

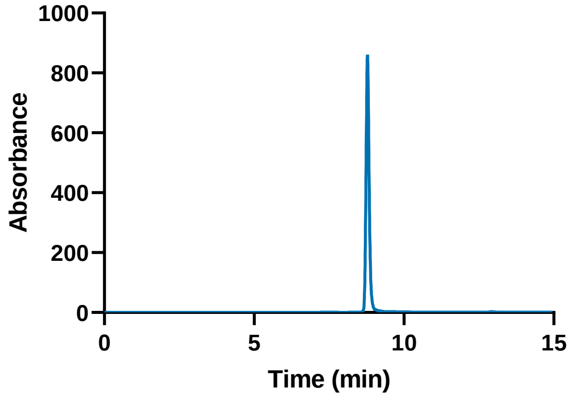

^{19}F NMR (471 MHz, D_2O): δ -73.45 (1 F, d, J = 934.0 Hz)

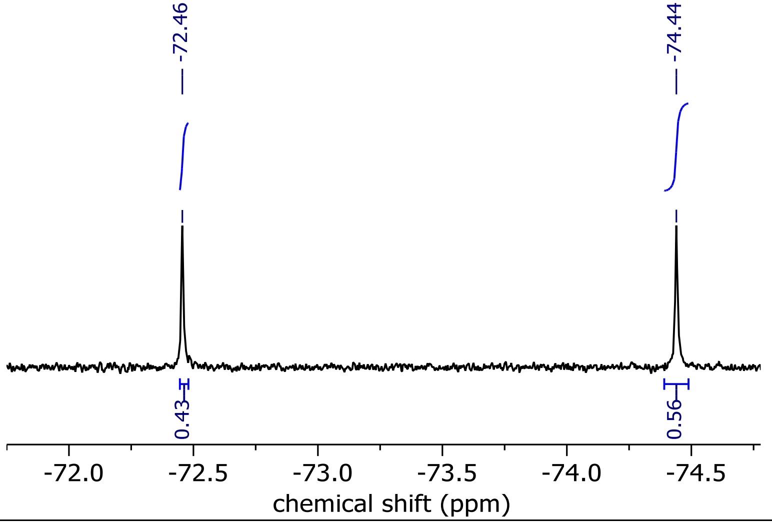

FPPON7

Sequence Fpp ATC AAT GTC G

HRMS (calc. m/z - 1061.48648, found m/z – 1061.48737)

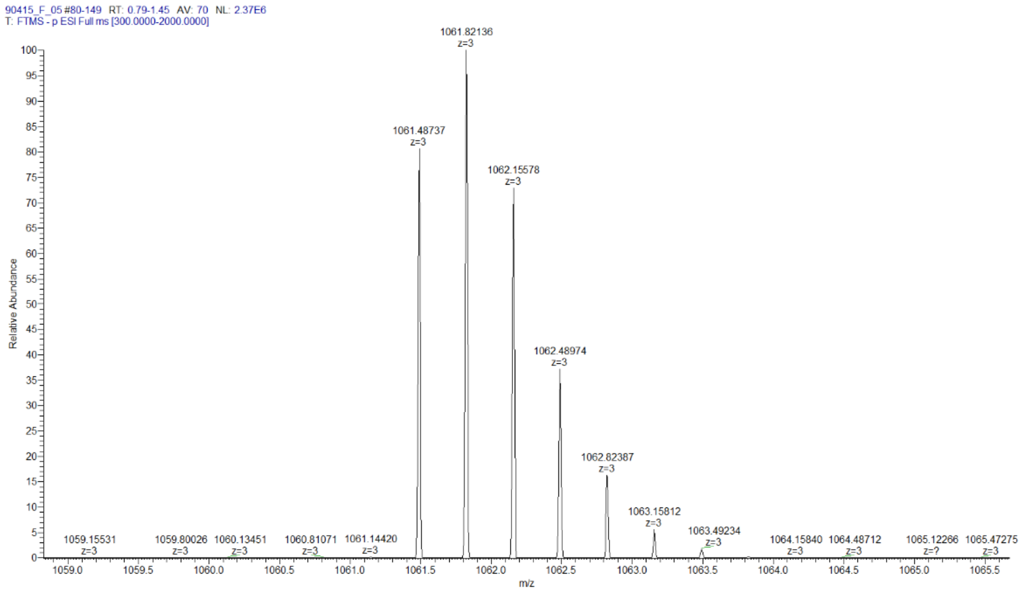

RP HPLC (purified product)

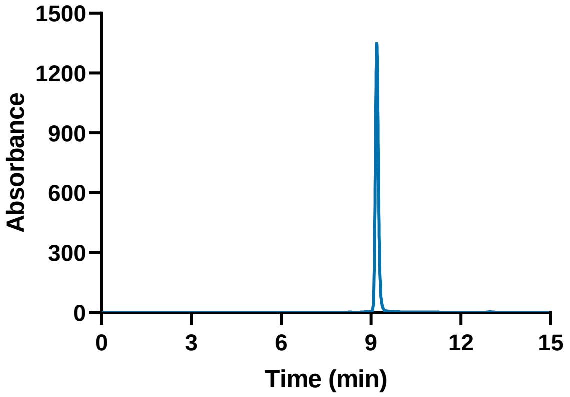

^{19}F NMR (471 MHz, D_2O): δ -73.30 (1 F, d, $J_{F\text{-}P}$ = 933.9 Hz)

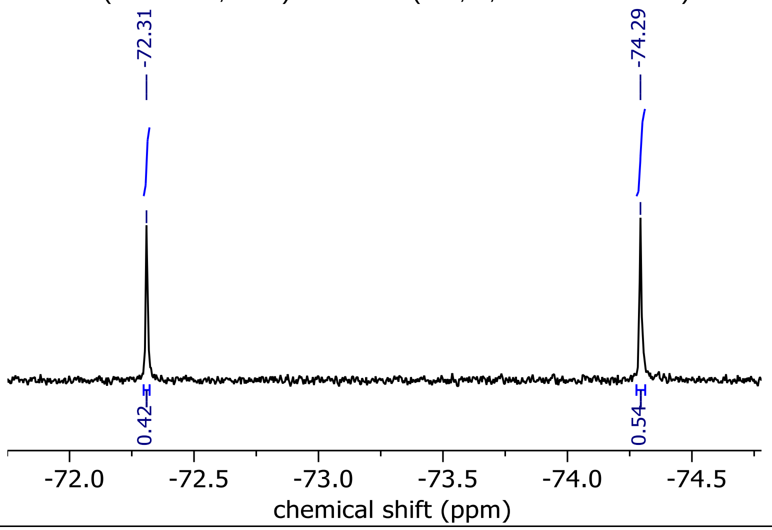

FPPON8

Sequence Fpp CGA CAT TGA T

HRMS (calc. m/z - 1061.48648, found m/z – 1061.48751)

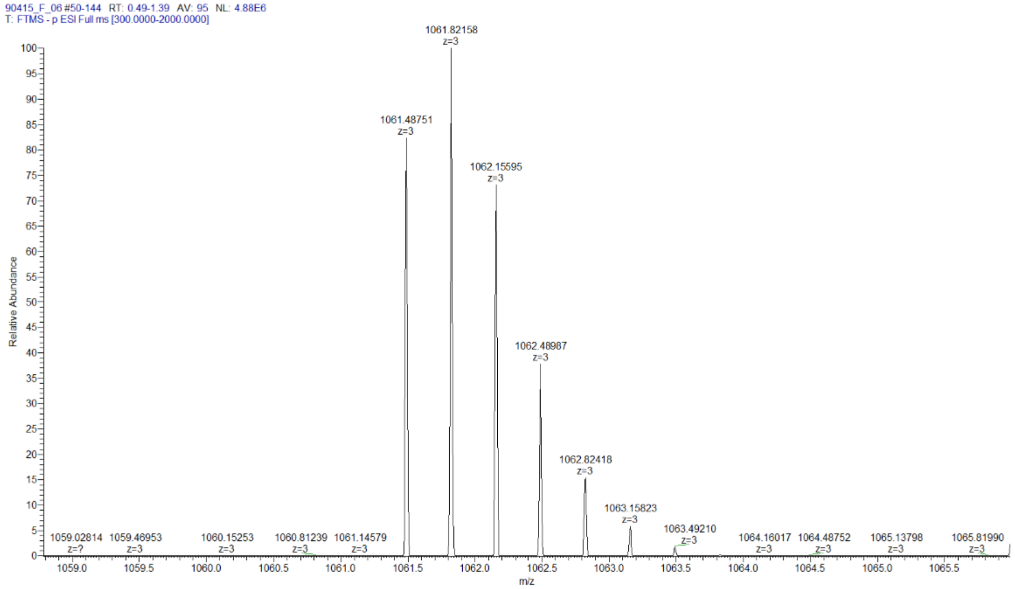

RP HPLC (purified product)

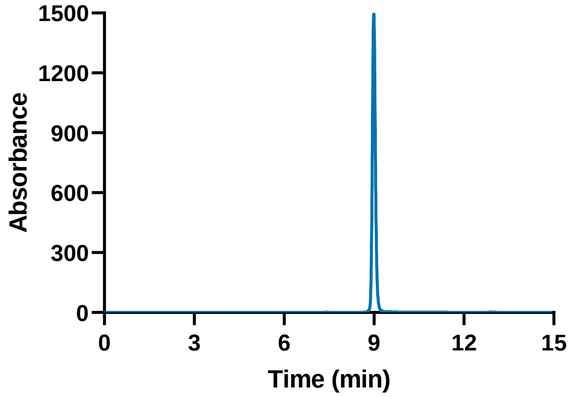

^{19}F NMR (471 MHz, D_2O): δ -73.46 (1 F, d, J = 934.5 Hz)

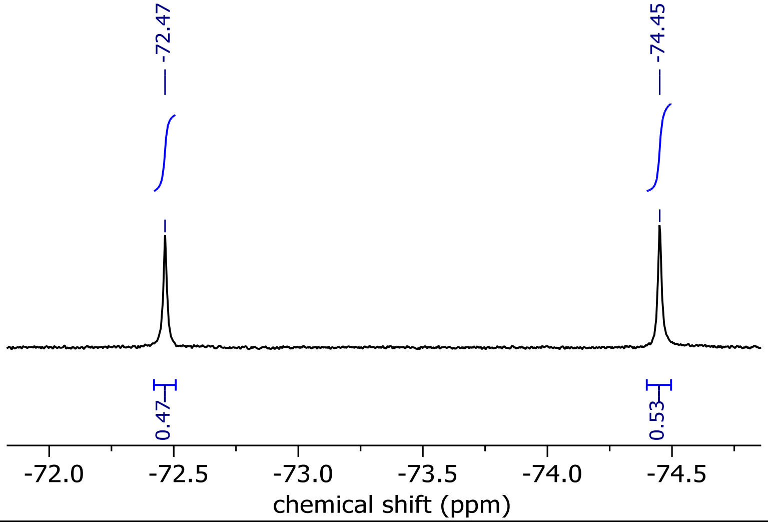

FPPON9

Sequence Fpp GTC AAT GTC T

HRMS (calc. m/z - 1058.48262, found m/z -1058.48364)

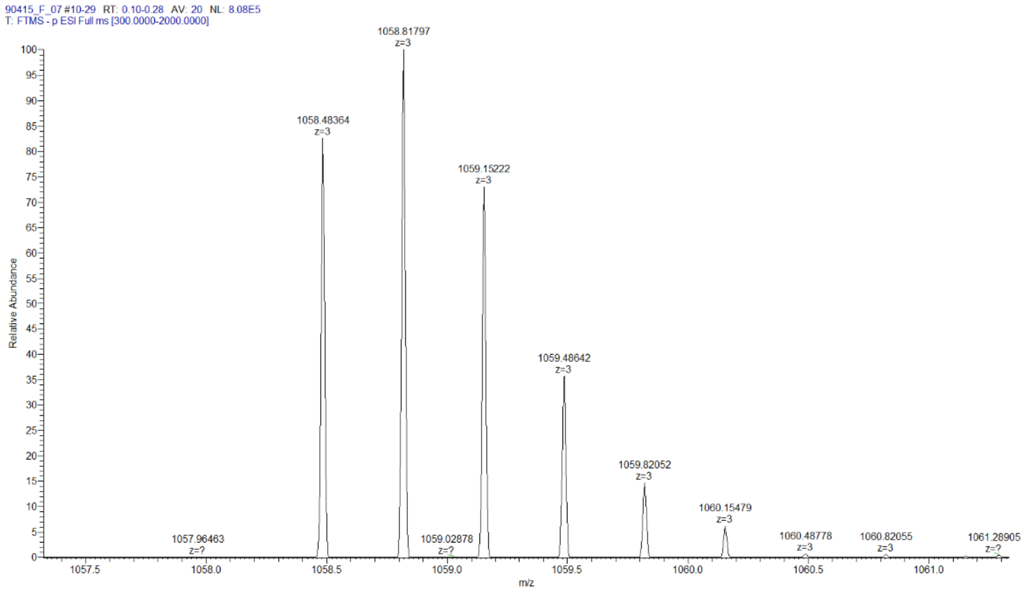

RP HPLC (purified product)

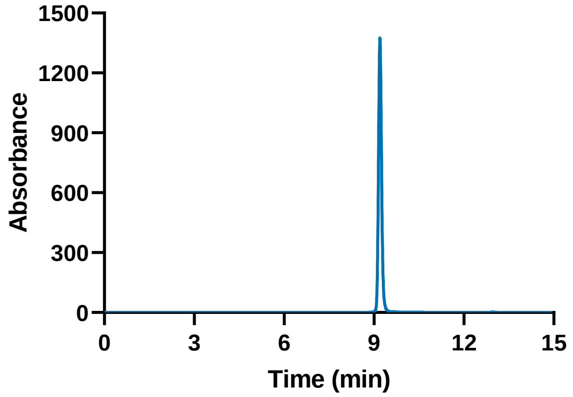

^{19}F NMR (471 MHz, D_2O): δ -73.54 (1 F, d, *J* = 934.3 Hz)

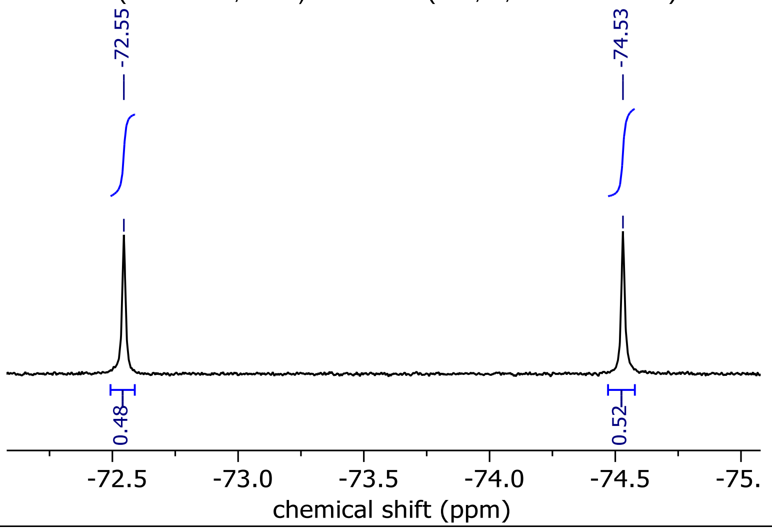

FPPON10

Sequence Fpp GTC AAT GTC AGC G

HRMS (calc. m/z - 1377.20362, found m/z – 1377.20421)

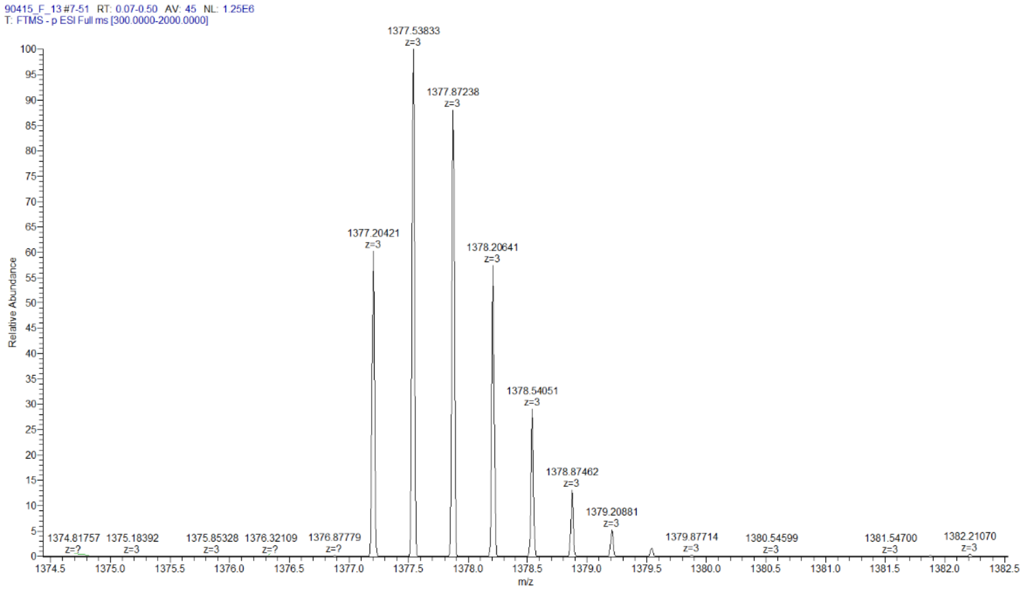

RP HPLC (purified product)

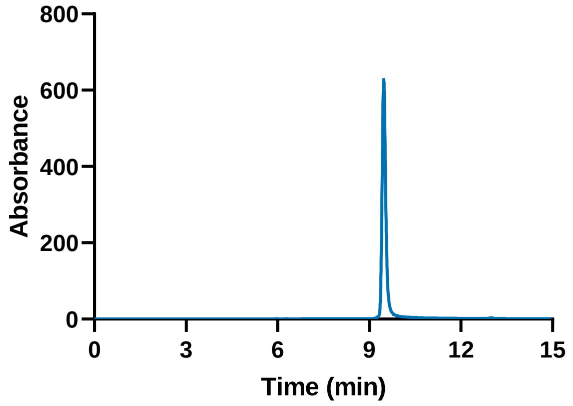

^{19}F NMR (471 MHz, D_2O): δ -73.52 (1 F, d, *J* = 934.2 Hz)

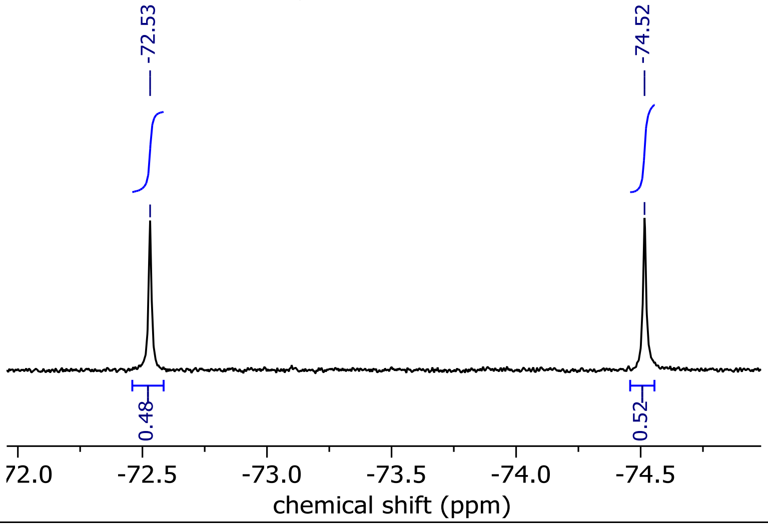

FPPON11

Sequence Fpp GGT TGG TGT GGT TGG

HRMS
(calc. m/z - 1220.42309 , found m/z – 1220.42932)

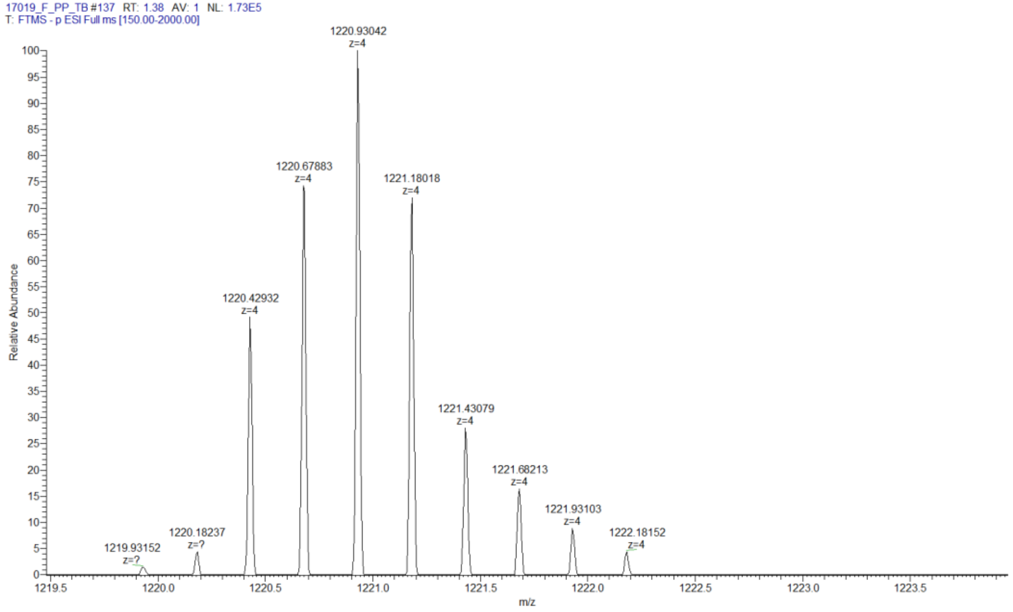

RP HPLC (purified product)

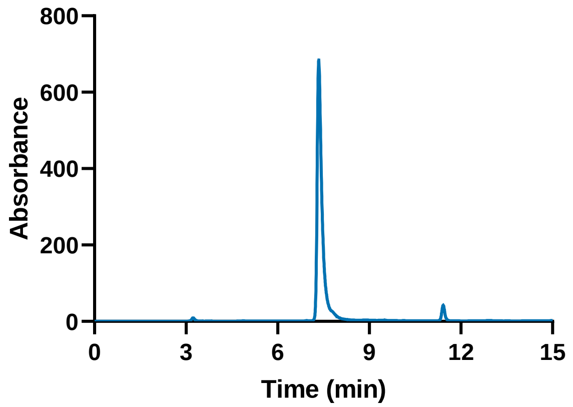

^{19}F NMR (471 MHz, D_2O): δ -73.26 (1 F, d, *J* = 932.3 Hz)

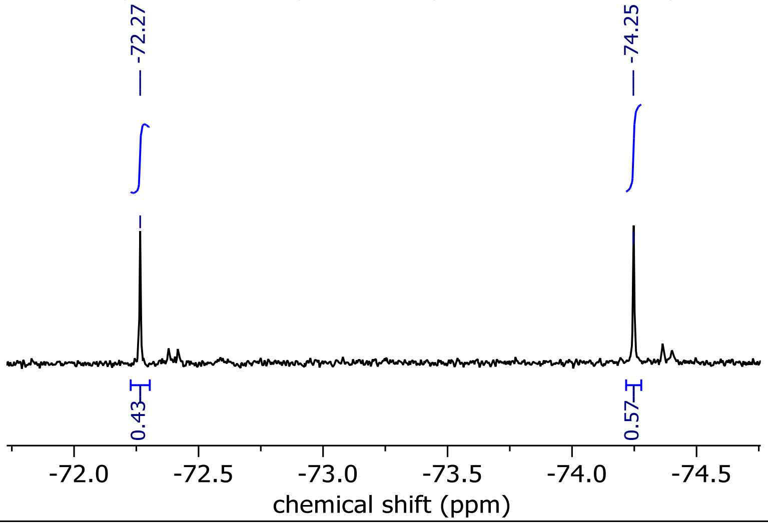

FPPON12

Sequence Fpp GGA TAC TTT TGT ATC C

HRMS (calc. m/4z - 1254.43380, found m/4z – 1254.43400)

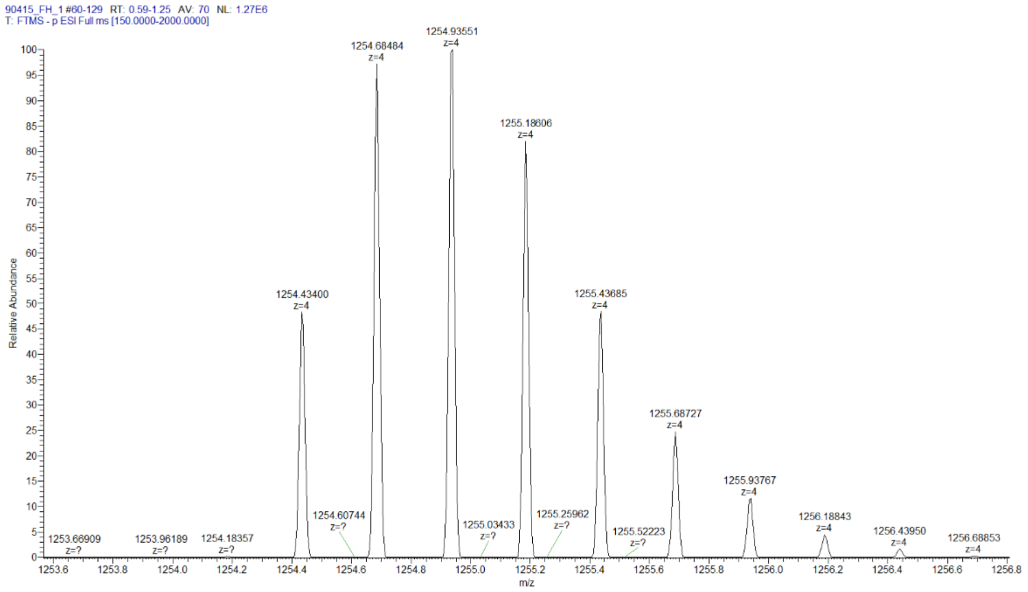

RP HPLC (purified product)

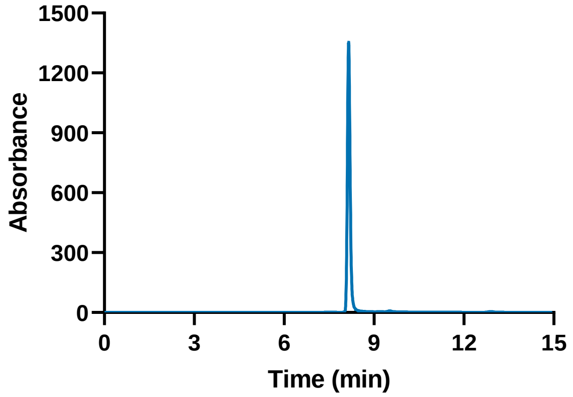

^{19}F NMR (471 MHz, D_2O): δ -73.43 (1 F, d, J = 934.4 Hz)

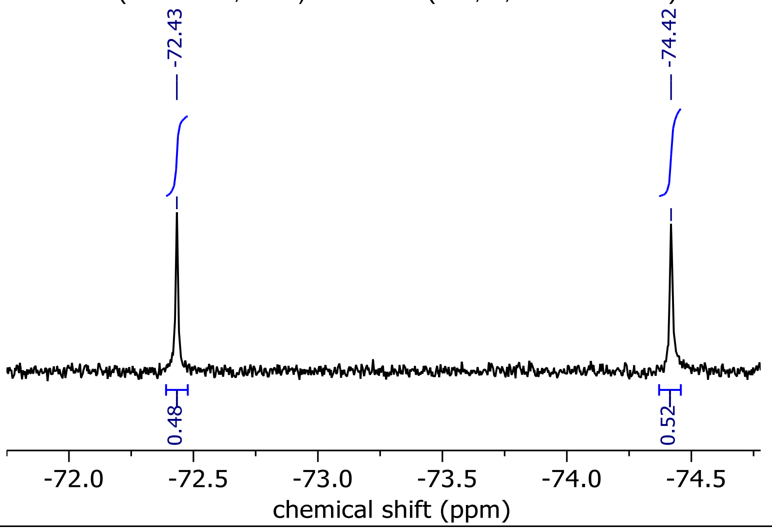

FPPON13

Sequence Fpp GGA TAT TTT TAT ATC C

HRMS (calc. m/4z - 1254.18499, found m/4z – 1254.18523)

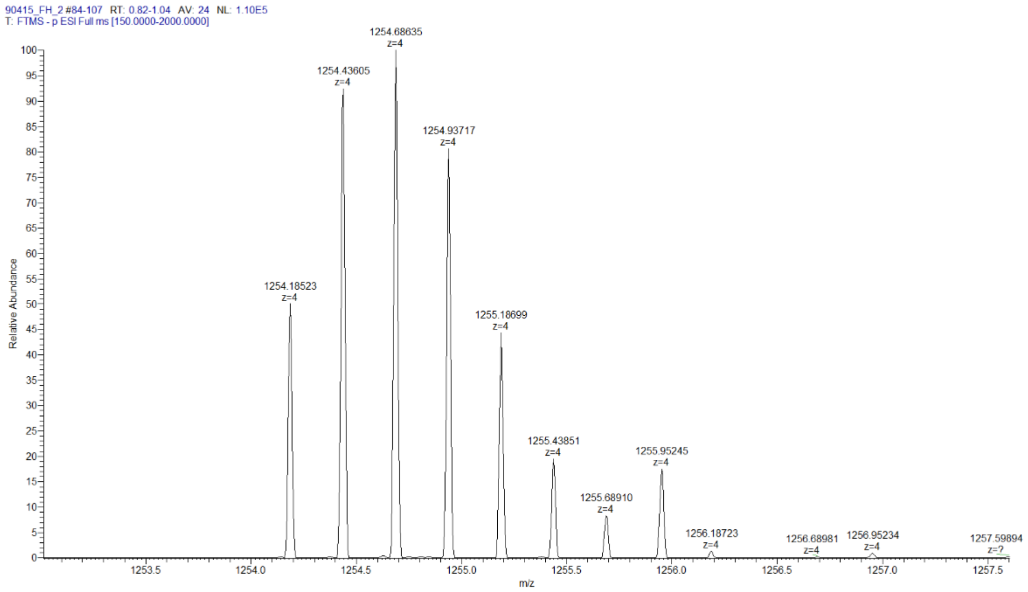

RP HPLC (purified product)

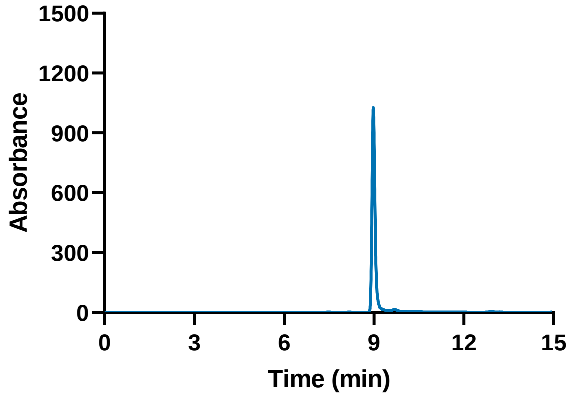

^{19}F NMR (471 MHz, D_2O): δ -73.64 (1 F, d, *J* = 934.7 Hz)

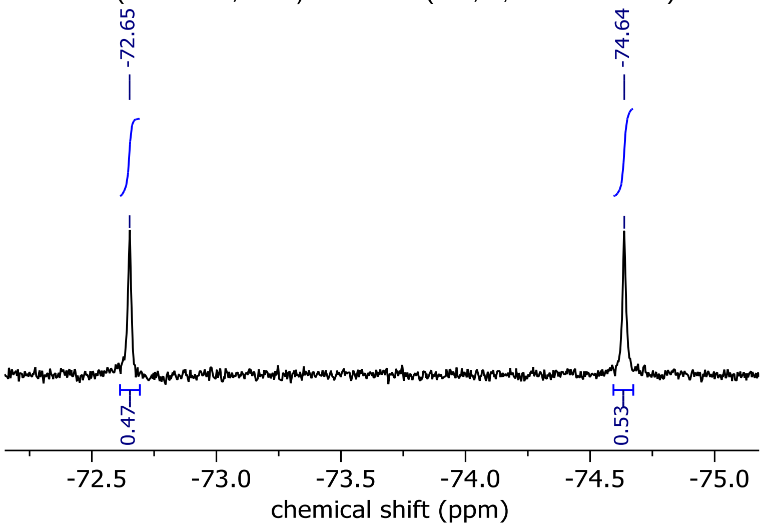

FPPON14

Sequence Fpp GTC AAT GTC AGC GAT A

HRMS (calc. m/3z – 1687.25737, found m/3z – 1687.25876)

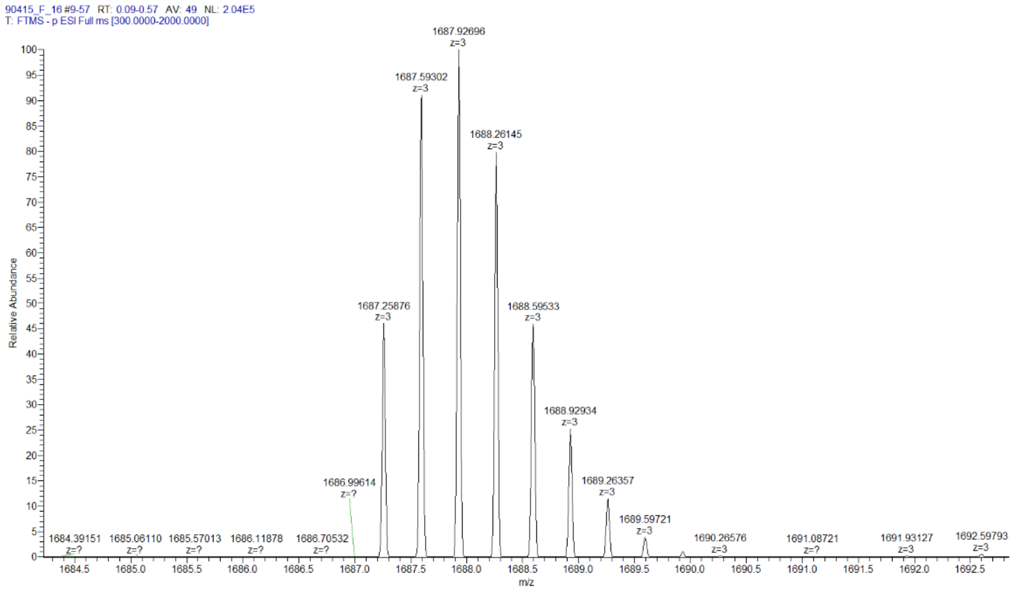

RP HPLC (purified product)

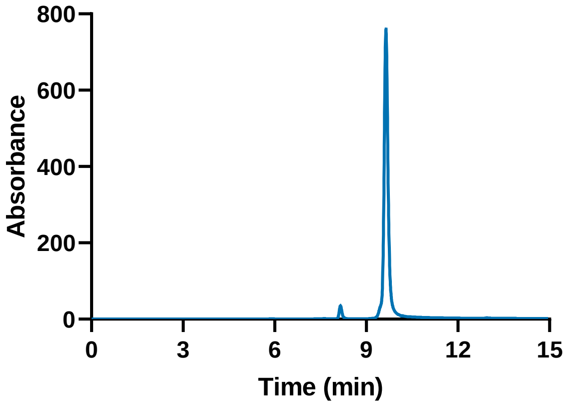

^{19}F NMR (471 MHz, D_2O): δ -73.33 (1 F, d, *J* = 933.9 Hz)

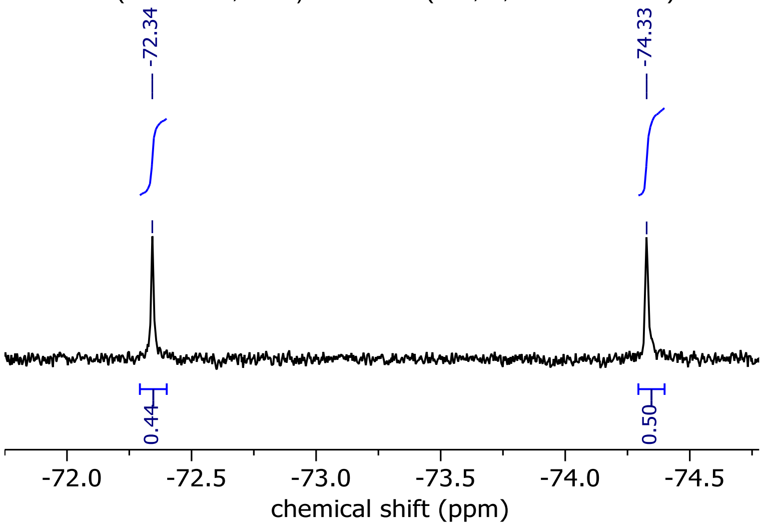

FPPON15

Sequence Fpp CCT GGG GGA GTA TTG CGG AGG AAG G

HRMS (calc. m/z - 1604.24604, found m/5z – 1604.24765)

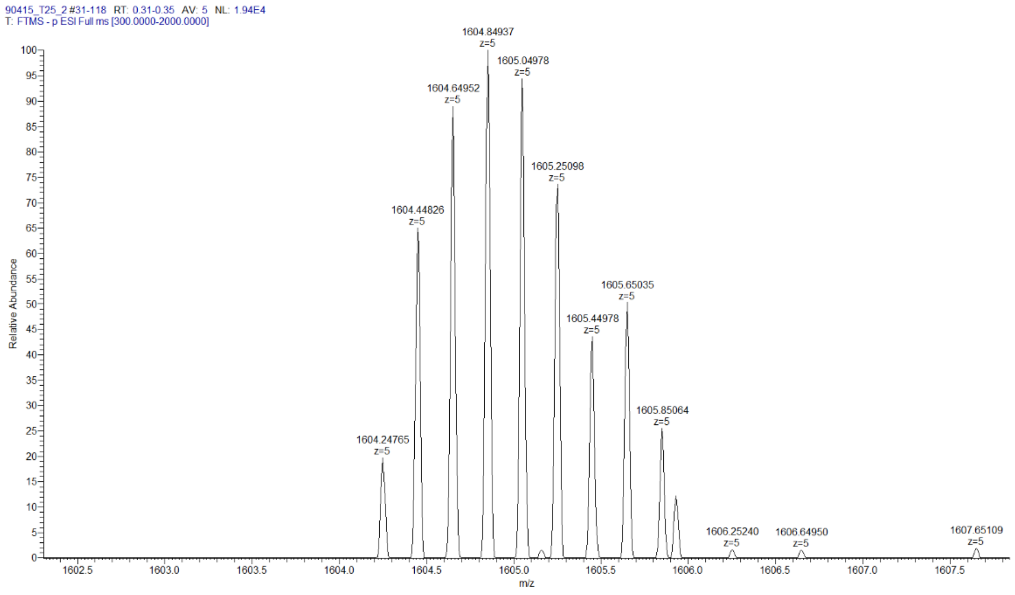

RP HPLC (purified product)

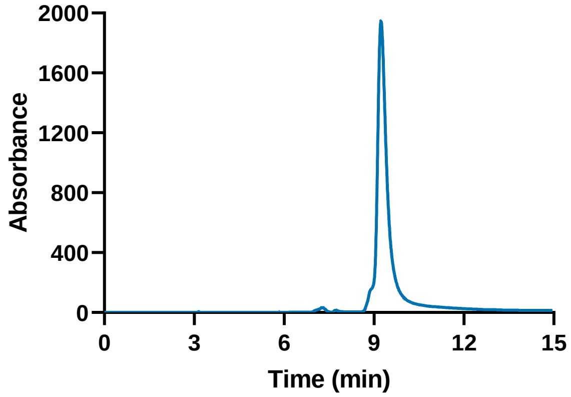

^{19}F NMR (471 MHz, D_2O): δ -73.39 (1 F, d, *J* = 935.7 Hz)

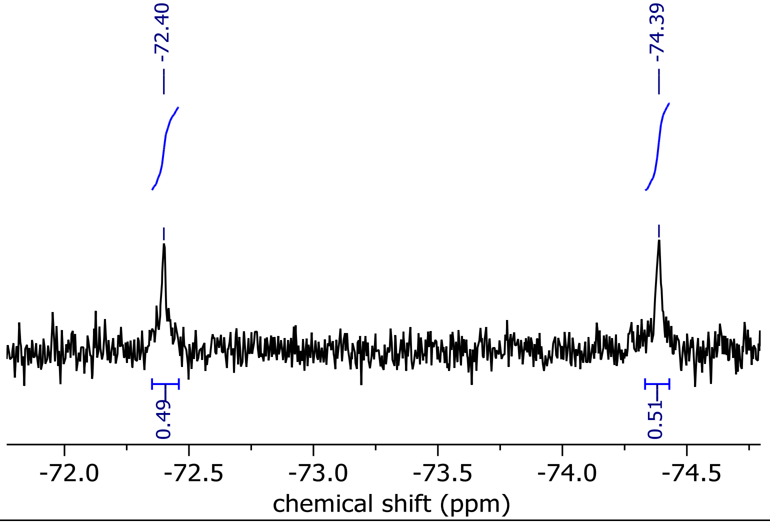

FP-hTeloC

Sequence Fp TAA CCC TAA CCC TAA CCC TAA CCC

HRMS (calc. m/4z – 1801.301921, found m/4z – 1801.29517)

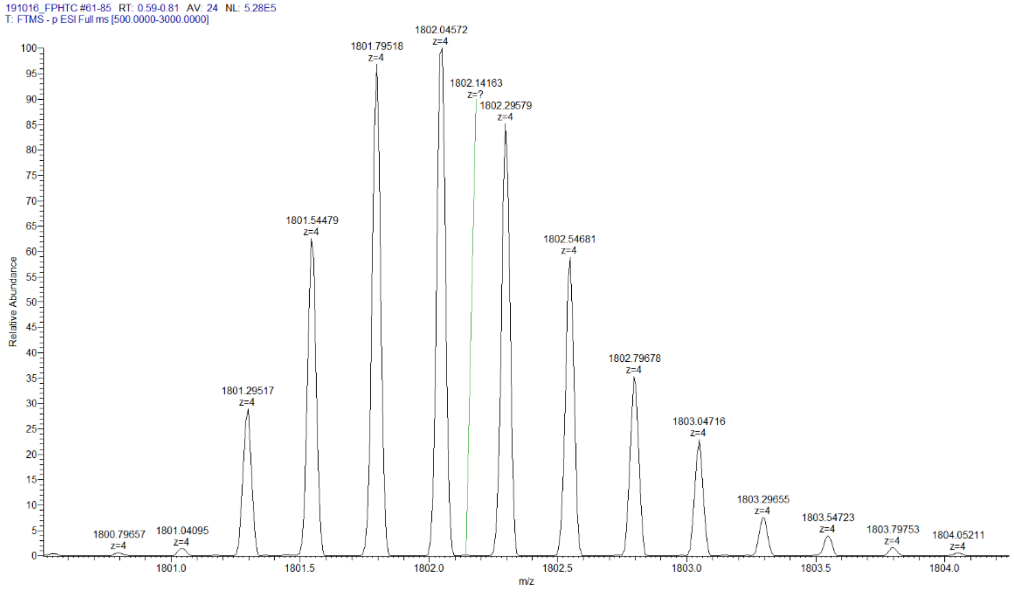

RP HPLC (purified product)

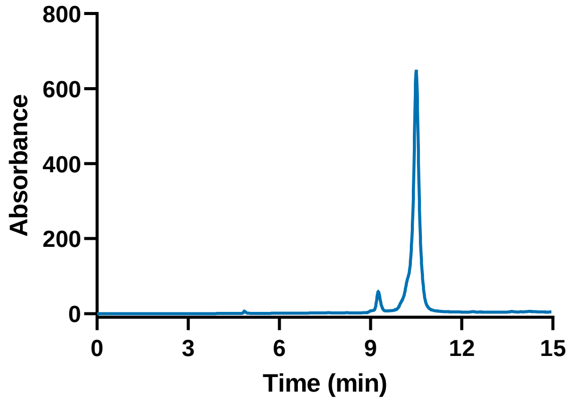

^{19}F NMR (471 MHz, 50 mM sodium citrate buffer pH 4.20, 10% D_2O): δ -79.95 (d, J = 933.6 Hz)

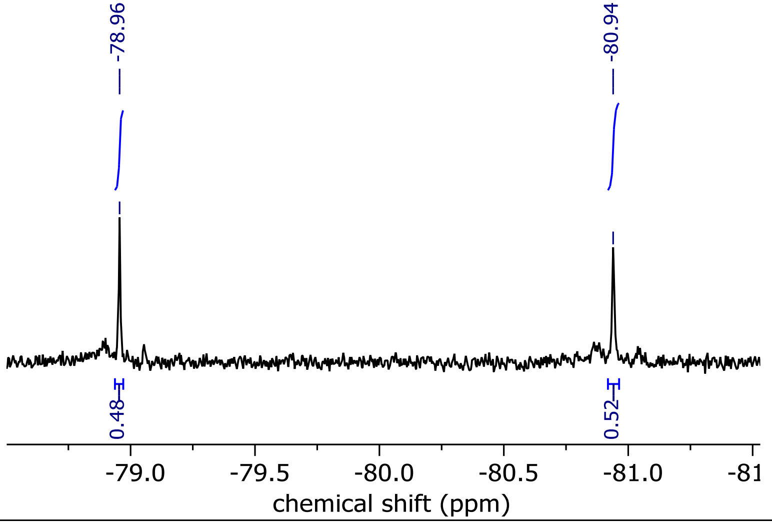

Unmodified oligonucleotides

ON1

Sequence TCC CCC

HRMS (calc. m/z – 842.65379, found m/z – 842.65435)

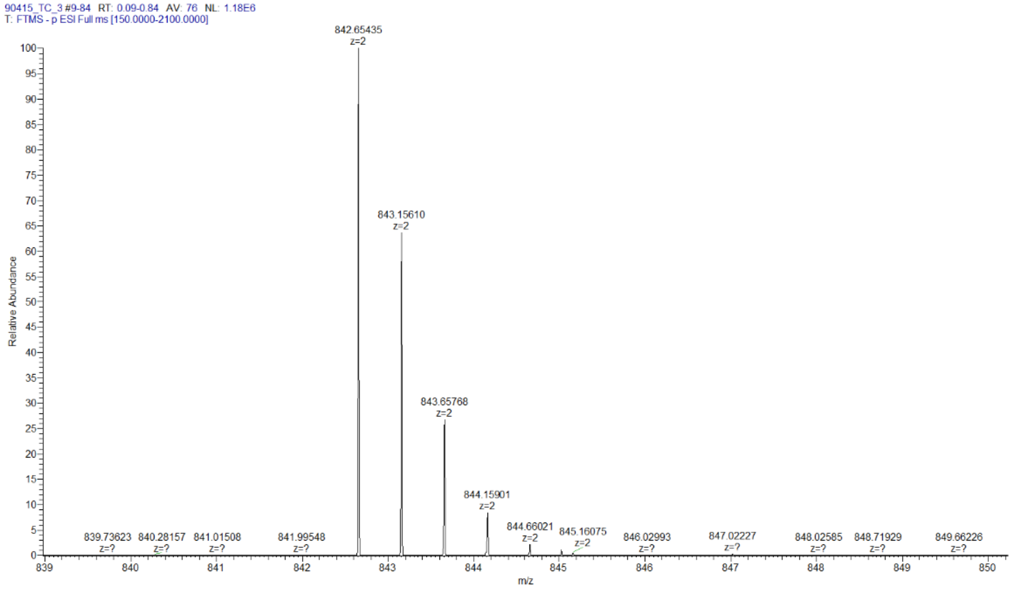

RP HPLC (purified product)

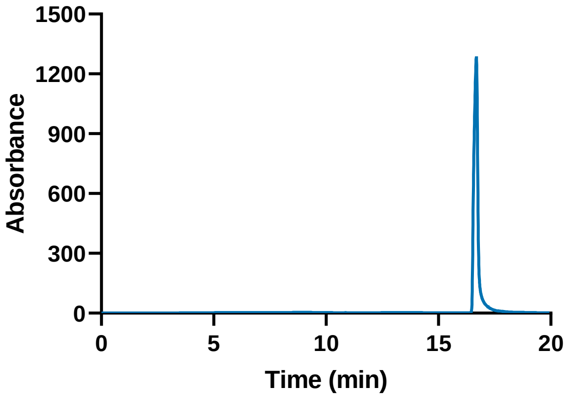

hTeloC

Sequence TAA CCC TAA CCC TAA CCC TAA CCC

HRMS (calc. m/3z – 1780.80415, found m/3z –)

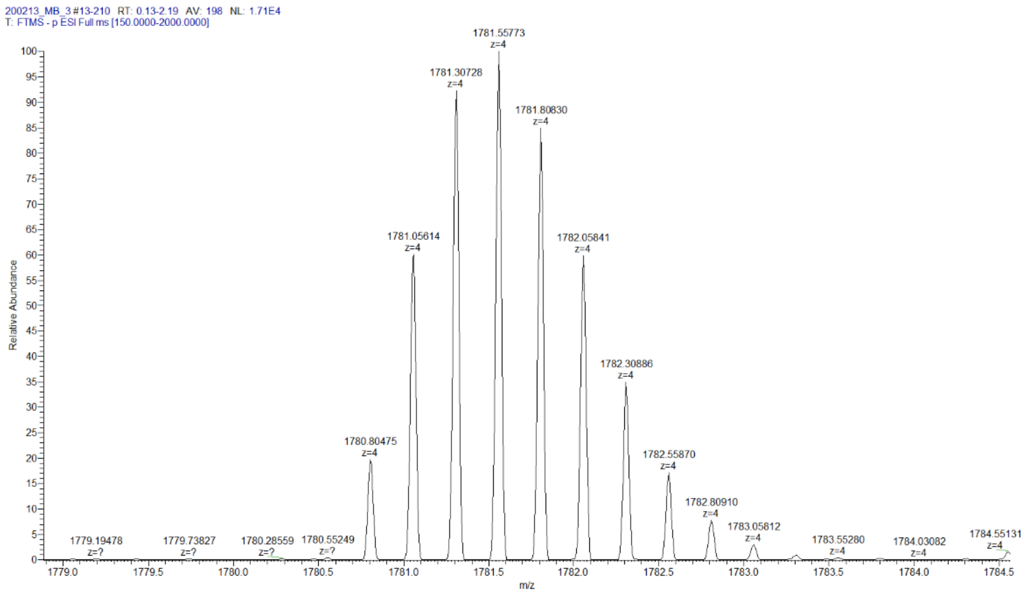

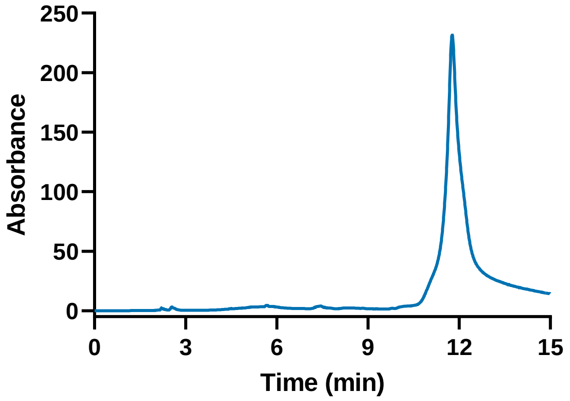

RP HPLC (purified product)

ON3

Sequence AGA CAT TGA C

HRMS (calc. m/z – 1010.51423, found m/z –)

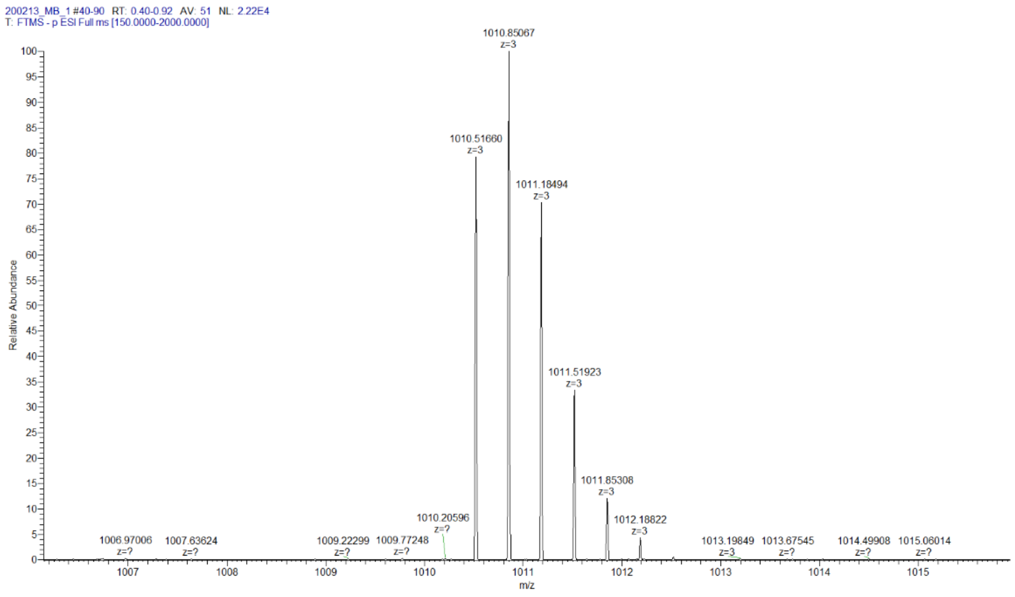

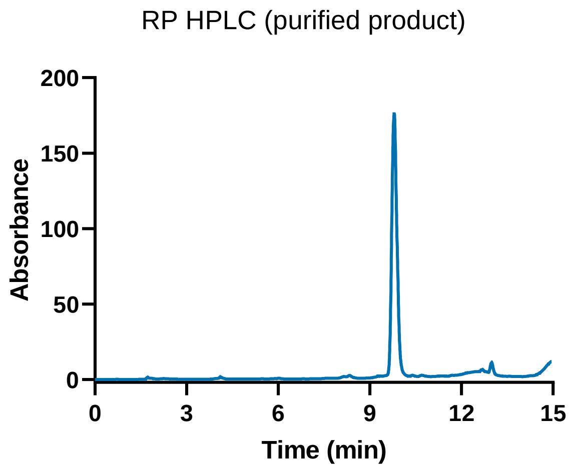

ON4

Sequence TGA CAT TGA C

HRMS (calc. m/z - 1007.51037, found m/z – 1007.51482)

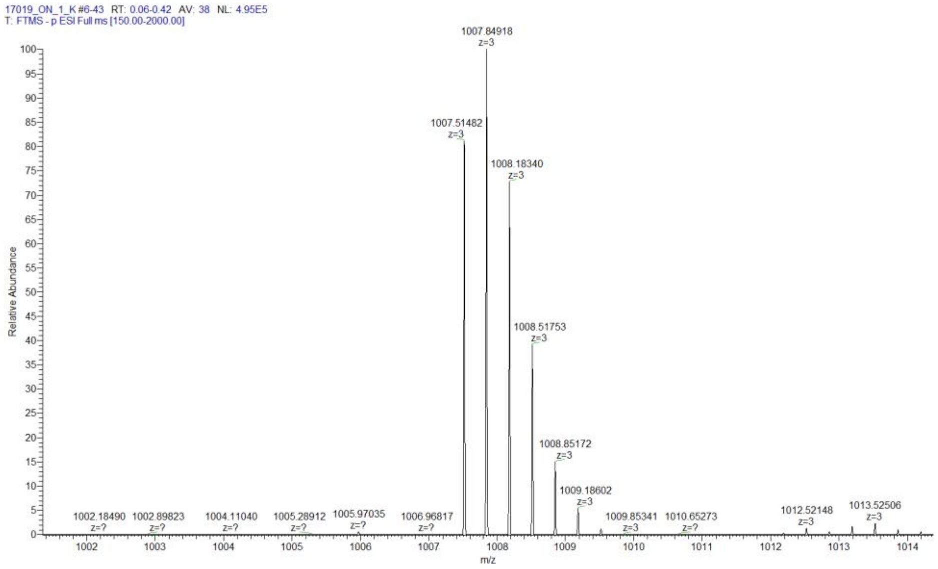

RP HPLC (purified product)

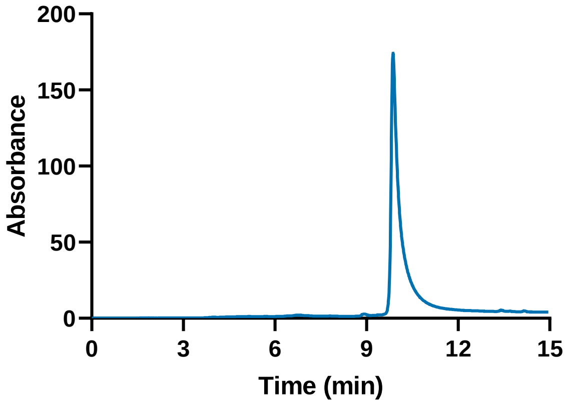

ON5

Sequence GTC AAT GTC C

HRMS (calc. m/z - 999.50662, found m/z – 999.51110)

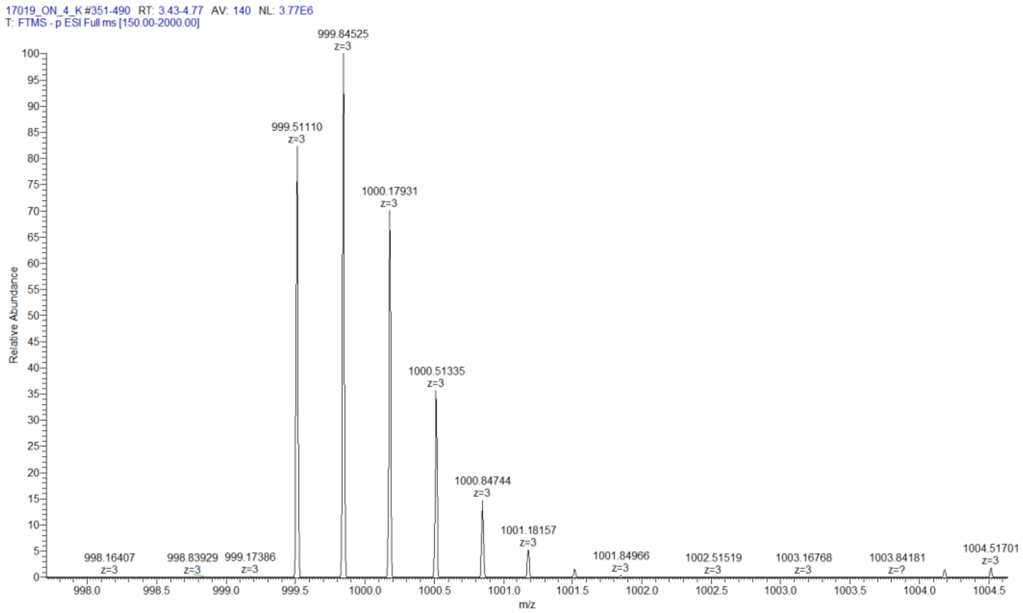

RP HPLC (purified product)

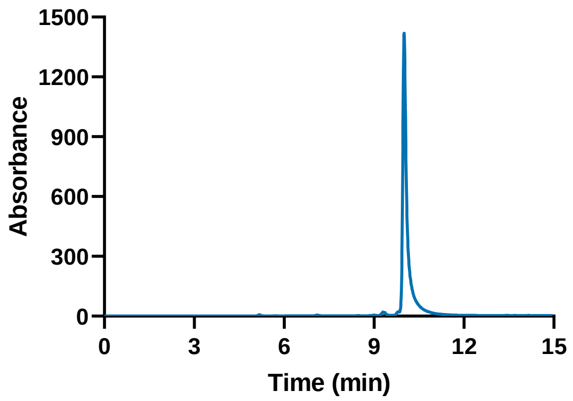

ON9

Sequence GTC AAT GTC T

HRMS (calc. m/z - 1004.50652, found m/z – 1004.51057)

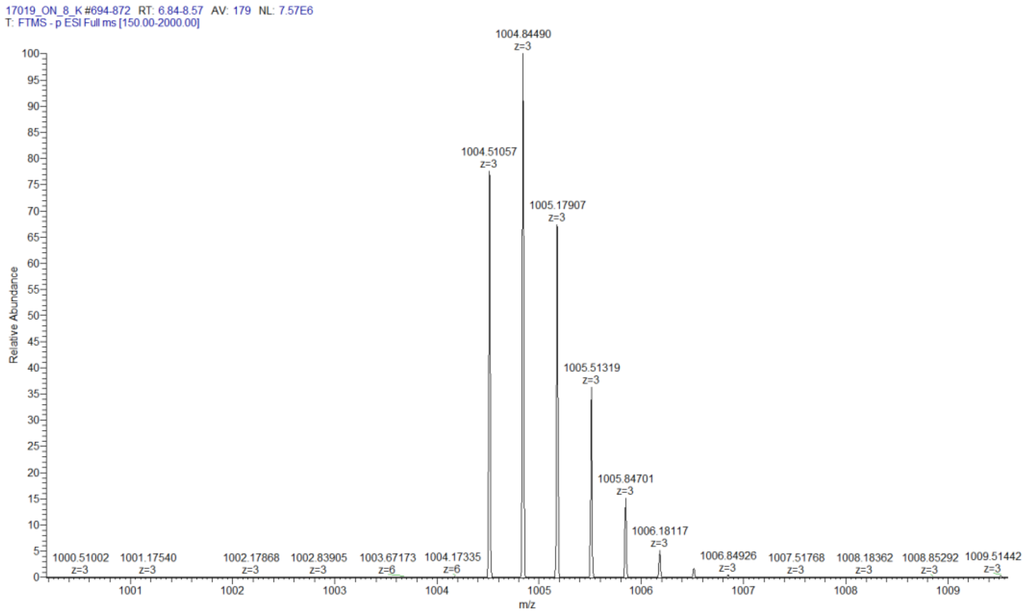

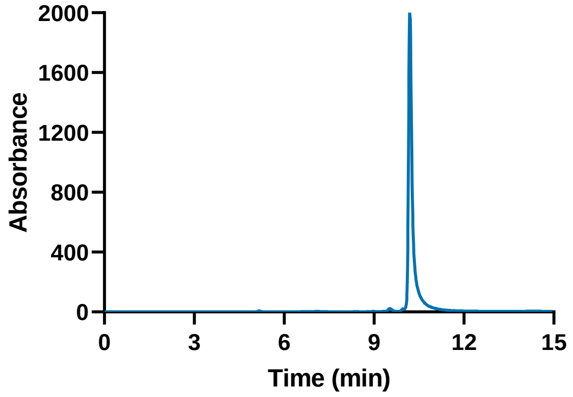

ON11

Sequence GGT TGG TGT GGT TGG

HRMS (calc. m/3z - 1573.59044, found m/3z –)

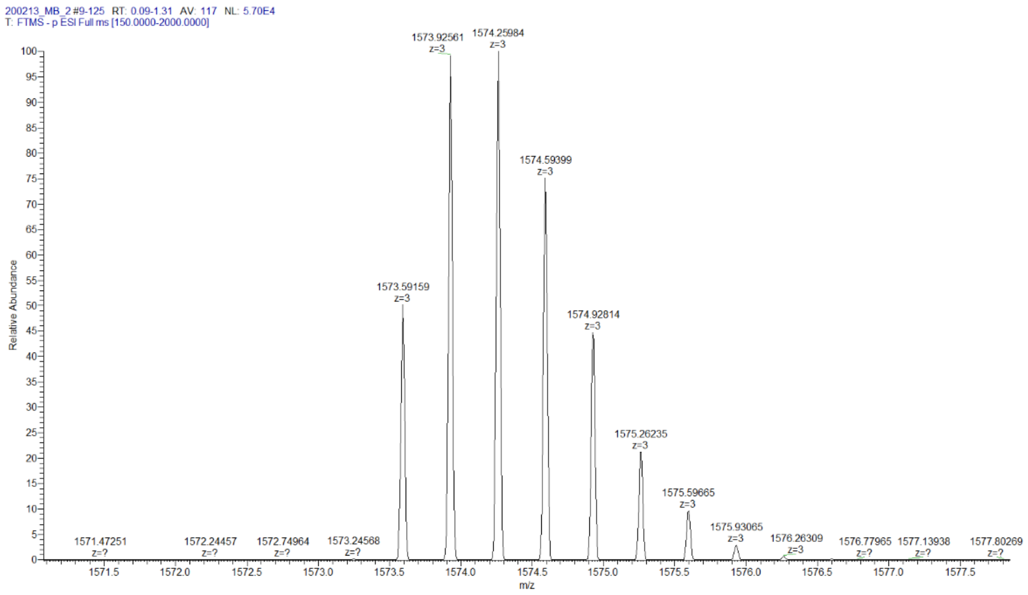

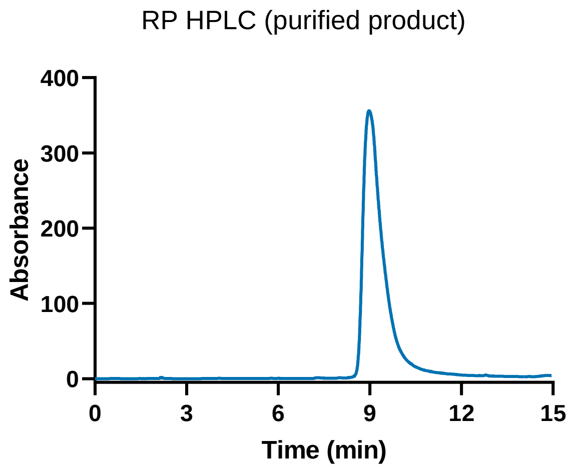

PON11

Sequence p-GGT TGG TGT GGT TGG

HRMS (calc. m/3z - 1600.24588, found m/3z – 1600.24497)

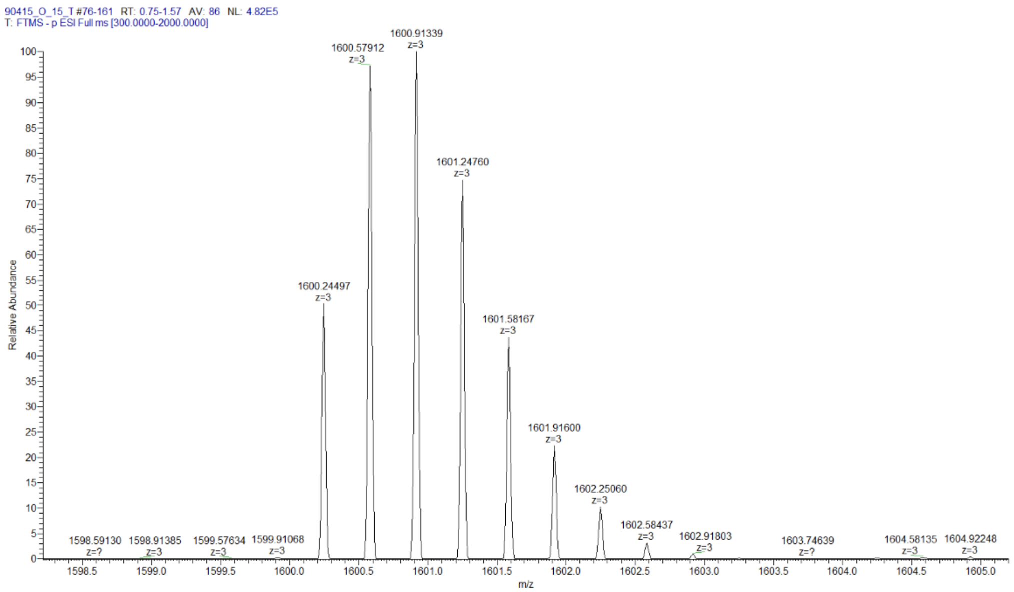

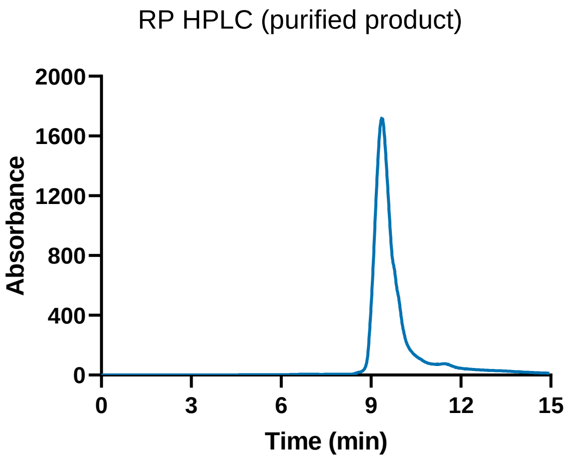

ON20

Sequence GTC AAT GTC G

HRMS (calc. m/z -1012.84201, found m/z -1012.84612)

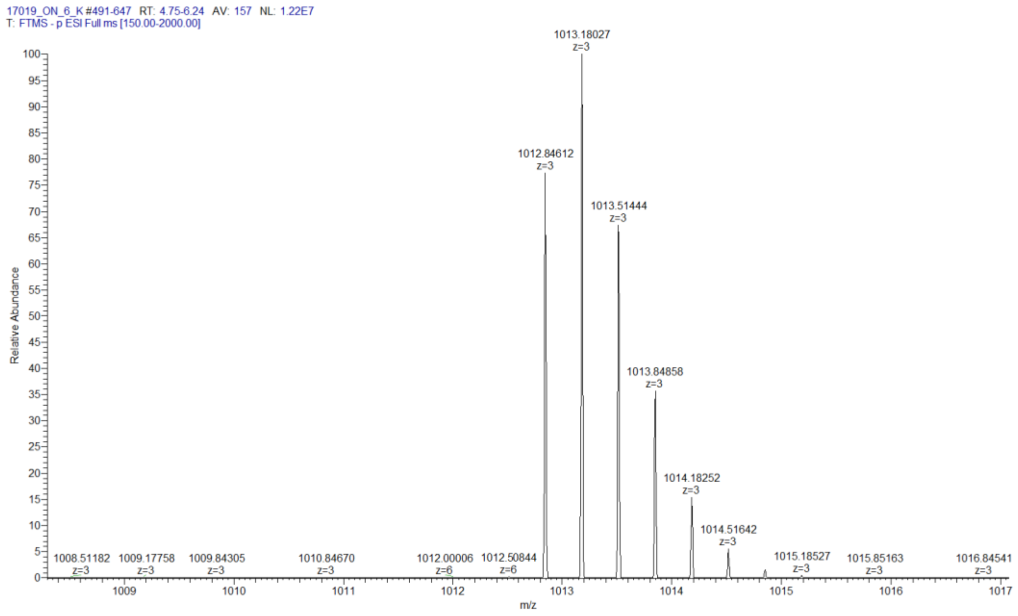

RP HPLC (purified product)

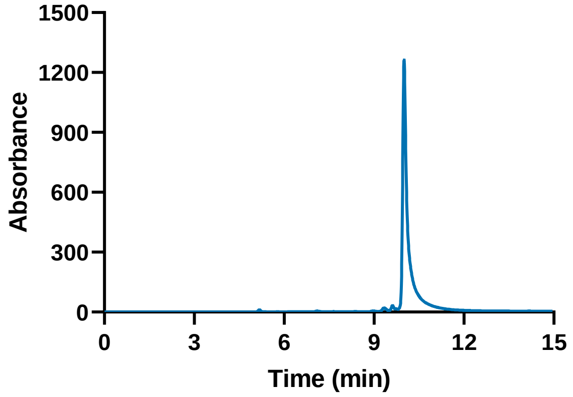

ON21

Sequence GTC AAT GTC A

HRMS (calc. m/z - 1007.51037, found m/z – 1007.51098)

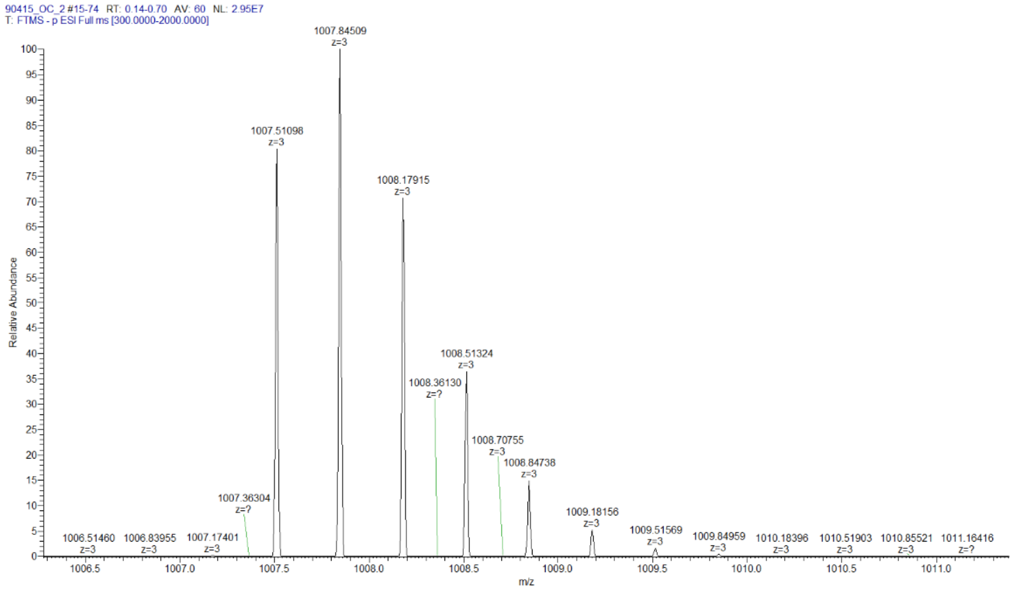

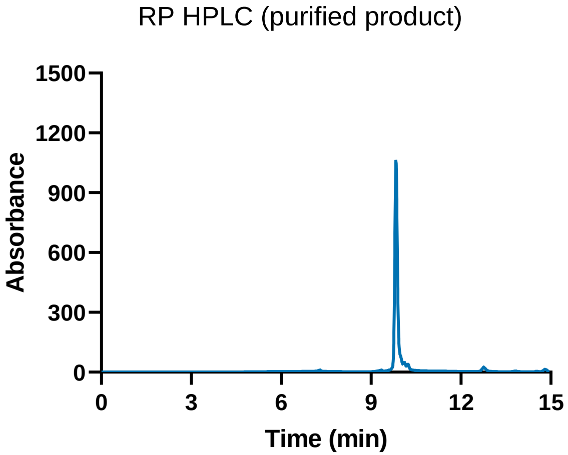

ON22

Sequence GTC AAT GTT T

HRMS (calc. m/z - 1009.50640, found m/z – 1009.50718)

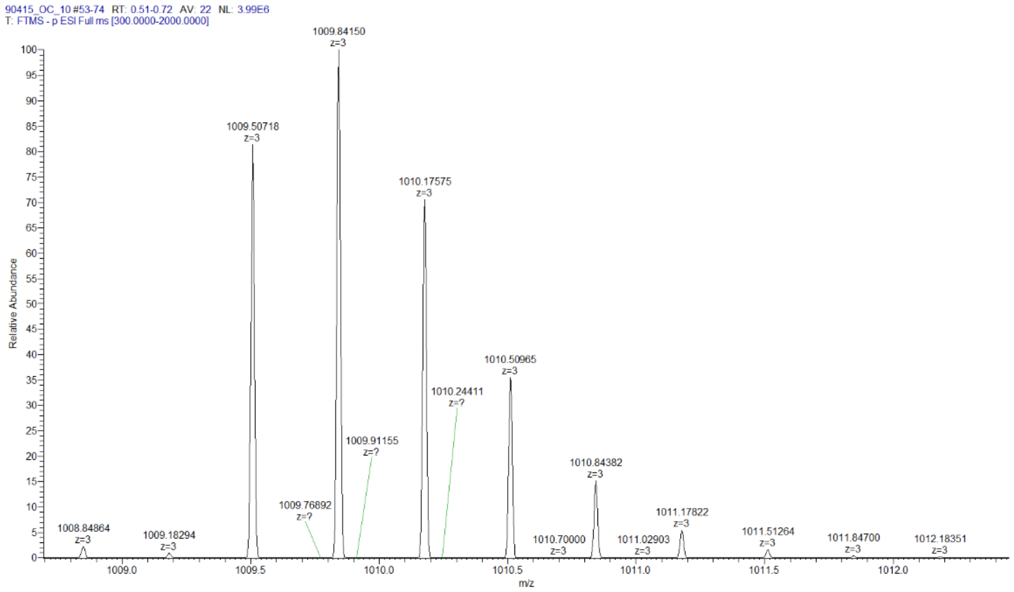

RP HPLC (purified product)

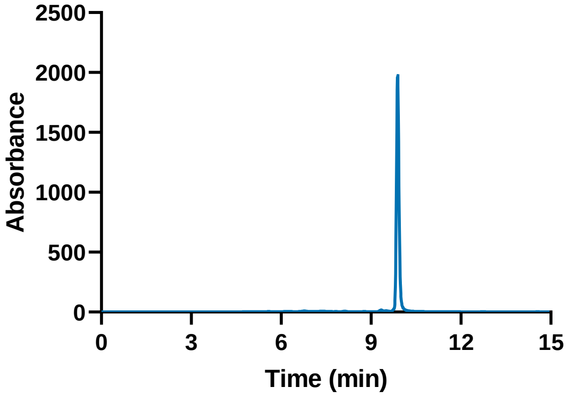

ON23

Sequence GTC AAT GTG T

HRMS (calc. m/z - 1017.84190, found m/z – 1017.84319)

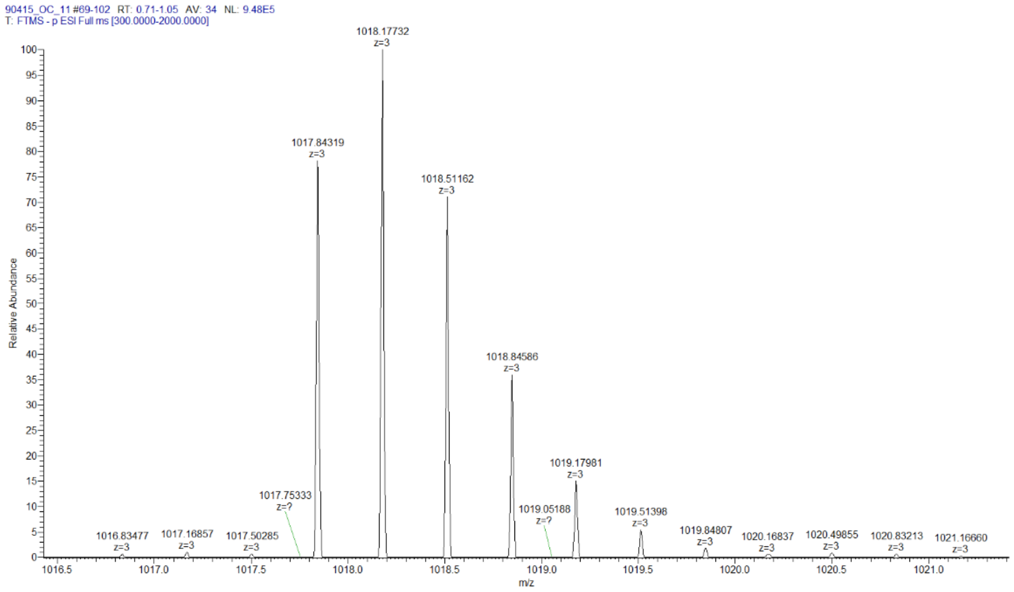

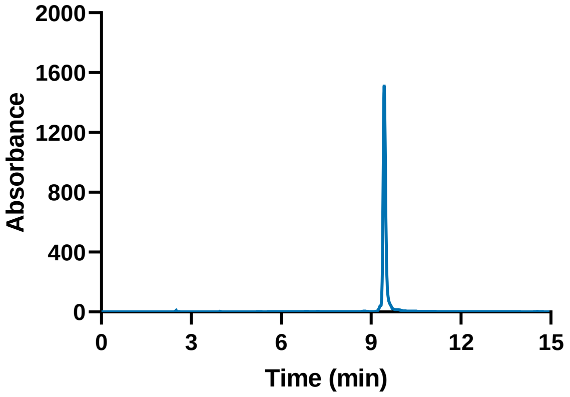

ON24

Sequence GTC AAT GTA T

HRMS (calc. m/z - 1012.51026, found m/z – 1012.51180)

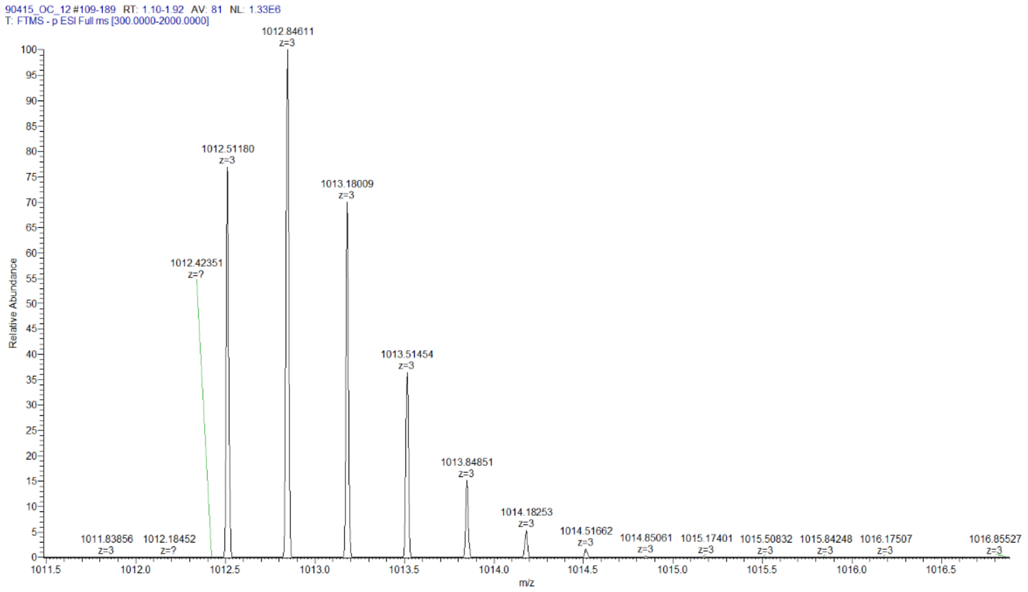

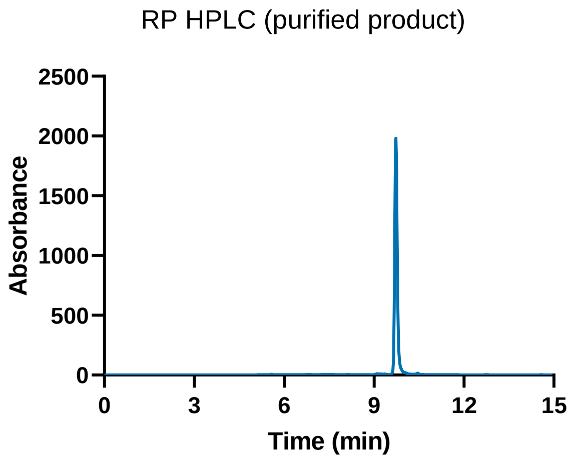

ON25

Sequence TGA CAT TGA T

HRMS (calc. m/z - 1012.51026, found m/z – 1012.51121)

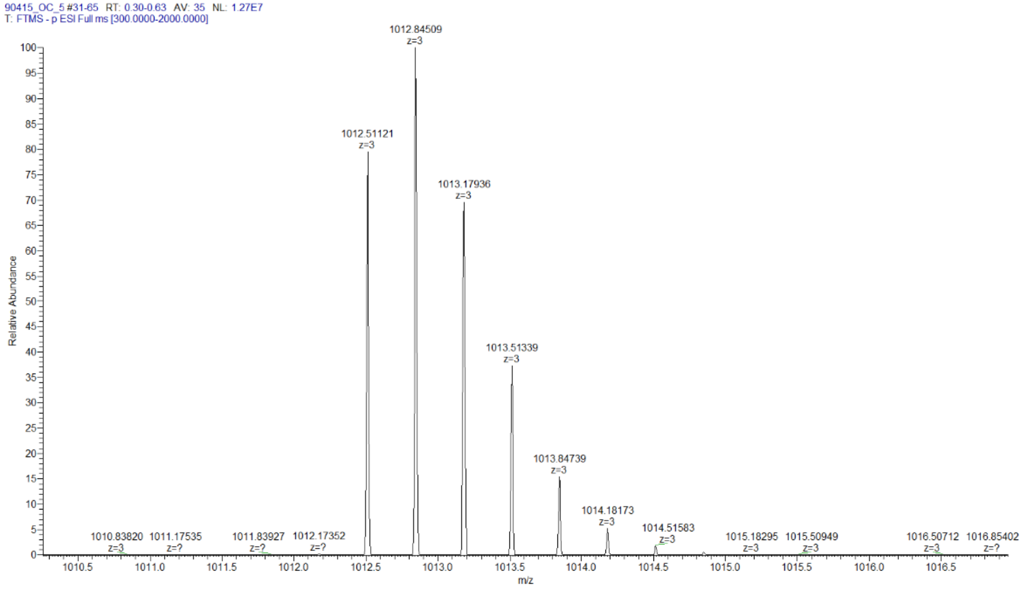

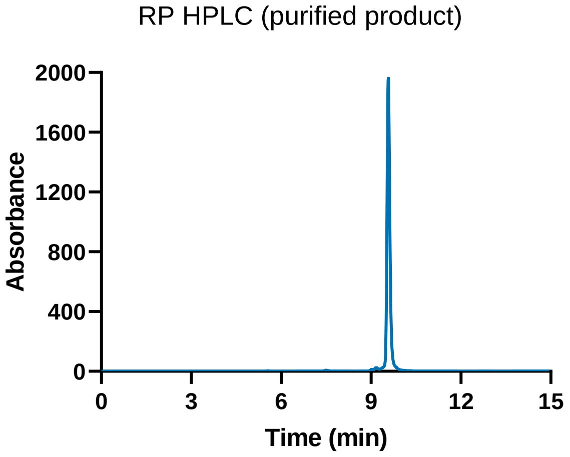

Supplement: gkaa470_Supplemental_Files [file gkaa470_supplemental_files.zip › Supplementary Information 2 - HPLC, HRMS and 19F spectra.pdf]
